# Clinical-mediated discovery of pyroptosis in CD8^+^ T cell and NK cell reveals melanoma heterogeneity by single-cell and bulk sequence

**DOI:** 10.1038/s41419-023-06068-5

**Published:** 2023-08-24

**Authors:** Ying Zhang, Yun Bai, Xiao-Xuan Ma, Jian-Kun Song, Yue Luo, Xiao-Ya Fei, Yi Ru, Ying Luo, Jing-Si Jiang, Zhan Zhang, Dan Yang, Ting-Ting Xue, Hui-Ping Zhang, Tai-Yi Liu, Yan-Wei Xiang, Le Kuai, Ye-Qiang Liu, Bin Li

**Affiliations:** 1 Department of Dermatology, Yueyang Hospital of Integrated Traditional Chinese and Western Medicine, Shanghai University of Traditional Chinese Medicine, Shanghai, 200437 China; 2 Shanghai Skin Disease Hospital, Tongji University, Shanghai, 200443 China; 3 Institute of Dermatology, Shanghai Academy of Traditional Chinese Medicine, Shanghai, 201203 China; 4 Shanghai Applied Protein Technology Co., Ltd., 58 Yuanmei Road, Shanghai, 200233 China; 5 School of Rehabilitation Science, Shanghai University of Traditional Chinese Medicine, Shanghai, 201203 China

**Keywords:** Prognostic markers, Melanoma

## Abstract

Histologically, melanoma tissues had fewer positive cells percentage of pyroptosis-related genes (PRGs), GZMA, GSDMB, NLRP1, IL18, and CHMP4A in epidermal than in normal skin. Pyroptosis, a new frontier in cancer, affects the tumor microenvironment and tumor immunotherapy. Nevertheless, the role of pyroptosis remains controversial, which reason is partly due to the heterogeneity of the cellular composition in melanoma. In this study, we present a comprehensive analysis of the single-cell transcriptome landscape of pyroptosis in melanoma specimens. Our findings reveal dysregulation in the expression of PRGs, particularly in immune cells, such as CD8^+^ cells (representing CD8^+^ T cells) and CD57^+^ cells (representing NK cells). Additionally, the immunohistochemical and multiplex immunofluorescence staining experiments results further confirmed GZMA^+^ cells and GSDMB^+^ cells were predominantly expressed in immune cells, especially in CD8 ^+^ T cells and NK cells. Melanoma specimens secreted a minimal presence of GZMA^+^ merged CD8^+^ T cells (0.11%) and GSDMB^+^ merged CD57^+^ cells (0.08%), compared to the control groups exhibiting proportions of 4.02% and 0.62%, respectively. The aforementioned findings indicate that a reduced presence of immune cells within tumors may play a role in diminishing the ability of pyroptosis, consequently posing a potential risk to the anti-melanoma properties. To quantify clinical relevance, we constructed a prognostic risk model and an individualized nomogram (C-index=0.58, *P* = 0.002), suggesting a potential role of PRGs in malignant melanoma prevention. In conclusion, our integrated single-cell and bulk RNA-seq analysis identified immune cell clusters and immune gene modules with experiment validation, contributing to our better understanding of pyroptosis in melanoma.

## Introduction

Cutaneous melanoma, characterized by its high mortality rates [1], exemplifies the paradox of possessing both high antigenicity and strong immunoevasive properties [2, 3]. The proposition that overcoming tumor-induced immunosuppression is crucial for the success of immunotherapies is supported by the remarkable clinical responses observed in anti-melanoma treatments utilizing anti-CTLA4/PD-1/PD-L1 antibodies [4]. However, despite the significant advancements made in immune checkpoint inhibitors (ICIs), a considerable proportion of melanoma patients still do not exhibit positive responses to ICI-based therapies [5]. Approximately 71.4% of metastatic melanoma patients are resistant to immunotherapy [6].

Considerable evidence suggests that the heterogeneity of cutaneous melanoma is a major cause of treatment failure. Overall survival (OS) rates vary widely which range from 14% to 92% for melanoma patients [7, 8]. The ecological milieu of melanoma encompasses a diverse array of cellular components, encompassing malignant, immune, and stromal entities [9]. Hence, studies focusing on the cellular level will add greater clarity and value to current findings, which could promote personalized and precision medicine, with critical implications.

**Fig. 1:**
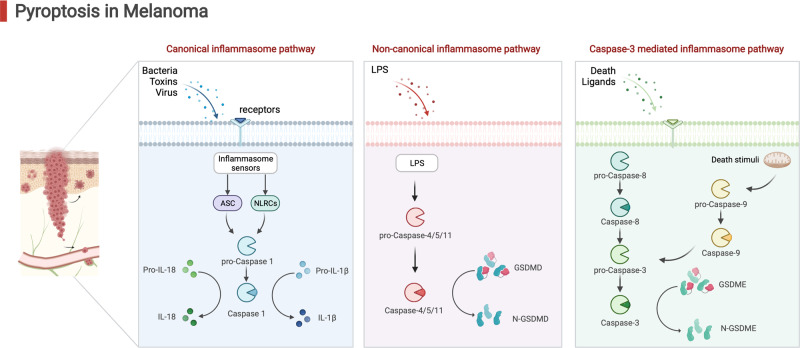
An overview of the pathways involved in pyroptosis.

Recently, tumor pyroptosis is recently been regarded as a promising strategy for tumor treatment [10, 11] (Fig. 1). Pyroptosis is a kind of programmed cell death (PCD), the classic pyroptotic pathway which is triggered by the activation of leucine-rich repeat family protein 3 (*NLRP3*) inflammasomes of the nucleotide-binding domain and is associated with an inflammatory response [12, 13]. Of importance, pyroptosis is highly correlated with the modulation of immunity in the tumor microenvironment [14].

So far, there has been little agreement on the role of pyroptosis in cancer research [10, 15]. Pyroptosis is still a mystery, with conflicting findings, despite all of the Gasdermin (GSDM) proteins being studied in cancer [15]. On the one hand, as a consequence of cell pyroptosis, inflammatory substances are expelled from tumor cells, creating an inflammatory microenvironment that promotes tumor growth [16–18]; On the other hand, inducing cancer cell pyroptosis is another way to reduce tumor load [19–21].

The reason why pyroptosis leads to two opposing consequences were modified by different cell source [15, 22, 23]. Traditionally, the prior studies solely selected the mRNA levels on different samples [21, 24, 25]. However, the above evidence was not enough to determine the cell lines of pyroptosis and assess salient tumor heterogeneity. Compared with these studies, we aim to assess salient cellular heterogeneity.

Single-cell sequencing (scRNA-seq) offers an unparalleled opportunity to dissect the tumor microenvironment of melanoma, enabling the exploration of immune context and tumor heterogeneity [26–28]. As tumor-associated cell types are increasingly studied using scRNA-seq, the abundance and functional state of these cells are being characterized and have provided detail of cellular composition heterogeneity [29]. With single-cell genomics approaches, genomic features can be evaluated in 100s–2000s of individual tumor cells at a time [30, 31]. This approach is capable of identifying all major components of the cell simultaneously and determining their molecular and genomic characteristics [32], and determining which of these features may be predictive or explicative of the heterogeneous.

In this study, we performed integrated single-cell and bulk RNA-seq analysis to identify immune cell clusters and immune gene modules. We performed in vitro cell experiments to further explore a specific mechanism between the expression of the pyroptosis-related genes (PRGs) on different immune cells and verify pyroptosis expression using immunohistochemical (IHC) and multiplex immunofluorescence staining experiments. Our findings are expected to provide newer insights at the cellular level into the role of pyroptosis for individualized diagnosis or treatment for melanoma patients.

## Materials and methods

### Patients and ethics approval

All clinical specimens utilized in this study were obtained from the Shanghai Skin Disease Hospital, following the necessary informed consent for research purposes and approval from the Ethics Committee of the same hospital (No. 2021-078), in strict adherence to the principles outlined in the Declaration of Helsinki. The melanoma samples consisted of superficial spreading melanoma, ocular melanoma, freckled nevus melanoma, and amelanotic melanoma samples, which were diagnosed with melanoma by the Shanghai Skin Disease Hospital. Control skin samples were procured from excess skin acquired during cosmetic surgeries. All melanoma samples were reconfirmed by two independent pathologists from the Department of Clinical Pathology of the hospital. A total of nine melanoma patients were obtained and their clinical attributes were presented in Table S1. Subsequently, the samples were promptly fixed in 70% formalin upon excision and subjected to paraffin embedding for further processing.

### Pyroptosis related genes identification

As pyroptosis has been annotated as one of the major routes in melanoma patients, the current study designs a series of bioinformatics analyses and experiments to identify potential PRGs and validate the function clinically. We obtained PRGs from the Gene Set Enrichment Analysis database. A total of 471 melanoma and clinical samples’ transcriptome RNA sequence data (Table 1) were acquired from the Cancer Genome Atlas (TCGA) database. Exploring the GTEx portal, 1089 samples were collected from non-diseased tissue. In detail, we use OS as the initial screening condition, which has been considered the gold standard for the demonstration of clinical benefit [33, 34]. Attempting to select the potential PRGs, we performed bioinformatics analysis, including Kaplan-Meier (K-M) based OS calculation and protein-protein interaction (PPI) analysis based on MCODE and CytoNCA analysis to identify the potential PRGs.

**Table 1:** Clinical characteristics of bulk seq.

	Characteristic	patients
Status	Alive	247
	Dead	217
Age	Mean (SD)	58.2 (15.7)
		58 [15,90]
Gender	Female	180
	Male	291
T stage	T0	23
	T1	42
	T2	78
	T3	91
	T4	153
N stage	N0	235
	N1	74
	N2	49
	N3	56
M stage	M0	418
	M1	25
TNM stage	0	7
	I	77
	II	140
	III	171
	IV	24
New tumor event type	Metastasis	167
	Metastasis and recurrence	6
	Primary	19
	Recurrence	52
Therapy type	Chemotherapy	72
	Immunotherapy	74
	Targeted molecular therapy	12
	Vaccine	22

### PRGs protein expression detected by ELISA

Human CHMP4A ELISA-kit (CSB-CL883630HU2, CUSABIO), human GSDMB ELISA-kit (PH103310, PYRAM), human GZMA ELISA-kit (ab225728, Abcam), human IL18 ELISA-kit (ab215539, Abcam), and human NLRP1 ELISA-kit (CSB-EL015864HU, CUSABIO) were used to monitor the kernel PRGs’ protein content.

### Immunohistochemical

IHC staining analysis was conducted using standard immunoperoxidase staining procedures to assess the protein expression of PRGs. IHC staining was implemented as previously described [35]. The following antibodies were employed for the analysis: anti-CHMP4A (bs-7744R, Bioss, Beijing, China, diluted 1:400), anti-NLRP1 (bs-6854R, Bioss, Beijing, China, diluted 1:200), anti-Granzyme A (ab29205, Abcam, Shanghai, China, diluted 1:100), anti-IL-18 (BA11806, Boster, CA, USA, diluted 1:300), and anti-GSDMB (12885-1-AP, Proteintech, Rosemont, USA, diluted 1:600). Each sample of the negative control was diluted and incubated with the same type of antibody under the same experimental conditions. Images were analyzed using the image processing software Image J. Two researchers captured images at a magnification of ×200 using an Olympus BH2 Upright Metallurgical Microscope in order to assess both the morphology and quantity of cells. A sample was made by averaging the positively stained area in three times stanning images of each specimen.

### Single-cell data processing

ScRNA-seq enhances understanding of the functional specificity of cancer cells, leading to the identification of PRGs expression in melanoma by the distribution of different cells. In the current study, the profiles of individual cells from patient-derived melanoma cells and normal cells (GEO database, GSE108394, GSE215120, and GSE180885) were obtained, including 126987 cells. R package ‘Seurat’ and harmony algorithm were used to merge sample files among variables with the criteria of >10% mitochondria [36]. Subsequently, merged data of cells were clustered into 12 cell populations under the condition (resolution = 0.5) after the reduction of cell clustering of the tSNE algorithm. Next, cell populations were annotated using the signatures from the original publication [37]. The PRGs’ distribution and expression level of different cell populations were displayed.

### Cell developmental trajectory

The cell lineage trajectory of immune clusters was inferred using Monocle2. Initially, the “relative2abs” function in Monocle2 was employed to convert TPM into normalized mRNA counts, and an object was created with the parameter “expressionFamily = negbinomial.size” following the Monocle2 tutorial. The “differentialGeneTest” function was utilized to identify differentially expressed genes (DEG) within each cluster, with genes having a q-value < 1e5 being used to order the cells in pseudo time analysis. Subsequently, the constructed cell trajectories were examined for differentially expressed genes along the pseudotime using the “differentialGeneTest” function.

### WGCNA immune module

To quantify the infiltration scores of immune cells in bulk sequence, the immune cell infiltration was performed using the CIBERSORT algorithm. Subsequently, the weighted gene co-expression network (WGCNA) was used when detecting co-expressed gene modules of immune infiltration results to investigate the association between hub genes and immune phenotype. Clustering was performed using a soft threshold power of 4 and a height of 0.25 as the threshold power, with the minimum module size at 30. Using WGCNA, we found modules of strongly correlated genes and calculated the principal component, whose correlation coefficient with immune cell infiltration was calculated.

### Multiplex immunofluorescence staining

The multispectral immunofluorescence (IF) staining method was performed as previously reported [38]. The samples were stained using the following antibodies: anti-CD8 (ZCIA055, zuochengbio), anti-CD57 (ZCIA273, zuochengbio), anti-GZMA (ab209205, Abcam), and anti-GSDMB (12885-1-AP, ThermoFisher). The samples were mounted using ProLong Diamond Antifade mounting medium containing DAPI (Invitrogen) and then sliced into 5 μm sections before being placed onto adhesive microscope slides. We performed deparaffinization, rehydration, and antigen retrieval to prepare the slides for multiplexed immunofluorescence staining. TG TSA Multiplex IHC Assay Kits (TissueGnostics Asia-Pacific Ltd.) were used along with a spectral library constructed from single-staining tissue images of each reagent to guide the staining process. The imaging procedure was performed utilizing the TissueFAXS (TissueGnostics) system, in conjunction with the Zeiss Axio Imager Z2 Microscope System, at a magnification of ×20. Quantitative analysis of the stained samples involved measuring cell density, expression levels, and area per cell using StrataQuest software (version 7.1.129, TissueGnostics GmbH, Vienna, Austria).

### Clinical Relevance and pyroptosis prognostic nomogram

To provide a quantitative analysis tool to estimate the individual survival risk of melanoma, a nomogram was constructed based on PRGs and clinical predictors, integrating the prognostic signature of 1, 2, and 3-year OS of melanoma patients. The results of multivariate prognostic analysis for PRGs were acquired by application of the R package ‘forest plot’. The R package “survival” and “survminer” was employed to perform K-M analysis, followed by 1-, 2-, and 3-year OS predicting performance using the R package “survivalROC”. Considering assessing the nomogram’s predictive performance, the concordance index (C-index) and calibration curves were drawn for comparison of the predictive and actual survival times. R package ‘rms’ was used to plot the nomogram and calibration curve, contributing to evaluating the OS and progression survival rate on kernel PRGs.

### Statistical methods

A GraphPad Prism 8 program was used to analyze the experimental validation data by three times experiments replicated. Mean ± standard deviation (SD) is the unit of measurement. Statistical significance was determined by a p-value of 0.05 on the two-side t-test for comparing the groups when the data meet the assumption of normal distribution, and the variance is similar between the groups that are being statistically compared.

## Results

### Melanoma tissues had fewer PRGs positive cells in the epidermal clinically

**Fig. 2:**
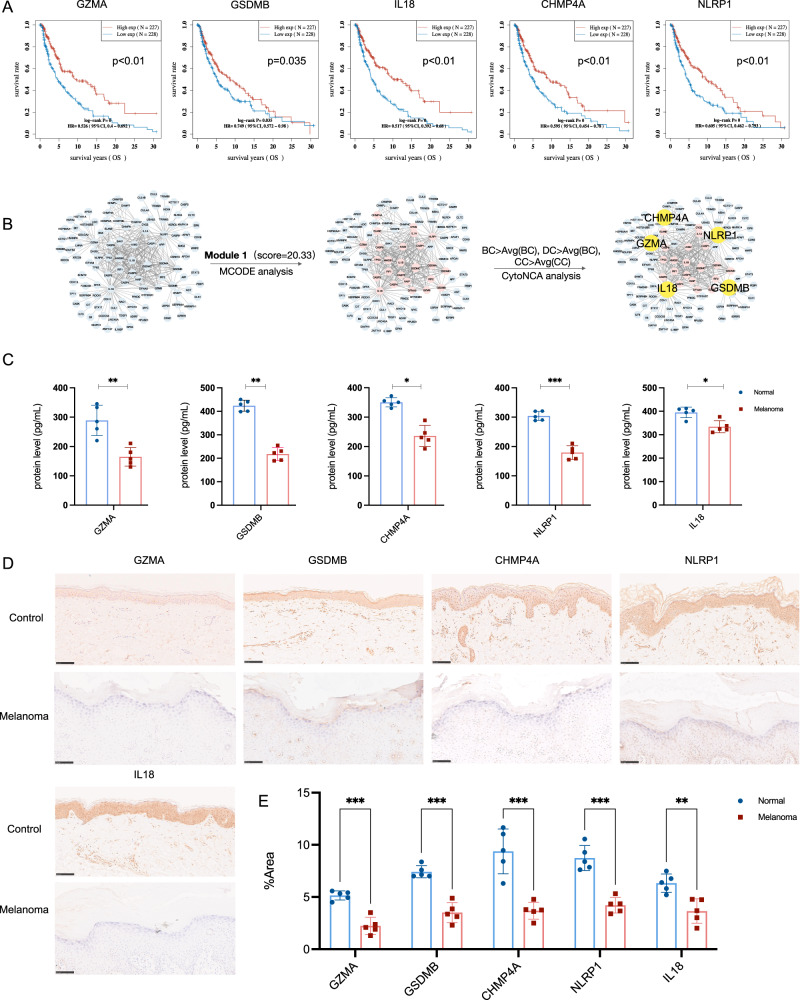
Melanoma specimens reduce PRGs positive cells in epidermal clinically. **A** Overall survival analysis based on K-M was calculated to identify the potential PRGs clinically. **B** PPI via MCODE and CytoNCA algorithm was established, in which PRGs were screened out as central protein functioning in melanoma deficiency. **C** GZMA, GSDMB, CHMP4A, NLRP1, and IL18 protein expression of melanoma and control patients detected by ELISA. *p < 0.05, **p < 0.01, ***p < 0.001, ****p < 0.0001, *n* = 5, compared with the normal group. **D** Representative IHC stanning of GZMA^+^, GSMDB^+^, CHMP4A^+^, NLRP1^+^, and IL18^+^ in melanoma specimens from the clinic. Scale bar = 100 μm, *n* = 5 samples per group. **E** The quantification of PRGs protein in epidermal areas, *p < 0.05, **p < 0.01, ***p < 0.001, ****p < 0.0001, compared with the control group.

As pyroptosis has been annotated as one of the major routes in melanoma patients, the current study designs a series of bioinformatics analyses and experiments to identify potential PRGs and validate the function clinically. Attempting to evaluate the landscape of pyroptosis and identify potential PRGs, we performed OS analysis based on K–M and PPI via MCODE and CytoNCA algorithm. In detail, the K–M survival curves were plotted between high expression (*n* = 227) and low expression (*n* = 228) of PRGs (Fig. 2A), which indicated that only *GZMA*, *GSDMB, NLRP1*, *CHMP4A*, and *IL18* reached significant better OS (*p* < 0.05), which has been considered as the gold standard for demonstration of clinical benefit [33]. Consistently, the PPI analysis reveals that the above five pyroptosis has been identified as the core PRGs in module 1 (score=20.33) (Fig. 2B). Therefore, we aim to probe the function of GZMA, GSDMB, NLRP1, CHMP4A, and IL18 in the following experiments.

To validate the activation and function of PRGs, we detected the protein level in melanoma patients and control volunteers. The initial findings from the enzyme-linked immunosorbent assay (ELISA) indicated a statistically significant downregulation of pyroptosis protein expression in melanoma patients when compared to the control group (Fig. 2C), especially NLRP1, GZMA, and GSDMB, followed by CHMP4A and IL18. IHC staining analysis (melanoma and control groups, *n* = 5, respectively) was employed to ascertain that melanoma reduced pyroptosis protein expression in epidermal areas (Fig. 2D, E). Histologically, GSDMB, NLRP1, and CHMP4A were primarily localized in the cytoplasm rather than the nucleus, while GZMA and IL18 were mainly localized in the nucleus, and IL18 was mainly localized in the cytoplasm after activation. In combination, the findings suggested that GZMA, GSDMB, NLRP1, CHMP4A, and IL18 deficiency, at least in part, are responsible for melanoma. Although the clinical evidence implied that melanoma skin tissue reduced GZMA, GSDMB, NLRP1, IL18, and CHMP4A expression in epidermal, however, the result obtained with bulk-sorted samples could not explain the main scientific questions on cell sources heterogeneity.

### ScRNA-seq of the ecosystem of melanoma by deep learning

**Fig. 3:**
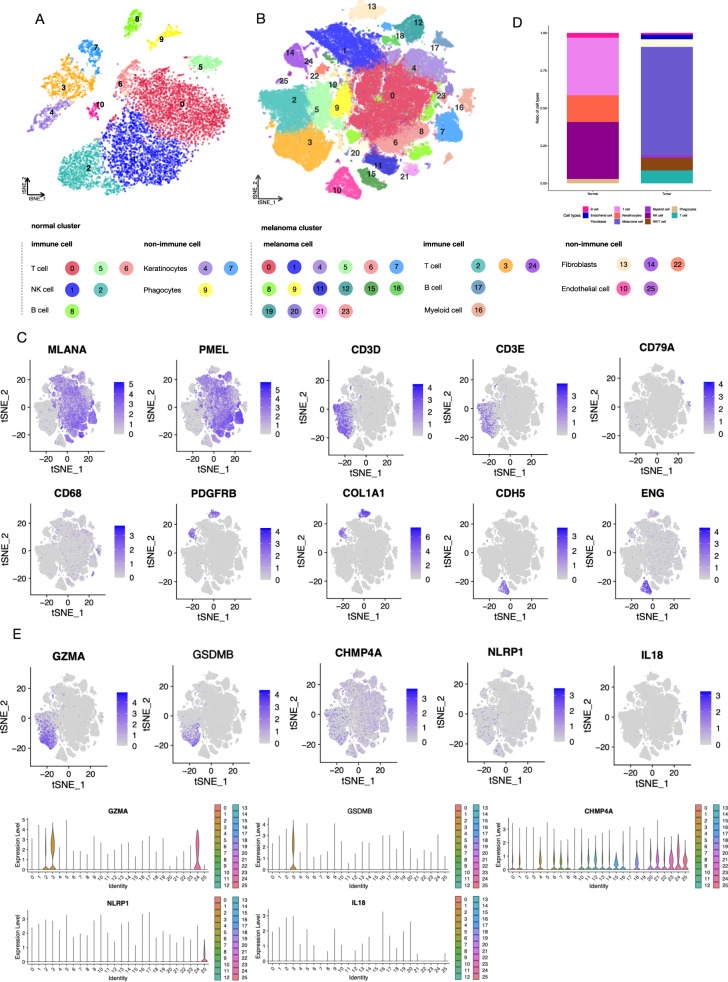
scRNA-seq profiling of the melanoma environments. **A** T-distributed stochastic neighbor embedding (tSNE) plot of control samples, non-immune cells, and immune cells are shown separately. **B** tSNE plot of melanoma samples, showing the annotation and color code for cell types in the melanoma ecosystem and cell origins by color, non-immune cells, and immune cells are shown separately. **C** The cell classification basis of melanoma scRNA-seq analysis was displayed with the marker genes expression in the Feature plot. **D** Histogram indicating the proportion of cells in tumor tissue of each group. **E** The expression of kernel PRGs in melanoma indicates the origination of immune cells.

In order to investigate the cellular source of the kernel PRGs, we performed scRNA-seq with a total of 126987 cells (Fig. 3A, B). We identified 25 distinctive cell clusters in melanoma and grouped melanoma tumor cells into melanoma cells, immune cells (T cell, B cell, and Myeloid cell), and non-immune cells (fibroblast and endothelial cell). The markers utilized in the conducted study, as defined by Tirosh and colleagues, successfully differentiated between various cell types [23, 39], including melanoma cell (*MLANA, PMEL*), T cell (*CD2, CD3D, CD3E, CD4, CD8A*). NK/T cell (*NKG7, GNLY*), B cell (*MS4A1, CD79A, CD79B, CD19*), myeloid (*CD68, CD163*), fibroblast (*PDGFRA, PDGFRB, COL1A1*), and endothelial cell (*CDH5, ENG, PECAM1*). Shows in Fig. 3C, the representative expression feature plots of cell classification basis markers were displayed, respectively.

We observed that melanoma and control samples were distinguishable by the subgroups of the immune cells (Fig. 3D), especially T cells, B cells, and myeloid cells. Notably, our investigation into the cell types from which the PRGs secreted revealed that the five PRGs were predominantly expressed in immune cells, particularly in T cell-related subgroups (Fig. 3E). For instance, the presence of *GZMA* and *GSDMB* was observed in T cell subgroups (cluster 2, 3, 24). The above evidence leads to a requirement for isolating immune cells and identifying immune subgroups.

### Immune microenvironment of melanoma

**Fig. 4:**
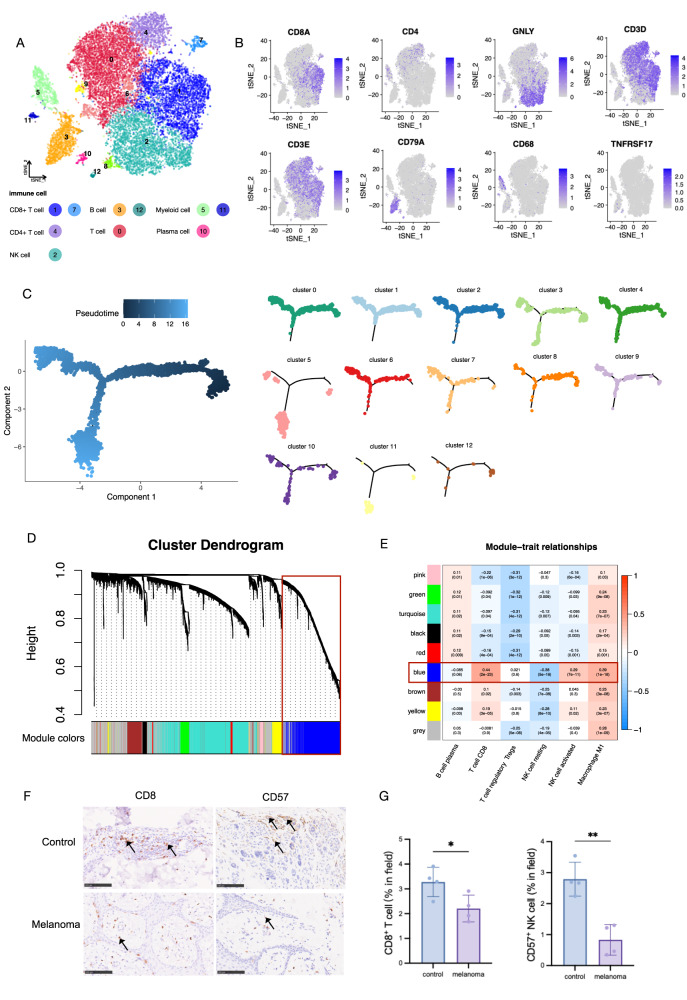
scRNA-seq profiling of immune cell components in melanoma. **A**. t-SNE plot showing the clusters of immune cells and cell origins by color, according to immune cell types. **B** The cell classification basis of immune cell scRNA-seq analysis was displayed with the marker genes expression in Feature plot. **C** Pseudotime-ordered analysis to construct the developmental trajectories of the isolated immune cell subgroups. **D** WGCNA in bulk sequence. Gene cluster dendrogram clustered by weighted gene co-expression network in different colors. **E** The heatmap of module-trait relationships. The number in and outside the bracket represents the *P*-value and Pearson coefficient, respectively. **F** Representative IHC stanning of CD8^+^ and CD57^+^ in immune cell from the clinic. Scale bar = 100 μm, *n* = 4 samples per group. **G** The quantification of PRGs protein in epidermal areas, *p < 0.05, **p < 0.01, ***p < 0.001, ****p < 0.0001, compared with the control group.

The 18295 cells derived from cell clusters that were annotated as immune cells (Fig. 4A) were partitioned into 12 clusters and subsequently classified into 7 distinct cell types. These identified cell clusters exhibited characteristic marker genes (Fig. 4B). The T cell cluster consisted of two CD8^+^ T cell subgroups (cluster1, 7), one CD4^+^ T cell subgroup (cluster 4), one NK cell subgroup (cluster 2), and one non-annotated T cell subgroup (cluster 0), together with the myeloid cell subgroup (cluster 5,11), and plasma cell subgroup (cluster 10). The CD8^+^ T cell subgroups highly expressed *CD8A*, especially in cluster 1, and the CD4^+^ T cell subgroup with significant *CD4* expression. The NK cell subgroup was naïve T cells, which were marked with an expression of the *GNLY* gene. The expression and distribution plot of myeloid cells and plasma cells are also displayed in Fig. 4B.

Monocle [40] was utilized in order to construct the developmental trajectories of the isolated immune cell subgroups. The results indicate that the clusters mentioned earlier exhibit varying states. The immune cells from subgroups 0, 1, and 2 predominantly occupy the root position in the phylogenetic tree. This observation suggests that cells from the CD8^+^ T cell and NK cell subgroups are likely to be less differentiated and more primitive, which may confer an advantage in terms of immune protection against melanoma. This is illustrated in Fig. 4C.

Consistently, the immune cell expression of bulk-seq was also calculated to provide larger-scale clinical evidence. The WGCNA analysis was performed to identify the characteristic gene expression network nodule that discriminated immune cell height most from the others (Fig. 4D). Those modules were ranked according to the *coef* to prioritize which co-expressed genes were important in the discrimination. As a result, the blue module ranked first, where closed with CD8^+^ T cells (coef=0.44[2e–23]), followed by NK cells (resting, coef = −0.38[5e–18]; activated, coef=0.29[7e–11]) (Fig. 4E).

Combining single-cell and bulk evidence on CD8^+^ T cell and NK cells, we applied experimental validation of IHC staining (melanoma and control groups, *n* = 4, respectively) to assess the marginal infiltration of CD8^+^ cells (representing CD8 T cells) and CD57^+^ cells (representing NK cells) in melanoma tissues compared to control tissues. As shown in Fig. 4F, samples from melanoma contained fewer CD8^+^ T cells (marked with *CD8*) and NK cells (marked with *CD57*) in the lymphocyte area, in contrast to the control group (Fig. 4G).

### PRGs predominantly expressed in immune cells

**Fig. 5:**
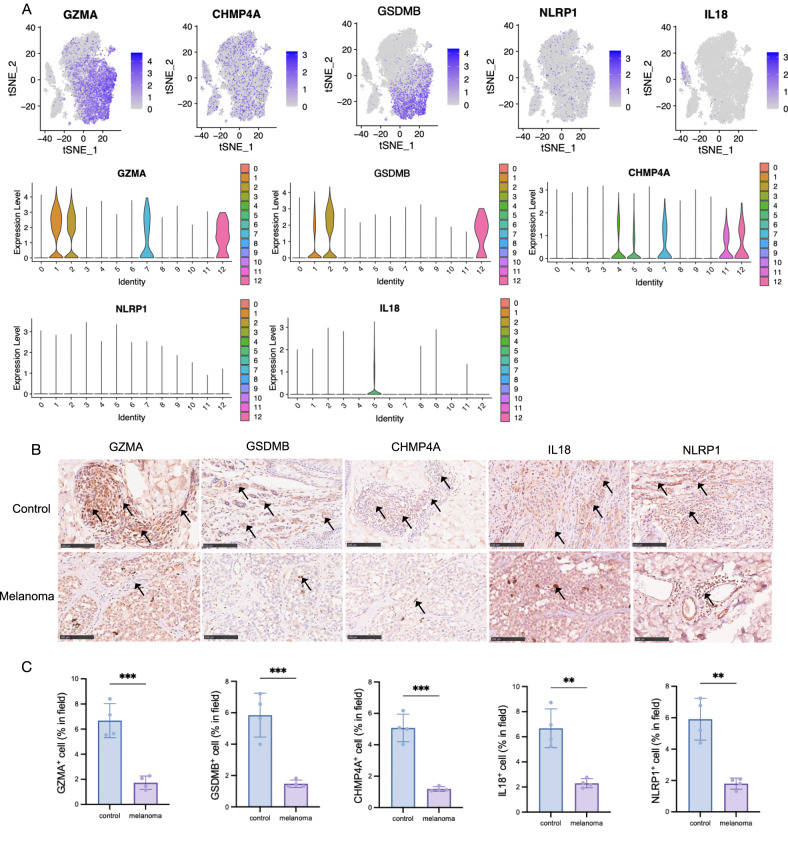
PRGs were primarily expressed in immune cells. **A** Feature plots depicting the expression of key pyroptosis, violin plots were also displayed to determine the cell type. **B** Representative IHC stanning of GZMA^+^, GSMDB^+^, CHMP4A^+^, NLRP1^+^, and IL18^+^ in melanoma specimens, especially in lymphocyte areas. Scale bar = 100 μm, *n* = 4 samples per group. **C** The quantification of PRGs protein in dermal areas, *p < 0.05, **p < 0.01, ***p < 0.001, ****p < 0.0001, compared with the control group.

Our study on the cellular origins of the secreted PRGs demonstrated that the five PRGs were primarily expressed in immune cells, specifically in subgroups related to T cells (Fig. 3F). Notably, T cell subgroups (cluster 2, 3, 24) exhibited the presence of *GZMA* and *GSDMB*. After isolating the immune cells, it was observed that PRGs are predominantly expressed in CD8 ^+^ T cells (cluster 1,7) and NK cells (cluster 2) (Fig. 5A). Our study provided evidence on *GSDMB* that it was relevant to better OS of melanoma and highly expressed in NK cells (cluster 2), followed by CD8^+^ T cells (cluster 1). There’s no differential expression of *CHMP4A* among immune subgroups, while less expression of NLRP1 was observed. Moreover, our results provide evidence that *IL18* could be considered a critical biomarker in myeloid cells, which was further considered a protective predictor in melanoma. In agreement, the direct correlation across immune cells and pyroptosis hub genes of transcriptome sequencing verified the correlation of CD8 ^+^ T cells and *GZMA* (*p* = 8.84e-203[0.91, 0.94]), as well as NK cells and *GSDMB* (*p* = 2.69e-20[0.33, 0.48]) (Table 2).

**Table 2:** Pyroptosis and immune cell analysis by single cell and bulk seq.

PRGs	Single cell			Bulk seq					
				WGCNA		Immune infiltration		Prognosis	
	Cell type	log2FC	*P* value	Module	Cell type	Cell type	*P* value	HR	*P* value
*GZMA*	CD8^+^ T cell	1.34	<0.0001	Blue	CD8^+^ T cell: 0.44 (2e–13) NK cell resting: -0.38 (5e–18) NK cell activated: 0.29 (7e–11) M1 Macrophage: 0.39 (1e–18)	CD8^+^ T cell	8.84e-203	0.526 (0.4–0.692)	<0.0001
	NK cell	0.91	<0.0001						
*GSDMB*	NK cell	2.24	<0.0001			NK cell	2.69e-20	0.749 (0.572–0.98)	0.035
*NLRP1*	/	/	/			CD8^+^ T cell	2.64e-36	0.605 (0.462–0.793)	<0.0001
*IL18*	/	/	/			Macrophage	1.05e-103	0.517 (0.392–0.68)	<0.0001
*CHMP4A*	T cell	0.58	<0.0001			Neutrophil	8.52e-09	0.595 (0.454–0.78)	<0.0001

In terms of experiment validation, providing insights into the level of immune infiltration and activation. The contribution of IL18^+^ cells and NLRP1^+^ cells was decreased in stromal areas in melanoma compared with control samples by IHC experiment (melanoma and control groups, *n* = 4, respectively). Notably, our observations revealed that the pyroptosis-expressed cells, particularly GZMA^+^ cells, GSDMB^+^ cells, and CHMP4A^+^ cells were encompassed by lymphocyte infiltrate, as depicted by the direction of arrows in Fig. 5B. The above experimental evidence revealed that PRGs may mainly express in immune cells (Fig. 5C), nevertheless, it should be further confirmed by immunofluorescence colocalization analysis.

### GZMA and GSDMB are constitutively secreted by CD8^+^ T cells and NK cells

**Fig. 6:**
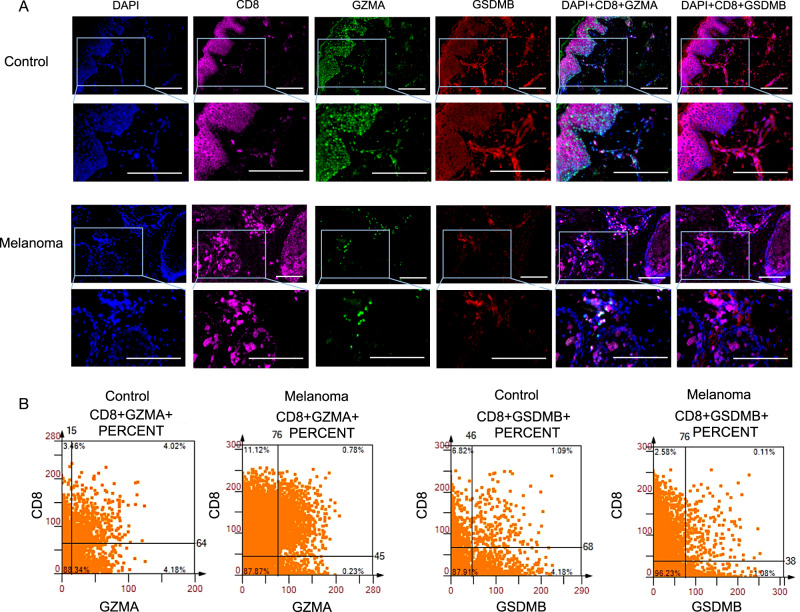
GZMA^+^ cells and GSDMB^+^ cells are secreted by CD8^+^ T cells. **A** Representative multi-color staining of phenotypes of control and melanoma. 40,6-diamidino-2phenylindole (DAPI) (blue), CD8 (pink), GZMA (green), and GSDMB (red). Scale bars, 50 μm. **B** The scattergrams of different CD8^+^GZMA^+^and CD8^ +^ GSDMB^+^ percent cells among the whole sample.

The cytometry panoramic tissue quantification assay developed by Li et al. [41], known as TissueFAXS, was utilized to elucidate the distinct spatial roles of CD8^+^ T cells and NK cells. Figure 6A displays illustrative images demonstrating the presence of GZMA^+^ and GSDMB^+^ in tumor cells, accompanied by the infiltration of CD8^+^ T cells into the immune cell region. The co-expression of CD8 and GZMA was prominently observed in control specimens at a rate of 4.02%, whereas the expression of GZMA in CD8^+^ T cells in melanoma specimens was minimal, measuring only 0.11%. The co-expression of GSDMB^+^ cells in tumor CD8^+^ cells exhibited a consistently lower frequency (0.78%) compared to control samples (1.09%), as shown in Fig. 6B. In combination, tumor CD8 ^+^ T cell downregulated pyroptosis expression percentage. The coexistence of reduced expression percentages of GZMA^+^ cells in tumor CD8^+^ T cells also suggests a diminished immune checkpoint and cellular cytotoxicity in melanoma.

**Fig. 7:**
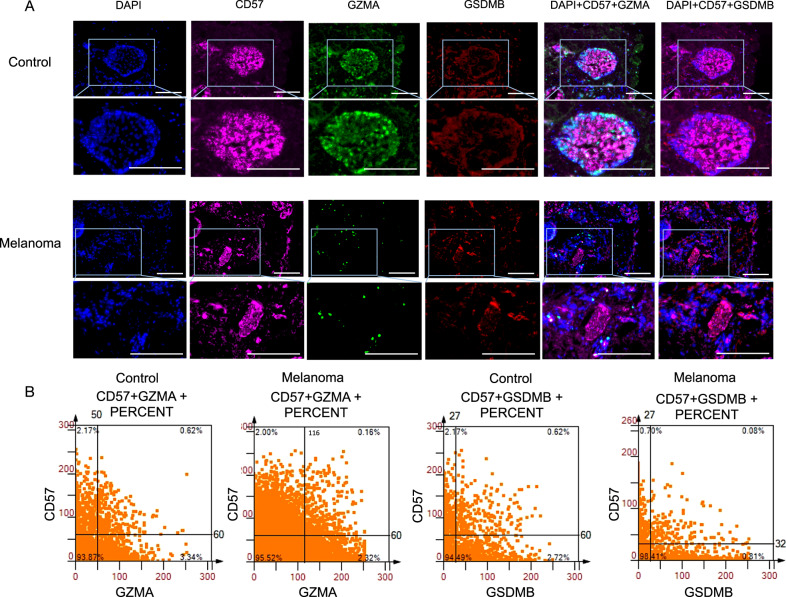
GZMA^+^ cells and GSDMB^+^ cells are secreted by NK cells. **A** Representative multi-color staining of phenotypes of control and melanoma. 40,6-diamidino-2phenylindole (DAPI) DAPI (blue), CD57 (pink), GZMA (green), and GSDMB (red). Scale bars, 50 μm. **B** The scattergrams of different CD57^+^GZMA^+^ and CD57^+^GSDMB^+^ percent cells among the whole sample.

Moreover, within the context of multiplex immunofluorescence staining experiments, the concurrent expression of GZMA and GSDMB has been detected in CD57^+^ cells, indicating a potential correlation with NK cells (Fig. 7A). In control specimens, the co-expression of CD57 and GZMA was observed prominently, with a frequency of 0.62% (Fig. 7B). Conversely, the expression of GZMA in CD57^+^ cells within melanoma specimens was minimal, measuring merely 0.15%. A notable decrease was obtained that tumor CD57^+^ cells barely expressed GSDMB (0.08%), as compared to the control groups (0.62%). The findings of this offer substantiation and validation to the assertion made by scRNA-seq that GZMA^+^ cells and GSDMB^+^ cells are secreted by CD8 ^+^ T cells and NK cells. GZMA and GSDMB could be regarded as significant indicators of immune therapeutic strategies of melanoma.

### Clinical relevance and pyroptosis prognostic nomogram

**Fig. 8:**
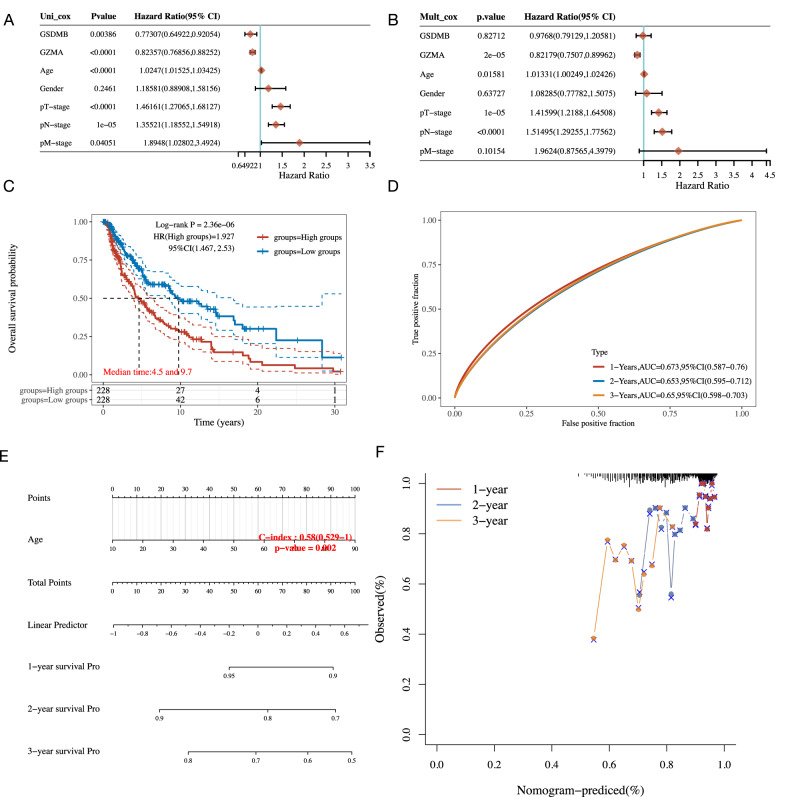
Clinical relevance and individualized prognostic nomogram. **A** Forest plot of univariate Cox regression of the PRGs, including pyroptosis (GZMA and GSDMB) and clinical predicts (age, gender, pT/N/M-stage). **B** Forest plot of multivariate Cox regression of the PRGs, including pyroptosis (GZMA and GSDMB) and clinical predicts (age, gender, pT/N/M-stage). **C** K–M analysis showing the overall survival rate of high-risk patients (red) and low-risk patients (blue). The numbers of patients and the risk classification are indicated in the figure. **D** ROC curve was adopted to evolute the prediction performance of pyroptosis-related signature. **E** The individual prognostic nomogram of the 1-year, 2-year, and 3-year survival prediction of melanoma patients. **F** Calibration curve for the individual prognostic nomogram. A dashed diagonal line represents the ideal nomogram, and the blue line, red line, and orange line represent the 1-y, 2-y, and 3-y observed nomograms.

Finally, we aimed to investigate the clinical relevance on the above five PRGs and conducted an individual prediction tool. GZMA (*P* < 0.0001, HR = 0.82 [0.77, 0.88]) and GSDMB (*P* < 0.01, HR = 0.77 [0.65, 0.92]) were selected as independent protective factors (Fig. 8A) Several clinical predictors were determined as independent risk factors, in which the most hazard predictors included Age (*P* < 0.0001, HR = 1.02 [1.02, 3.34]), T-stage (*P* < 0.0001, HR = 1.46 [1.27, 1.68]), M-stage (*P* = 0.04, HR = 1.89 [1.02, 3.49]) (Fig. 8A, B). The most significant was obtained from N-stage (*P* = 1e-05, HR = 1.35 [1.18, 1.55]). The prognostic K-M curves of pyroptosis indicated a higher mortality rate and higher risk score for patients with low PRGs expression (Fig. 8C, D).

Intending to optimize prognostic risk prediction in individual patients, we constructed a prognostic nomogram featuring PRGs and clinical features based on the results of the single-cell sequencing analysis and Cox proportional hazards analysis. In the nomogram (Fig. 8E), the linear predictor was calculated to predict the 1/2/3-year survival rates of melanoma patients. The model prognostic discrimination performance of the individual nomogram (C-index=0.58[0.529, 1], *P* = 0.002) was significantly higher than the American Joint Committee on Cancer (AJCC) TNM system, which is widely adopted in clinical practice [42]. Besides, model validation was also performed, and the results of the calibration plot of the 1-year, 2-year, and 3-year OS revealed good calibration by showing comparisons between estimated and actual observations (Fig. 8F).

## Discussion

Histologically, melanoma tissues had fewer positive cells percentage of PRGs, *GZMA, GSDMB, NLRP1, IL18,* and *CHMP4A* in epidermal than in normal skin. Pyroptosis, a new frontier in cancer, affects the tumor microenvironment and tumor immunotherapy [43]. Nevertheless, the role of pyroptosis remains controversial, which reason is partly due to the heterogeneity of the cellular composition in melanoma. Traditionally, the prior studies solely selected the mRNA levels of the protein-coding PRGs [21, 24, 25]. However, the above evidence only on sample-based expression was not enough to determine the cell lines of pyroptosis and assess salient cellular heterogeneity. It is unknown from which cells pyroptosis-related macromolecules originate. Toward cellular heterogeneity, as tumor-associated cell types are increasingly studied using scRNA-seq, the abundance and functional state of these cells are being characterized and have provided unprecedented detail of cellular composition heterogeneity [29]. Consequently, we have presented an extensive single-cell transcriptome analysis elucidating the pyroptosis phenomenon in skin melanoma, thereby facilitating a comprehensive comprehension of the tumor microenvironment and cellular constituents associated with pyroptosis.

The single-cell data presented in this study provides insight into a cell–cell interaction network implicated in the pathogenesis of melanoma, with a focus on the dysregulation of immune cells and the notable distribution of pyroptosis in the immune cell region, particularly T cells, B cells, and myeloid cells. Based on the aforementioned evidence, we partitioned the 18,295 immune cells and identified CD8^+^ T cells as primitive immune clusters, and consistently, we observed dysregulation in marginal infiltration of CD8^+^ cells (representing CD8^+^ T cells). In agreement with our results, Li et al. have made a significant finding in their research, revealing the existence of a substantial cohort of CD8^ +^ T cells that undergo a continuous transition from an initial effector “transitional” state to a dysfunctional T cell state. It is noteworthy that CD8 ^+^ T cells expressing a comprehensive cytotoxic gene repertoire are infrequently observed [44]. Sade-Feldman et al. demonstrated that CD8^+^ T cells are associated with either tumor regression or progression in patients [45].

Additionally, NK cells have been identified as an imbalanced subgroup, specifically designated as cluster 2 within the immune cell clusters feature map. As shown in the IHC staining, samples from melanoma contained fewer and NK cells (marked with *CD57*) in the lymphocyte area, in contrast to melanotic naevus samples in the control group. and CD57^+^ cells (representing NK cells). According to the report, NK cells that underwent differentiation increased cytotoxicity towards melanoma targets by exhibited heightened production of IFNγ [44]. Barry and colleges find that NK cell frequency correlates with patient responsiveness to anti-PD-1 immunotherapy, as well as an association with improved overall survival [46]. These findings have the potential to predict positive clinical outcomes in a separate group of patients undergoing checkpoint therapy.

Notably, lymphocyte infiltrates were observed to encompass cells expressing pyroptosis, particularly in GZMA^+^ cells, GSDMB^+^ cells, and CHMP4A^+^ cells. Multiplex immunofluorescence staining results furtherer confirmed GZMA^+^ cells and GSDMB^+^ cells are secreted by CD8 ^+^ T cells and NK cells, suggesting ruduced tumor immune cells leading to lower pyroptosis capability on anti-melanoma properties. These results corroborate the ideas of Wang et al, who suggested that cell pyroptosis activated the T cell-mediated anti-tumor immune response, thereby regulating the tumor immune microenvironment, effectively [47]. It has been reported that the *GZMB* of the granzyme from killer cells can directly cut *GSDME* and activate cell pyrosis, and the occurrence of cell pyroptosis further activates the anti-tumor immune response and inhibits tumor growth [48].

Rapid decreases in *GZMA* in melanoma were observed that GZMA^+^ merge CD8^+^ T cell in melanoma specimens was minimal (0.11%) compared to control specimens (4.02%). The presence of decreased expression levels of GZMA^+^ cells within tumor CD8^+^ T cells further implies a weakened immune checkpoint and diminished cellular cytotoxicity in melanoma. In the process of cytotoxic lymphocyte killing target cells, cytotoxic lymphocyte-derived *GZMA* cleaves pore activity, thereby triggering target cell pyroptosis, a molecular mechanism that enhances anti-tumor immunity [49]. What stands out in this table is that *GZMA* almost clustered together in melanoma samples, especially in CD8^ +^ T cells and NK cells. The correlation between *GZMA* expression and CD8 ^+^ T cells matches those observed in earlier studies. The *GZMA* was identified as CD8^ +^ T cell co-expression genes that promoted infiltration of CD8 ^+^ T cells in an antigen presentation process of cancer [50]. With *GZMA* expressed by significantly higher percentages of mucosal CD8 ^+^ T cells [51], findings suggest that melanoma harboring high *GZMA* expression may respond preferentially to cancer immunotherapies [52].

A significance decrease was obtained that tumor CD57^+^ cells barely expressed GSDMB (0.08%), as compared to the control groups (0.62%). GSDMB is a member of an extensive family of pore-forming cytolysins, which are responsible for the activation of inflammatory cell death pathways [53]. Compare the result of *GSDMB* with that found by Zhou et al., (2020) who found that NK cells and cytotoxic T lymphocytes kill proinflammatory cells through pyroptosis driven by the *GSDMB*-mediated cytotoxic lymphocyte-killing mechanism, which may enhance anti-tumor immunity [49]. Our study provided evidence on *GSDMB* that it was relevant to better OS of melanoma and highly expressed in NK cells. This finding is also consistent with previous research that *GSDMB* could execute inflammatory cell death programs [54] by NK cells [53]. Besides, several recent studies found that cancer with low *NLRP1* expression had low immune cell infiltration [55] and a poorer prognosis [56], which is consistent with our results. Contrary to expectations, it’s illustrated that *IL18* is a critical pro-inflammatory factor in cell swelling and intracellular inflammasomes release [57, 58]. Very little was found in the literature on the question of *CHMP4A* in melanoma, which needs further research.

In summary, pyroptosis-related biomarkers were found to be differentially expressed in different immune cells. A prognostic model was developed based on PRGs expression and clinical characteristics. However, details of the exact mechanisms need to be evaluated in future studies. Moreover, research is still needed to develop practical strategies to translate melanoma PRGs into clinical applications.

## Conclusion

The single-cell expression profiles of PRGs were evaluated in our study, dysregulation in the expression of pyroptosis-related genes, particular in immune cells. Tumor CD8^+^ cells (representing CD8^+^ T cells) and CD57^+^ cells (representing NK cells) downregulated pyroptosis expression percentage, especially GZMA^+^ and GSDMB^+^, acting as a protective prognostic predictor clinically. The primary objective of this study is to enhance comprehension of the mechanisms linked to pyroptosis, thereby aiding in the identification and development of more efficacious therapeutic targets and biomarkers for immunotherapies in individuals with melanoma.

### Supplementary information

Table S1

Original Data File

## Data Availability

The datasets download link for this study can be found in the additional file for review purposes only.

## Code availability

The authors of this article will provide the R code supporting the conclusions upon request via email, without any unwarranted hesitation, to any researcher who meets the necessary qualifications.

## References

[1] Hseu YC, Chiang YC, Vudhya Gowrisankar Y, Lin KY, Huang ST, Shrestha S (2020). The in vitro and in vivo anticancer properties of chalcone flavokawain B through induction of ROS-mediated apoptotic and autophagic cell death in human melanoma cells. Cancers.

[2] Dudek-Perić AM, Ferreira GB, Muchowicz A, Wouters J, Prada N, Martin S (2015). Antitumor immunity triggered by melphalan is potentiated by melanoma cell surface–associated calreticulin. Cancer Res.

[3] Twyman-Saint Victor C, Rech AJ, Maity A, Rengan R, Pauken KE, Stelekati E (2015). Radiation and dual checkpoint blockade activate non-redundant immune mechanisms in cancer. Nature.

[4] Spranger S, Bao R, Gajewski TF (2015). Melanoma-intrinsic β-catenin signalling prevents anti-tumour immunity. Nature.

[5] Li C, Kuai L, Cui R, Miao X (2022). Melanogenesis and the targeted therapy of melanoma. Biomolecules.

[6] Zhu L, Kalimuthu S, Gangadaran P, Oh JM, Lee HW, Baek SH (2017). Exosomes derived from natural killer cells exert therapeutic effect in melanoma. Theranostics.

[7] Goff SL, Dudley ME, Citrin DE, Somerville RP, Wunderlich JR, Danforth DN (2016). Randomized, prospective evaluation comparing intensity of lymphodepletion before adoptive transfer of tumor-infiltrating lymphocytes for patients with metastatic melanoma. J Clin Oncol.

[8] Miller KD, Nogueira L, Mariotto AB, Rowland JH, Yabroff KR, Alfano CM (2019). Cancer treatment and survivorship statistics, 2019. CA Cancer J Clin.

[9] Vishwakarma M, Piddini E (2020). Outcompeting cancer. Nat Rev Cancer.

[10] Xia X, Wang X, Cheng Z, Qin W, Lei L, Jiang J (2019). The role of pyroptosis in cancer: pro-cancer or pro-“host”?. Cell Death Dis.

[11] Hsu SK, Li CY, Lin IL, Syue WJ, Chen YF, Cheng KC (2021). Inflammation-related pyroptosis, a novel programmed cell death pathway, and its crosstalk with immune therapy in cancer treatment. Theranostics.

[12] Yang Y, Liu PY, Bao W, Chen SJ, Wu FS, Zhu PY (2020). Hydrogen inhibits endometrial cancer growth via a ROS/NLRP3/caspase-1/GSDMD-mediated pyroptotic pathway. BMC Cancer.

[13] Zhang J, Jiang N, Zhang L, Meng C, Zhao J, Wu J (2020). NLRP6 expressed in astrocytes aggravates neurons injury after OGD/R through activating the inflammasome and inducing pyroptosis. Int Immunopharmacol.

[14] He X, Fan X, Bai B, Lu N, Zhang S, Zhang L (2021). Pyroptosis is a critical immune-inflammatory response involved in atherosclerosis. Pharmacol Res.

[15] Lu X, Guo T, Zhang X (2021). Pyroptosis in cancer: friend or foe?. Cancers.

[16] Wang Y, Kong H, Zeng X, Liu W, Wang Z, Yan X (2016). Activation of NLRP3 inflammasome enhances the proliferation and migration of A549 lung cancer cells. Oncol Rep.

[17] Wang H, Luo Q, Feng X, Zhang R, Li J, Chen F (2018). NLRP3 promotes tumor growth and metastasis in human oral squamous cell carcinoma. BMC Cancer.

[18] Hou J, Zhao R, Xia W, Chang CW, You Y, Hsu JM (2020). PD-L1-mediated gasdermin C expression switches apoptosis to pyroptosis in cancer cells and facilitates tumour necrosis. Nat Cell Biol.

[19] Wei Q, Mu K, Li T, Zhang Y, Yang Z, Jia X (2014). Deregulation of the NLRP3 inflammasome in hepatic parenchymal cells during liver cancer progression. Lab Invest.

[20] Dupaul-Chicoine J, Arabzadeh A, Dagenais M, Douglas T, Champagne C, Morizot A (2015). The Nlrp3 inflammasome suppresses colorectal cancer metastatic growth in the liver by promoting natural killer cell tumoricidal activity. Immunity.

[21] Xie J, Li H, Chen L, Cao Y, Hu Y, Zhu Z (2021). A novel pyroptosis-related lncRNA signature for predicting the prognosis of skin cutaneous melanoma. Int J Gen Med.

[22] Meacham CE, Morrison SJ (2013). Tumour heterogeneity and cancer cell plasticity. Nature.

[23] Tirosh I, Izar B, Prakadan SM, Wadsworth MH, Treacy D, Trombetta JJ (2016). Dissecting the multicellular ecosystem of metastatic melanoma by single-cell RNA-seq. Science.

[24] Wu Z, Chen L, Jin C, Xu J, Zhang X, Yao Y (2021). A novel pyroptosis-associated gene signature for immune status and prognosis of cutaneous melanoma. PeerJ.

[25] Niu Z, Xu Y, Li Y, Chen Y, Han Y (2022). Construction and validation of a novel pyroptosis-related signature to predict prognosis in patients with cutaneous melanoma. Math Biosci Eng.

[26] Papalexi E, Satija R (2018). Single-cell RNA sequencing to explore immune cell heterogeneity. Nat Rev Immunol.

[27] Han X, Zhou Z, Fei L, Sun H, Wang R, Chen Y (2020). Construction of a human cell landscape at single-cell level. Nature.

[28] Yan G, Li L, Zhu S, Wu Y, Liu Y, Zhu L (2021). Single-cell transcriptomic analysis reveals the critical molecular pattern of UV-induced cutaneous squamous cell carcinoma. Cell Death Dis.

[29] Kumar MP, Du J, Lagoudas G, Jiao Y, Sawyer A, Drummond DC (2018). Analysis of single-cell RNA-seq identifies cell-cell communication associated with tumor characteristics. Cell Rep.

[30] Shalek AK, Satija R, Adiconis X, Gertner RS, Gaublomme JT, Raychowdhury R (2013). Single-cell transcriptomics reveals bimodality in expression and splicing in immune cells. Nature.

[31] Macosko EZ, Basu A, Satija R, Nemesh J, Shekhar K, Goldman M (2015). Highly parallel genome-wide expression profiling of individual cells using nanoliter droplets. Cell.

[32] Patel AP, Tirosh I, Trombetta JJ, Shalek AK, Gillespie SM, Wakimoto H (2014). Single-cell RNA-seq highlights intratumoral heterogeneity in primary glioblastoma. Science.

[33] Batlevi CL, Sha F, Alperovich A, Ni A, Smith K, Ying Z (2020). Follicular lymphoma in the modern era: survival, treatment outcomes, and identification of high-risk subgroups. Blood Cancer J.

[34] Savina M, Le Cesne A, Blay JY, Ray-Coquard I, Mir O, Toulmonde M (2017). Patterns of care and outcomes of patients with METAstatic soft tissue SARComa in a real-life setting: the METASARC observational study. BMC Med.

[35] Kuai L, Song JK, Zhang RX, Xing M, Luo Y, Ru Y (2020). Uncovering the mechanism of Jueyin granules in the treatment of psoriasis using network pharmacology. J Ethnopharmacol.

[36] Stuart T, Butler A, Hoffman P, Hafemeister C, Papalexi E, Mauck WM (2019). Comprehensive integration of single-cell data. Cell.

[37] Zhang M, Yang H, Wan L, Wang Z, Wang H, Ge C (2020). Single-cell transcriptomic architecture and intercellular crosstalk of human intrahepatic cholangiocarcinoma. J Hepatol.

[38] Nghiem PT, Bhatia S, Lipson EJ, Kudchadkar RR, Miller NJ, Annamalai L (2016). PD-1 blockade with pembrolizumab in advanced merkel-cell carcinoma. N. Engl J Med.

[39] Zhang C, Shen H, Yang T, Li T, Liu X, Wang J (2022). A single-cell analysis reveals tumor heterogeneity and immune environment of acral melanoma. Nat Commun.

[40] Trapnell C, Cacchiarelli D, Grimsby J, Pokharel P, Li S, Morse M (2014). The dynamics and regulators of cell fate decisions are revealed by pseudotemporal ordering of single cells. Nat Biotechnol.

[41] Li H, Chen J, Li Z, Chen M, Ou Z, Mo M, et al. S100A5 attenuates efficiency of anti‐PD‐L1/PD‐1 immunotherapy by inhibiting CD8^+^ T cell‐mediated anti‐cancer immunity in bladder carcinoma. Adv Sci (Weinh). 2023;e2300110. 10.1002/advs.202300110. 10.1002/advs.202300110 PMC10477882 37414584

[42] Liang W, Zhang L, Jiang G, Wang Q, Liu L, Liu D (2015). Development and validation of a nomogram for predicting survival in patients with resected non-small-cell lung cancer. J Clin Oncol.

[43] Fang Y, Tian S, Pan Y, Li W, Wang Q, Tang Y (2020). Pyroptosis: a new frontier in cancer. Biomed Pharmacother.

[44] Li H, Van Der Leun AM, Yofe I, Lubling Y, Gelbard-Solodkin D, Van Akkooi ACJ (2019). Dysfunctional CD8 T cells form a proliferative, dynamically regulated compartment within human melanoma. Cell.

[45] De Andrade LF, Lu Y, Luoma A, Ito Y, Pan D, Pyrdol JW (2019). Discovery of specialized NK cell populations infiltrating human melanoma metastases. JCI Insight.

[46] Barry KC, Hsu J, Broz ML, Cueto FJ, Binnewies M, Combes AJ (2018). A natural killer–dendritic cell axis defines checkpoint therapy–responsive tumor microenvironments. Nat Med.

[47] Wang Q, Wang Y, Ding J, Wang C, Zhou X, Gao W (2020). A bioorthogonal system reveals antitumour immune function of pyroptosis. Nature.

[48] Zhang Z, Zhang Y, Xia S, Kong Q, Li S, Liu X (2020). Gasdermin E suppresses tumour growth by activating anti-tumour immunity. Nature.

[49] Zhou Z, He H, Wang K, Shi X, Wang Y, Su Y (2020). Granzyme A from cytotoxic lymphocytes cleaves GSDMB to trigger pyroptosis in target cells. Science.

[50] Pan Q, Cheng Y, Cheng D (2021). Identification of CD8+ T cell-related genes: correlations with immune phenotypes and outcomes of liver cancer. J Immunol Res.

[51] Kiniry BE, Hunt PW, Hecht FM, Somsouk M, Deeks SG, Shacklett BL (2018). Differential expression of CD8+ T cell cytotoxic effector molecules in blood and gastrointestinal mucosa in HIV-1 infection. J Immunol.

[52] Inoue H, Park JH, Kiyotani K, Zewde M, Miyashita A, Jinnin M (2016). Intratumoral expression levels of PD-L1, GZMA, and HLA-A along with oligoclonal T cell expansion associate with response to nivolumab in metastatic melanoma. Oncoimmunology.

[53] Hansen JM, de Jong MF, Wu Q, Zhang LS, Heisler DB, Alto LT (2021). Pathogenic ubiquitination of GSDMB inhibits NK cell bactericidal functions. Cell.

[54] Rana N, Privitera G, Kondolf HC, Bulek K, Lechuga S, De Salvo C (2022). GSDMB is increased in IBD and regulates epithelial restitution/repair independent of pyroptosis. Cell.

[55] Shen E, Han Y, Cai C, Liu P, Chen Y, Gao L (2021). Low expression of NLRP1 is associated with a poor prognosis and immune infiltration in lung adenocarcinoma patients. Aging (Albany NY).

[56] Liu LP, Lu L, Zhao QQ, Kou QJ, Jiang ZZ, Gui R (2021). Identification and validation of the pyroptosis-related molecular subtypes of lung adenocarcinoma by bioinformatics and machine learning. Front Cell Dev Biol.

[57] Al Mamun A, Wu Y, Monalisa I, Jia C, Zhou K, Munir F (2020). Role of pyroptosis in spinal cord injury and its therapeutic implications. J Adv Res.

[58] Burdette BE, Esparza AN, Zhu H, Wang S (2021). Gasdermin D in pyroptosis. Acta Pharm Sin B.

