# Correction: Clinical-mediated discovery of pyroptosis in CD8^+^ T cell and NK cell reveals melanoma heterogeneity by single-cell and bulk sequence

**DOI:** 10.1038/s41419-023-06366-y

**Published:** 2024-02-22

**Authors:** Ying Zhang, Yun Bai, Xiao-Xuan Ma, Jian-Kun Song, Yue Luo, Xiao-Ya Fei, Yi Ru, Ying Luo, Jing-Si Jiang, Zhan Zhang, Dan Yang, Ting-Ting Xue, Hui-Ping Zhang, Tai-Yi Liu, Yan-Wei Xiang, Le Kuai, Ye-Qiang Liu, Bin Li

**Affiliations:** 1 Department of Dermatology, Yueyang Hospital of Integrated Traditional Chinese and Western Medicine, Shanghai University of Traditional Chinese Medicine, Shanghai, 200437 China; 2 Shanghai Skin Disease Hospital, Tongji University, Shanghai, 200443 China; 3 Institute of Dermatology, Shanghai Academy of Traditional Chinese Medicine, Shanghai, 201203 China; 4 Shanghai Applied Protein Technology Co., Ltd., 58 Yuanmei Road, Shanghai, 200233 China; 5 School of Rehabilitation Science, Shanghai University of Traditional Chinese Medicine, Shanghai, 201203 China

**Keywords:** Prognostic markers, Melanoma

Correction to: *Cell Death and Disease* 10.1038/s41419-023-06068-5, published online 24 August 2023

In this article, the authors have been affiliated wrongly.

Ying Zhang^1,2,5^, Yun Bai^3,5^, Xiao-Xuan Ma^1,2,5^, Jian-Kun Song^3^, Yue Luo^3^, Xiao-Ya Fei^3^, Yi Ru^1,2^, Ying Luo^1,2^, Jing-Si Jiang^3^, Zhan Zhang^1,2^, Dan Yang^3^, Ting-Ting Xue^1,2^, Hui-Ping Zhang^4^, Tai-Yi Liu^4^, Yan-Wei Xiang^1^, Le Kuai^1,2,*^, Ye-Qiang Liu^3,**^ and Bin Li^2,3,***^

It should read:

Ying Zhang^1,3,6^, Yun Bai^2,6^, Xiao-Xuan Ma^1,3,6^, Jian-Kun Song^2^, Yue Luo^2^, Xiao-Ya Fei^2^, Yi Ru^1,3^, Ying Luo^1,3^, Jing-Si Jiang^2^, Zhan Zhang^1,3^, Dan Yang^2^, Ting-Ting Xue^1,3^, Hui-Ping Zhang^4^, Tai-Yi Liu^4^, Yan-Wei Xiang^1,5^, Le Kuai^1,3,*^, Ye-Qiang Liu^2,**^, Bin Li^2,3,***^

^1^Department of Dermatology, Yueyang Hospital of Integrated Traditional Chinese and Western Medicine, Shanghai University of Traditional Chinese Medicine, Shanghai, 200437, China

^2^Shanghai Skin Disease Hospital, Tongji University, Shanghai, 200443, China

^3^Institute of Dermatology, Shanghai Academy of Traditional Chinese Medicine, Shanghai, 201203, China

^4^Shanghai Applied Protein Technology Co., Ltd., 58 Yuanmei Road, Shanghai 200233, China.

^5^School of Rehabilitation Science, Shanghai University of Traditional Chinese Medicine, Shanghai, 201203, China

^6^These authors contributed equally: Ying Zhang, Yun Bai, Xiao-Xuan Ma.

In order to maintain consistency with the main text, the legend of Fig. 7 “GZMA^+^ cells and GSDMB^+^ cells are secreted by NK T cells” should be corrected as “GZMA^+^ cells and GSDMB^+^ cells are secreted by NK cells”. In accordance with the figure and its context, the legend of Fig. 7B “The scattergrams of different CD8^+^GZMA^+^, CD8^+^GSDMB^+^, CD57^+^GZMA^+^, and CD57^+^GSDMB^+^ percent cells among the whole sample” should be be modified as “The scattergrams of different CD57^+^GZMA^+^ and CD57^+^GSDMB^+^ percent cells among the whole sample”.

**Fig. 7:**
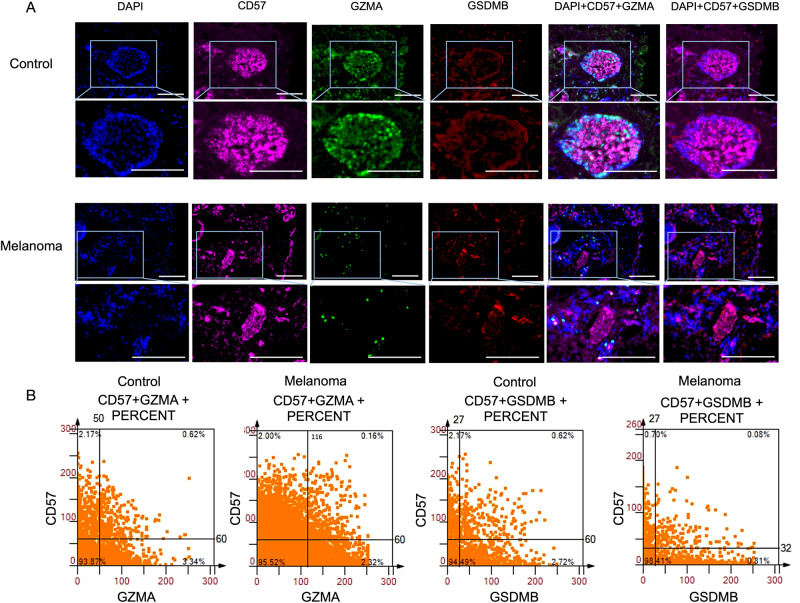
.

Following the citation of Fig. 7B on page 10, “Conversely, the expression of GZMA in CD57^+^ T cells within melanoma specimens was minimal” would better be changed to “Conversely, the expression of GZMA in CD57^+^ cells within melanoma specimens was minimal”.

There was a slip of the pen in the first sentence of Discussion section that “Histologically, melanoma tissues had fewer positive cells percentage of PRGs, GZMA, GSDMB, NLRP1, IL18, and NLRP1 in epidermal than in normal skin” should be changed into “Histologically, melanoma tissues had fewer positive cells percentage of PRGs, GZMA, GSDMB, NLRP1, IL18, and CHMP4A in epidermal than in normal skin”.

The original article has been corrected.

