# Supplementary material for: Clinical-mediated discovery of pyroptosis in CD8+ T cell and NK cell reveals melanoma heterogeneity by single-cell and bulk sequence
Source: Cell Death Dis. 2023 Aug 24;14(8):553. doi: 10.1038/s41419-023-06068-5 (PMC10449777; doi:10.1038/s41419-023-06068-5)
Supplement: Supplementary file 2 — Original Data File [file 41419_2023_6068_MOESM2_ESM.docx]

GraphPad datasheet

| Elisa | Normal | | | | | Melanoma | | | | |
| --- | --- | --- | --- | --- | --- | --- | --- | --- | --- | --- |
| CHMP4A | 350.43 | 330.25 | 372.34 | 355.42 | 343.65 | 289.32 | 230.67 | 192.32 | 222.54 | 246.54 |
| IL18 | 403.23 | 409.13 | 412.22 | 394.65 | 356.54 | 338.12 | 310.24 | 376.42 | 324.43 | 321.65 |
| GSDMB | 450.43 | 430.25 | 440.34 | 400.42 | 398.64 | 189.32 | 230.67 | 192.32 | 222.54 | 254.86 |
| NLRP1 | 288.4 | 290.4 | 320.1 | 301.5 | 320.98 | 158.6 | 155.3 | 180.2 | 192.2 | 210.54 |
| GZMA | 220.1 | 260.4 | 285.3 | 333.2 | 345.43 | 130.4 | 144.6 | 158 | 180.2 | 210.54 |

| IHC-1 | Normal | | | | | Melanoma | | | | |
| --- | --- | --- | --- | --- | --- | --- | --- | --- | --- | --- |
| GZMA | 4.5 | 4.945 | 5.327 | 5.42 | 5.58 | 2.377 | 1.302 | 2.092 | 1.908 | 3.51 |
| GSDMB | 7.591 | 8.372 | 7.003 | 7.002 | 7.139 | 3.137 | 3.363 | 3.872 | 4.896 | 2.308 |
| CHMP4A | 11.658 | 9.464 | 11.028 | 6.303 | 8.422 | 3.753 | 3.617 | 2.509 | 3.791 | 4.779 |
| NLRP1 | 7.622 | 10.56 | 7.861 | 8.399 | 9.276 | 5.323 | 4.333 | 3.395 | 4.358 | 3.672 |
| IL18 | 5.82 | 6.389 | 7.49 | 5.203 | 6.768 | 2.023 | 3.023 | 4.749 | 4.782 | 3.698 |

| IHC-2 | Control | | | | Melanoma | | | |
| --- | --- | --- | --- | --- | --- | --- | --- | --- |
| CD8 | 3.909 | 3.289 | 3.425 | 2.491 | 1.675 | 2.322 | 1.923 | 2.911 |
| CD57 | 2.246 | 2.698 | 3.545 | 2.668 | 1.3 | 1.22 | 0.462 | 0.348 |
| GZMA | 5.54 | 5.639 | 8.4 | 7.14 | 2.07 | 1.138 | 2.27 | 1.45 |
| GSDMB | 3.988 | 5.65 | 7.21 | 6.56 | 1.82 | 1.32 | 1.44 | 1.33 |
| CHMP4A | 4.022 | 6.18 | 5.01 | 5.09 | 1.39 | 1.07 | 1.13 | 1.135 |
| IL18 | 6.947 | 5.804 | 8.73 | 5.24 | 2.766 | 2.333 | 1.909 | 2.176 |
| NLRP1 | 4.395 | 5.205 | 7.241 | 6.791 | 1.309 | 2.021 | 2.093 | 1.798 |

IHC uncropped figure-1

21-176_CHMP4A
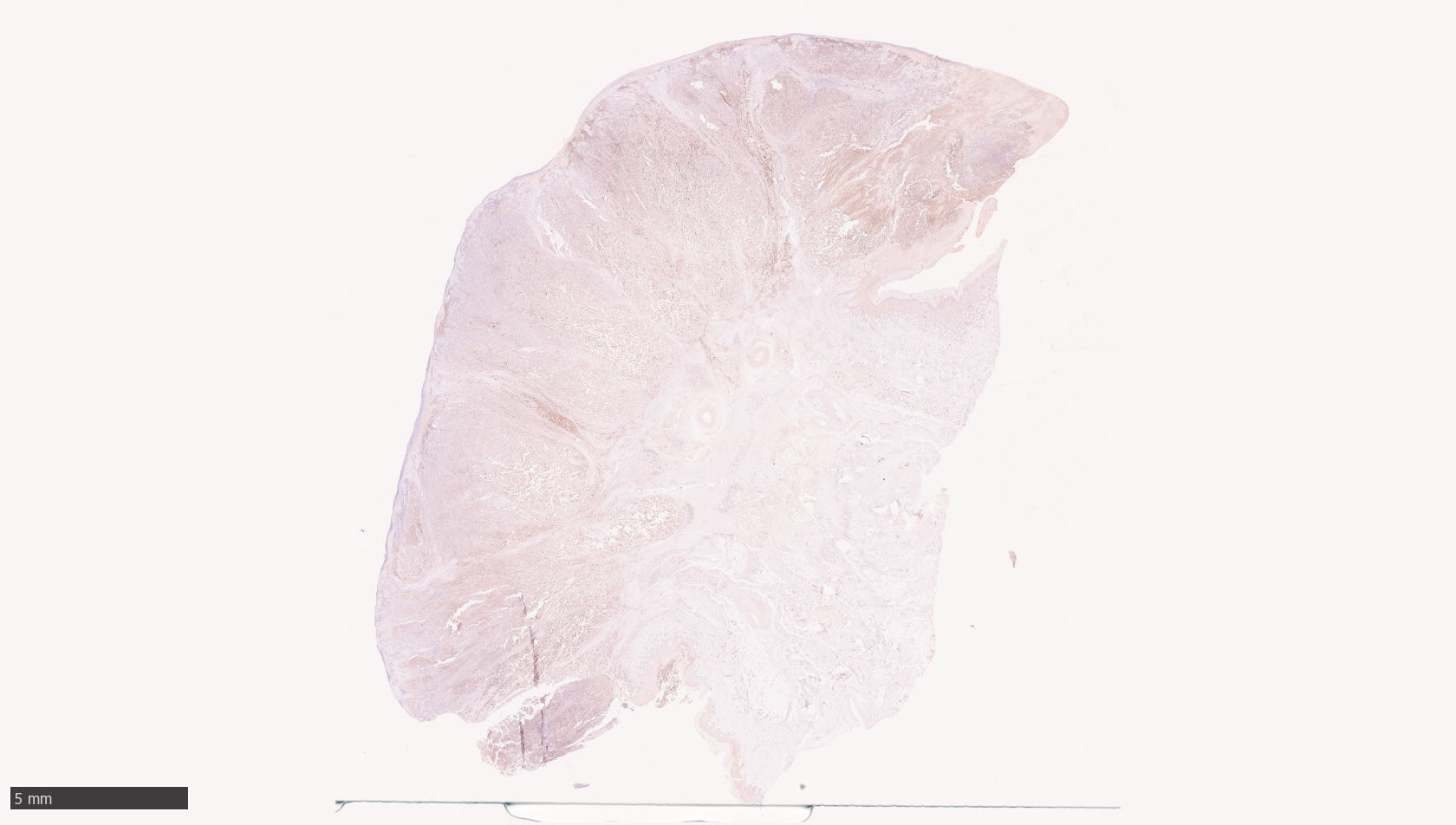
 21-176_GSDMB
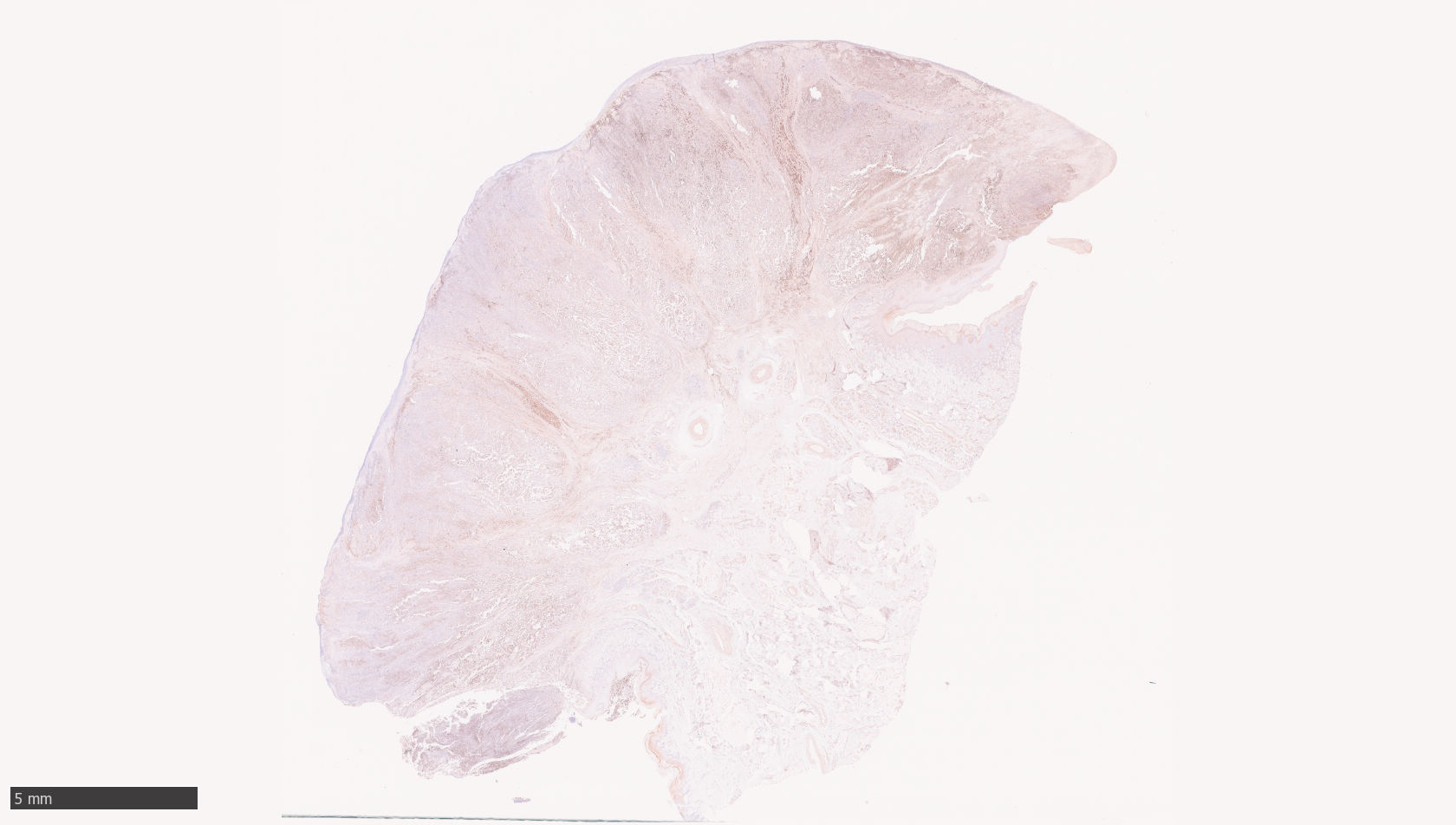


21-176_GZMA
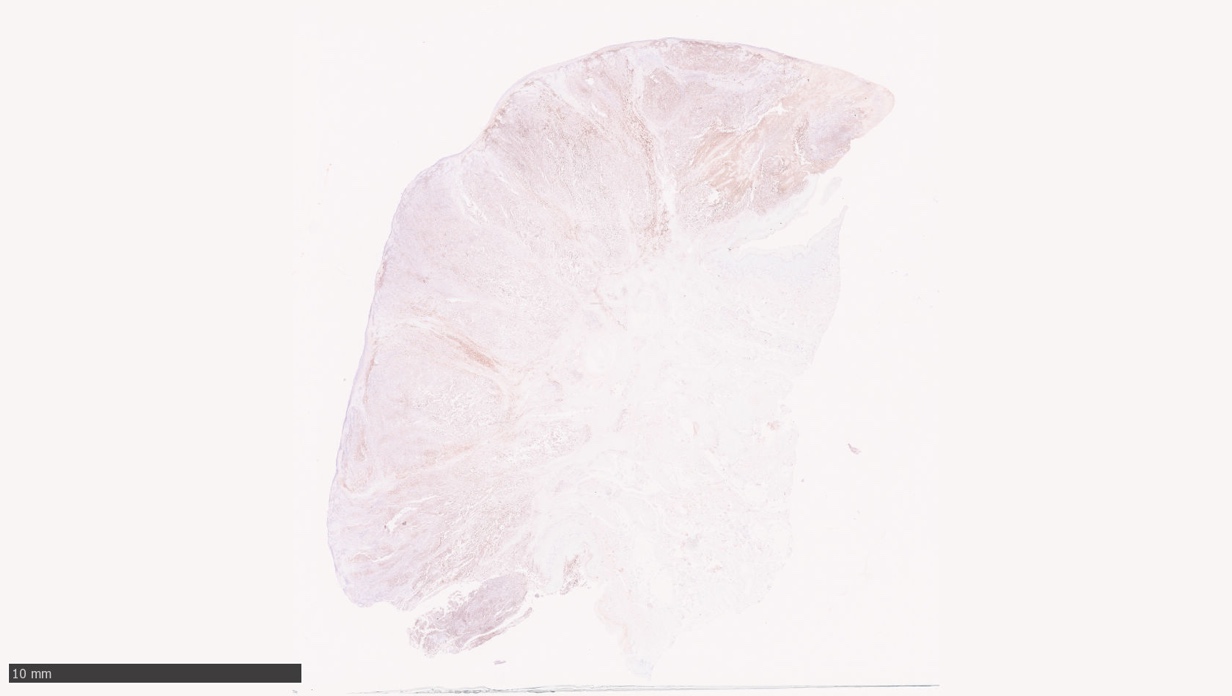
 21-176_IL18
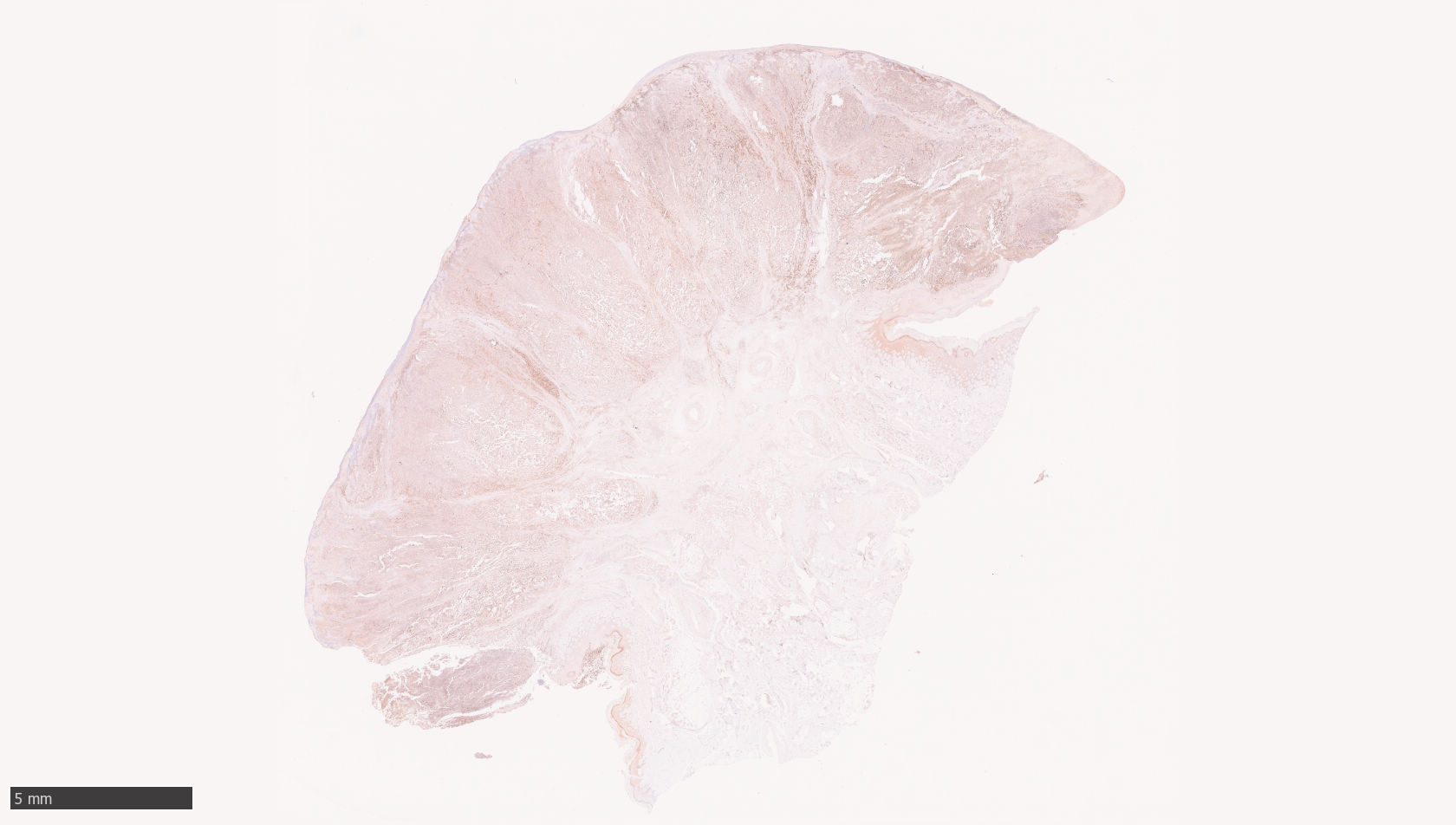


21-176_NLRP1
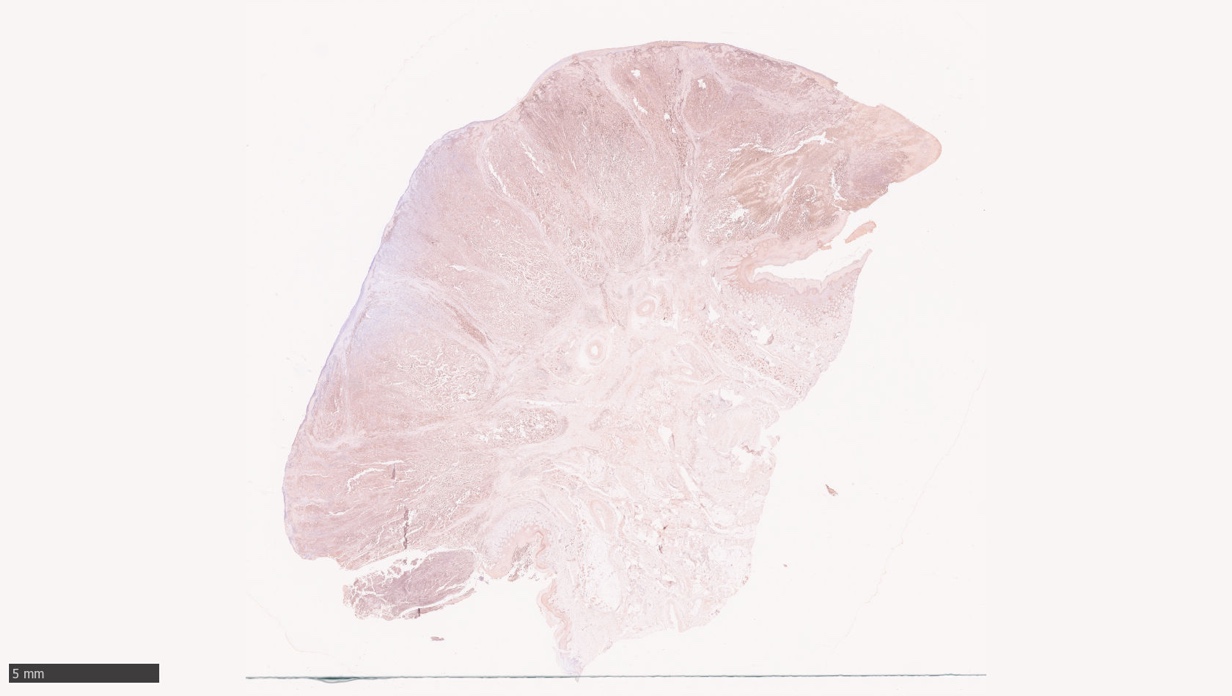


21-7818_CHMP4A
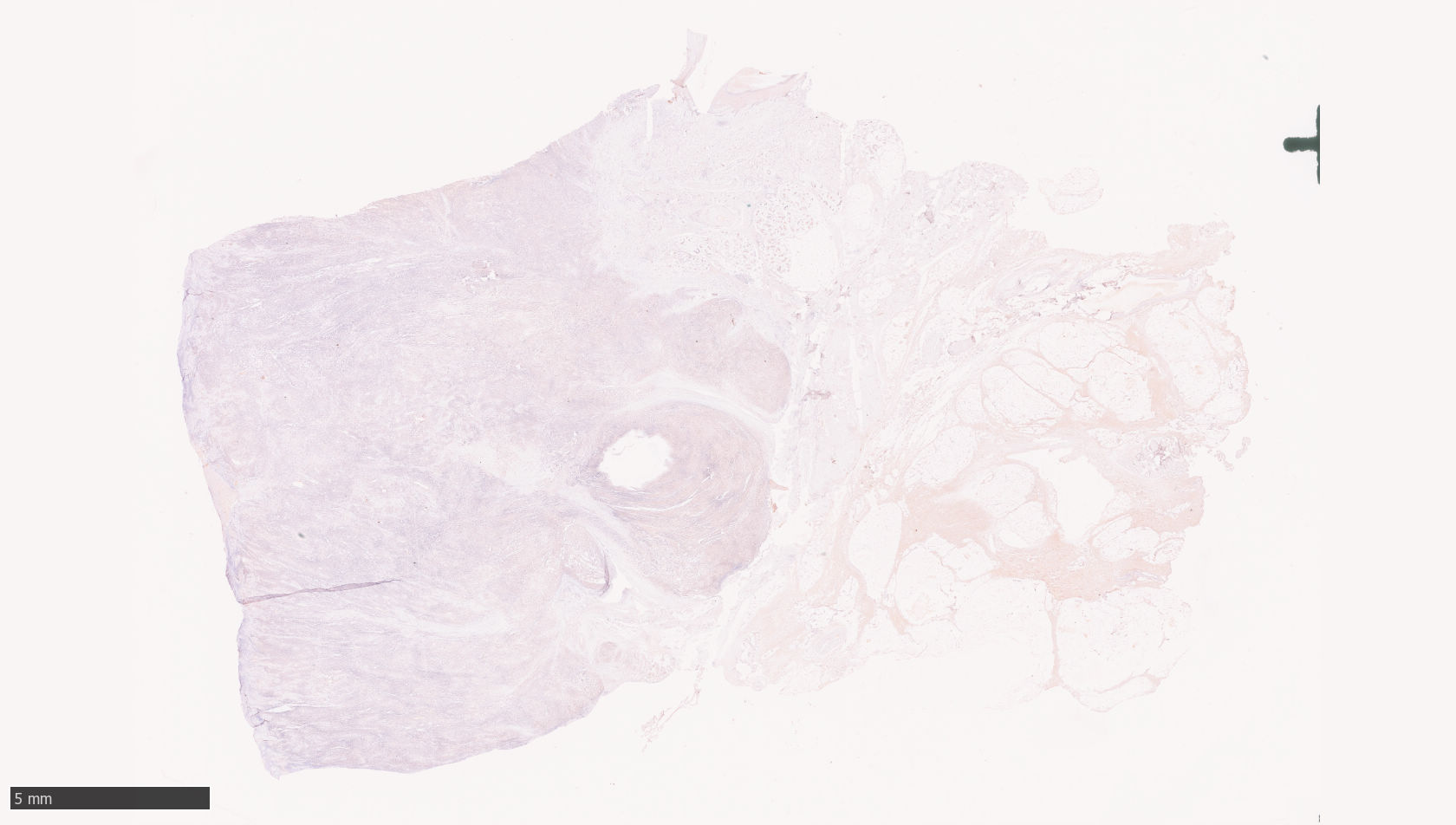


21-7818_GSDMB
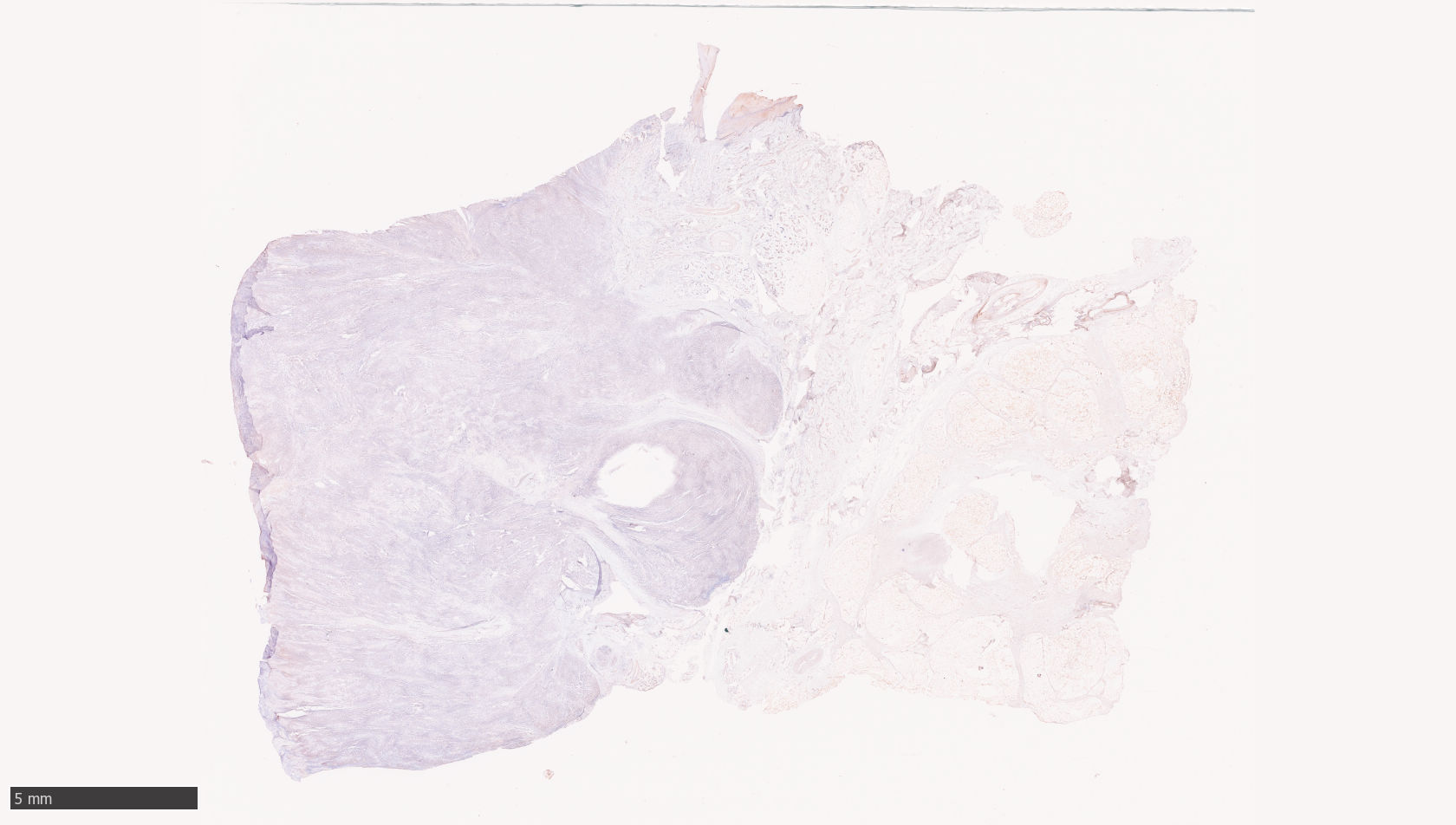


21-7818_GZMA
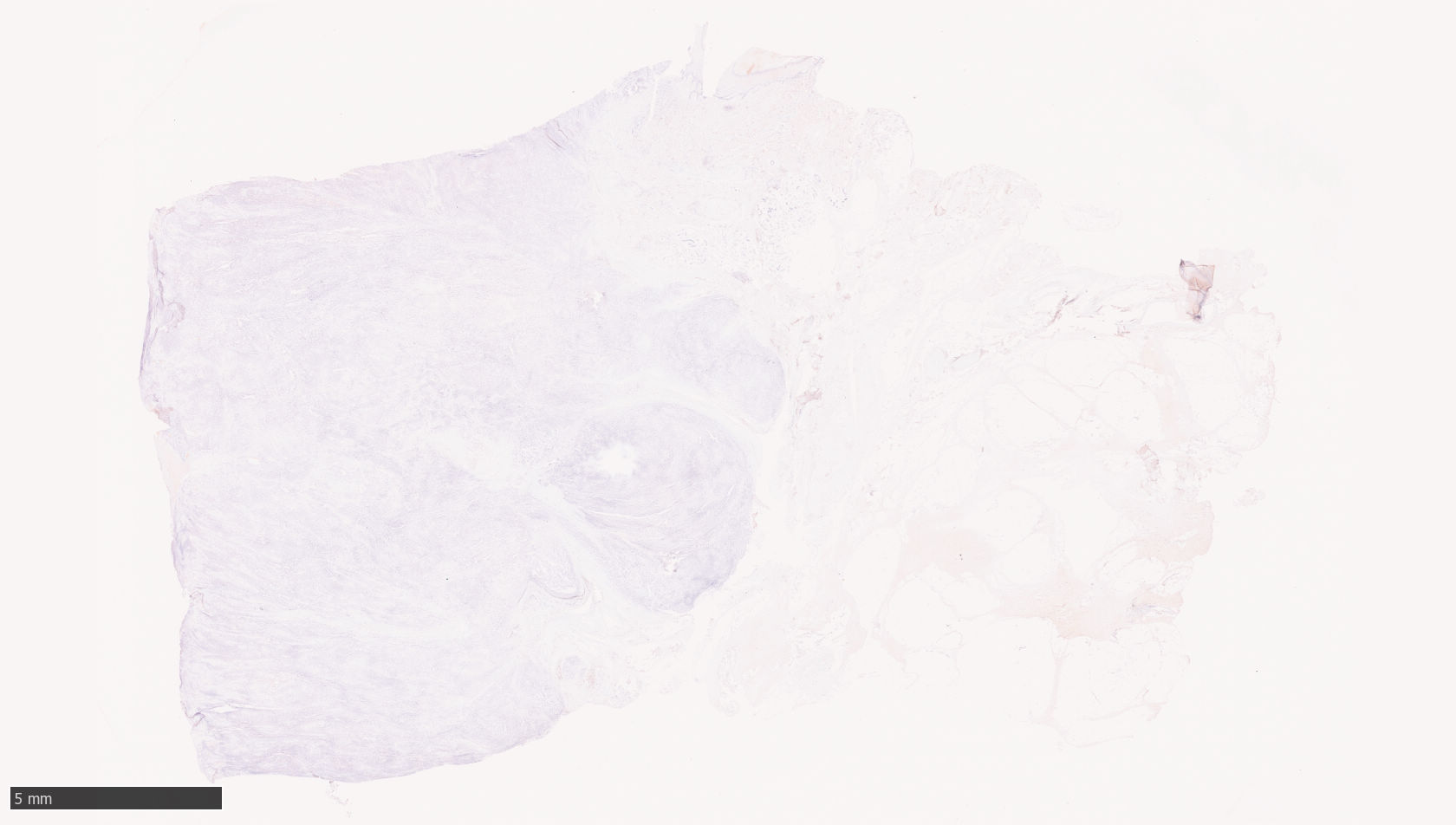


21-7818_IL18
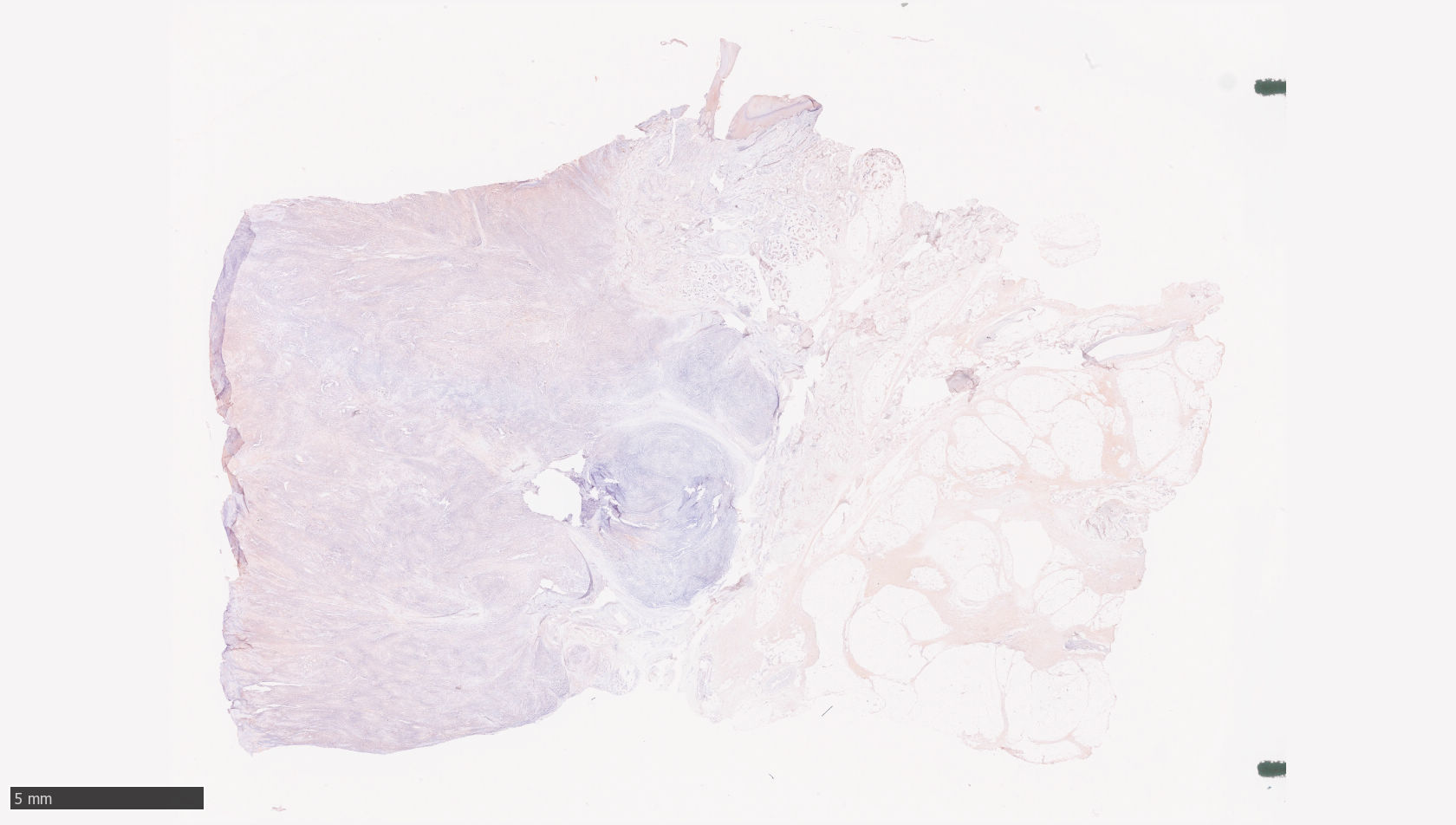


21-7818_NLRP1
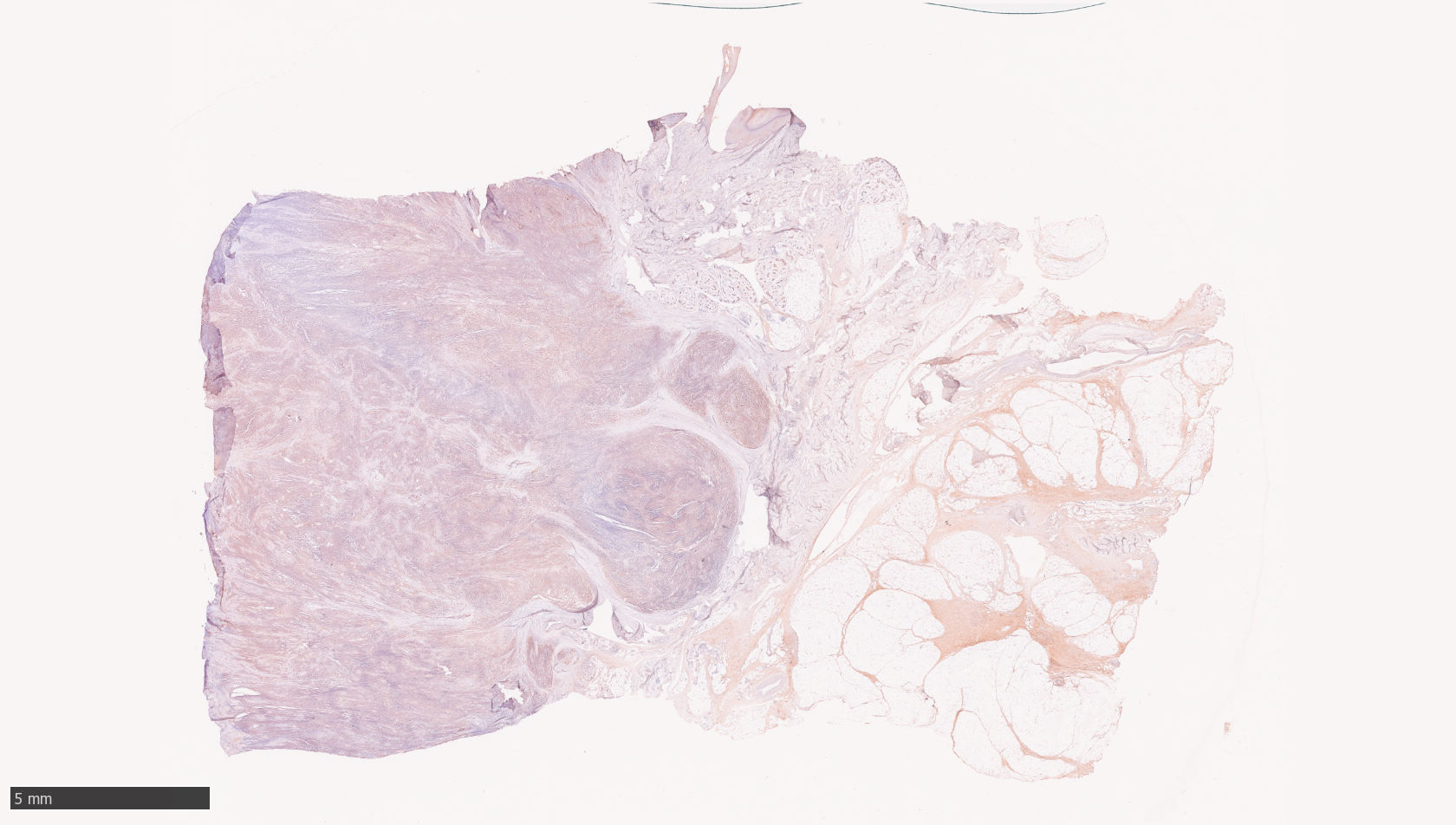


21-8530_CHMP4A
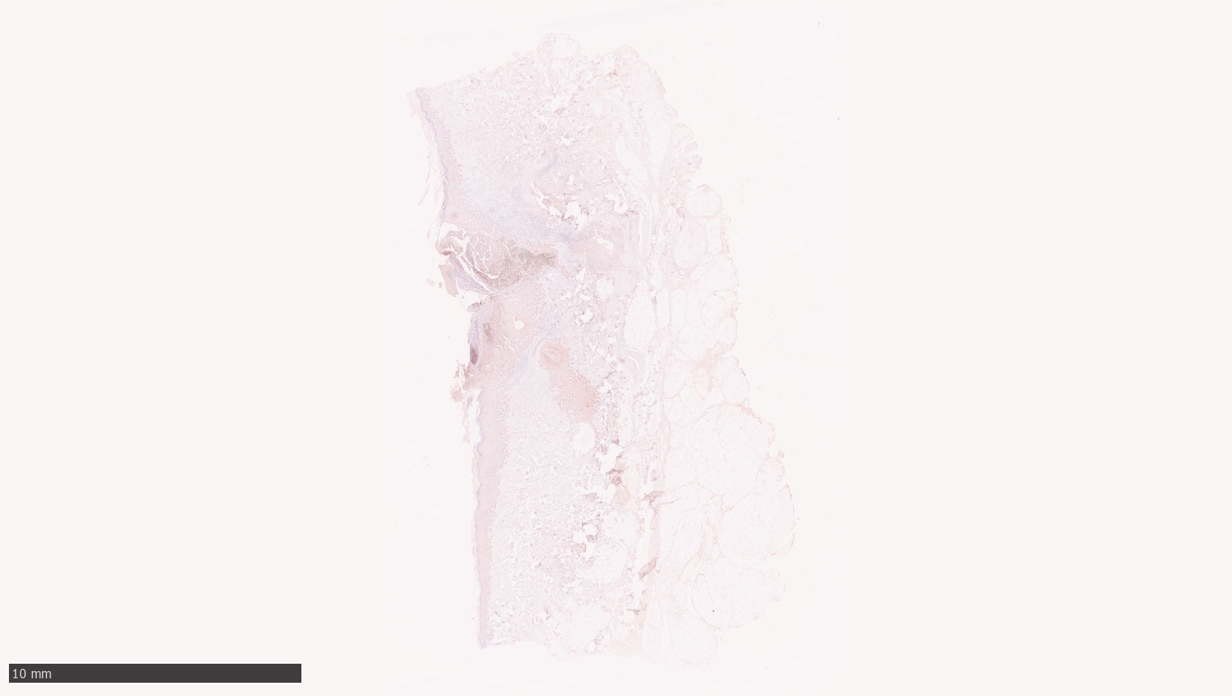


21-8530_GSDMB
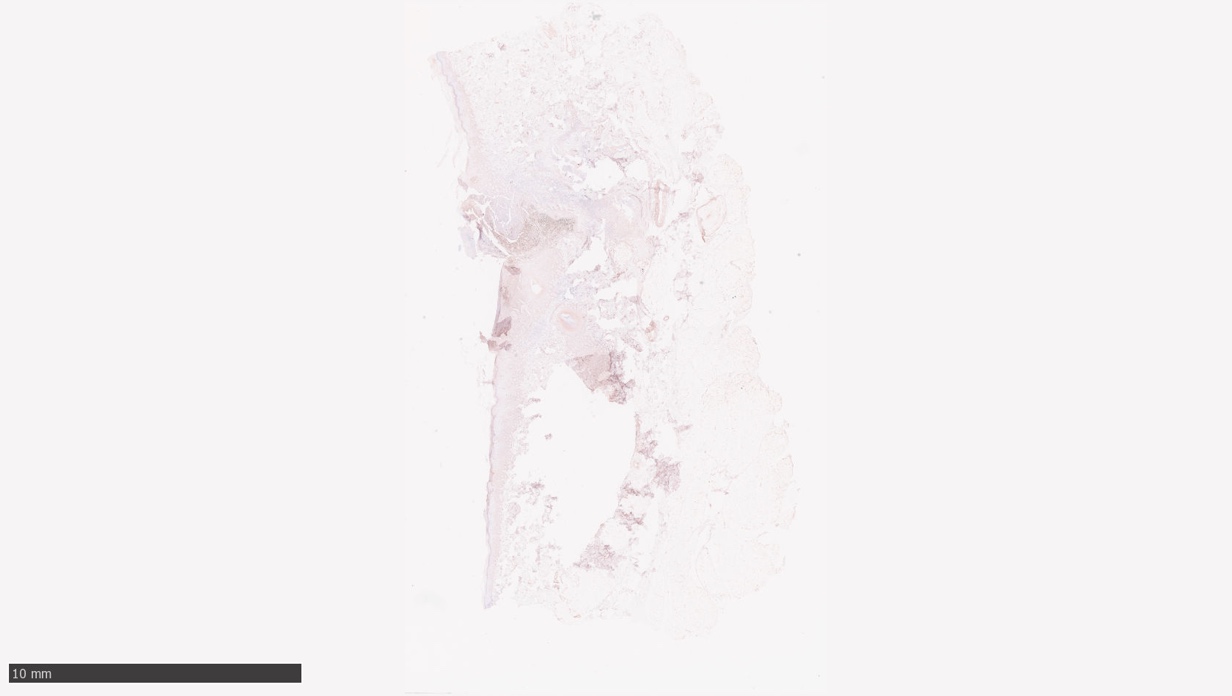


21-8530_GZMA
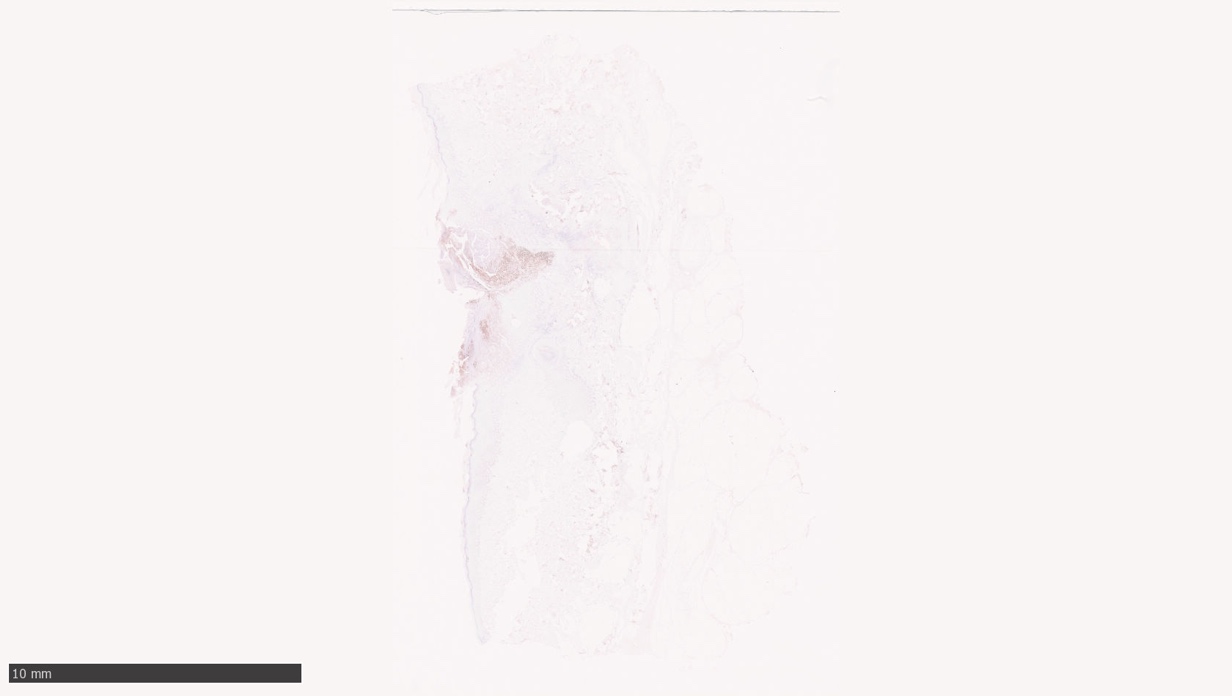


21-8530_IL18
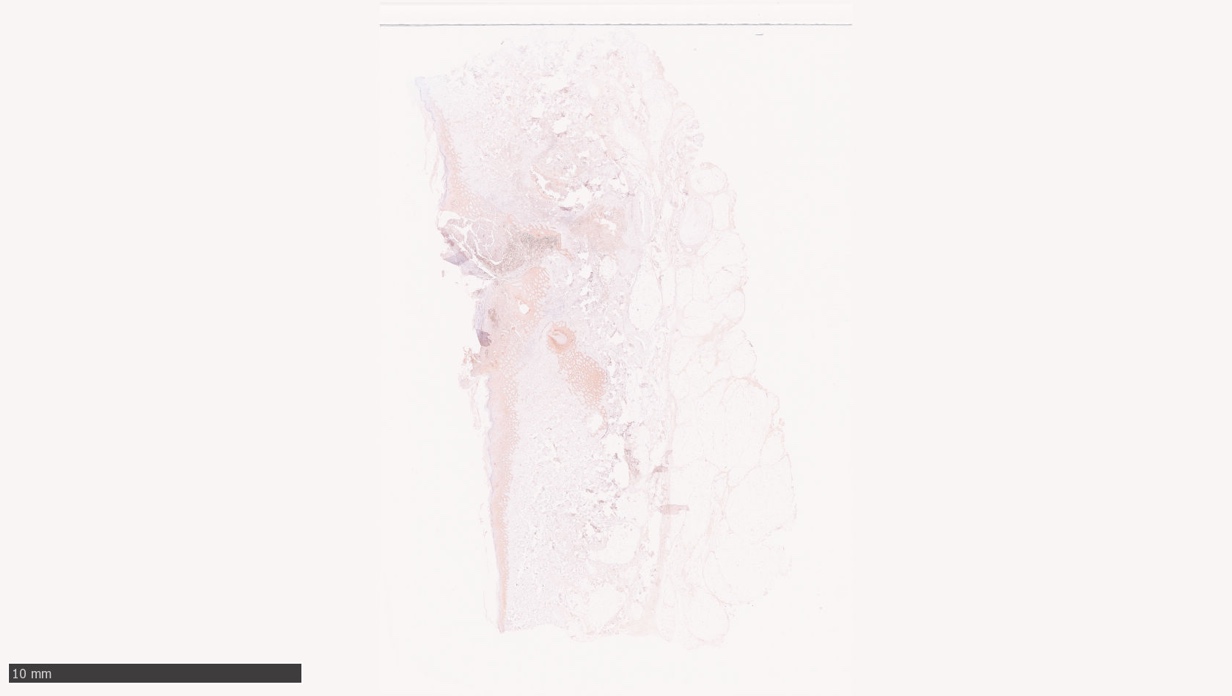


21-8530_NLRP1
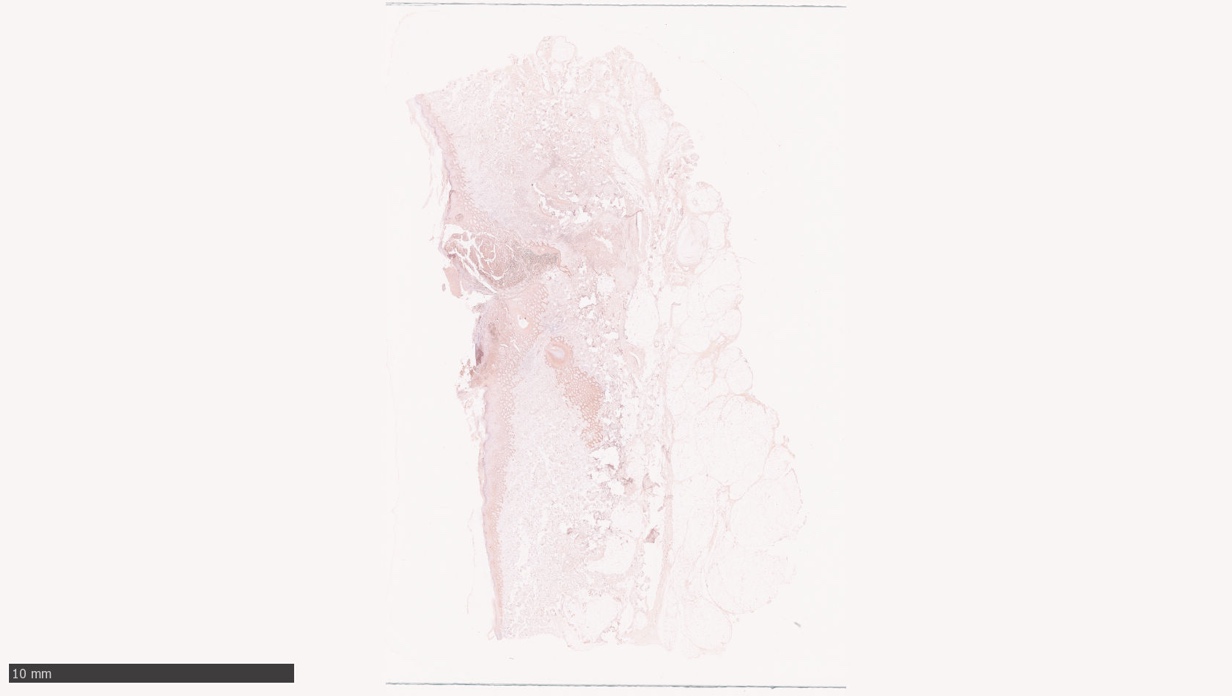


21-10026_CHMP4A
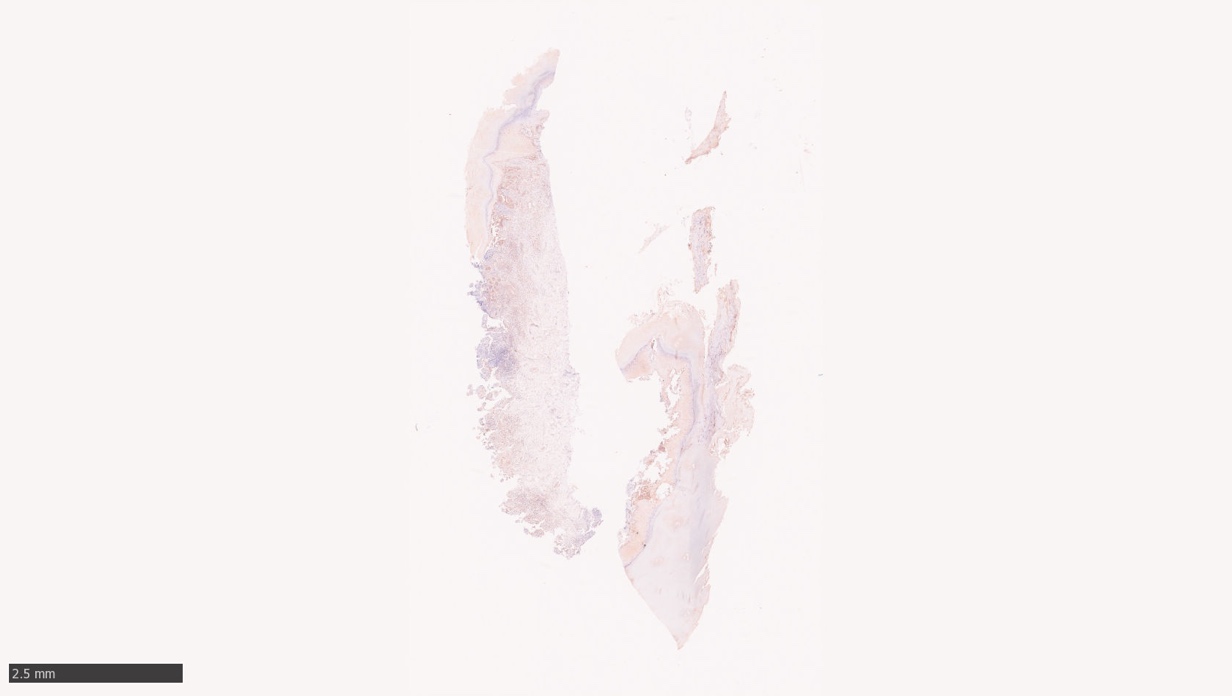


21-10026_GSDMB
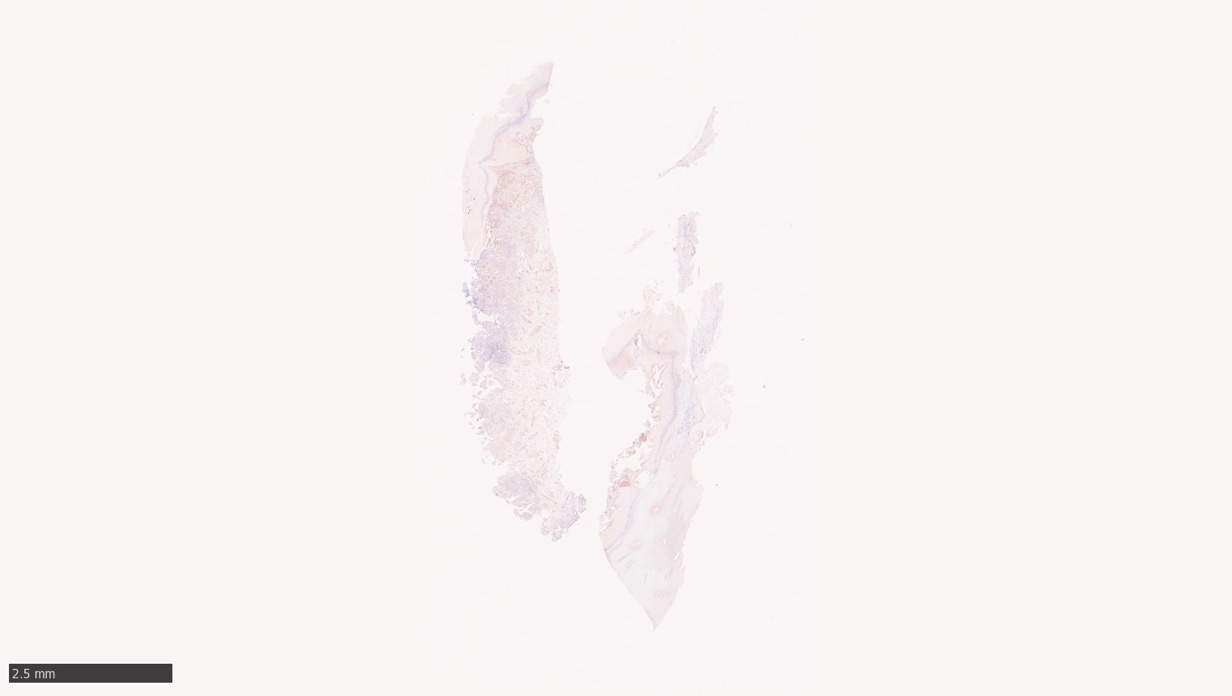
 21-10026_GZMA
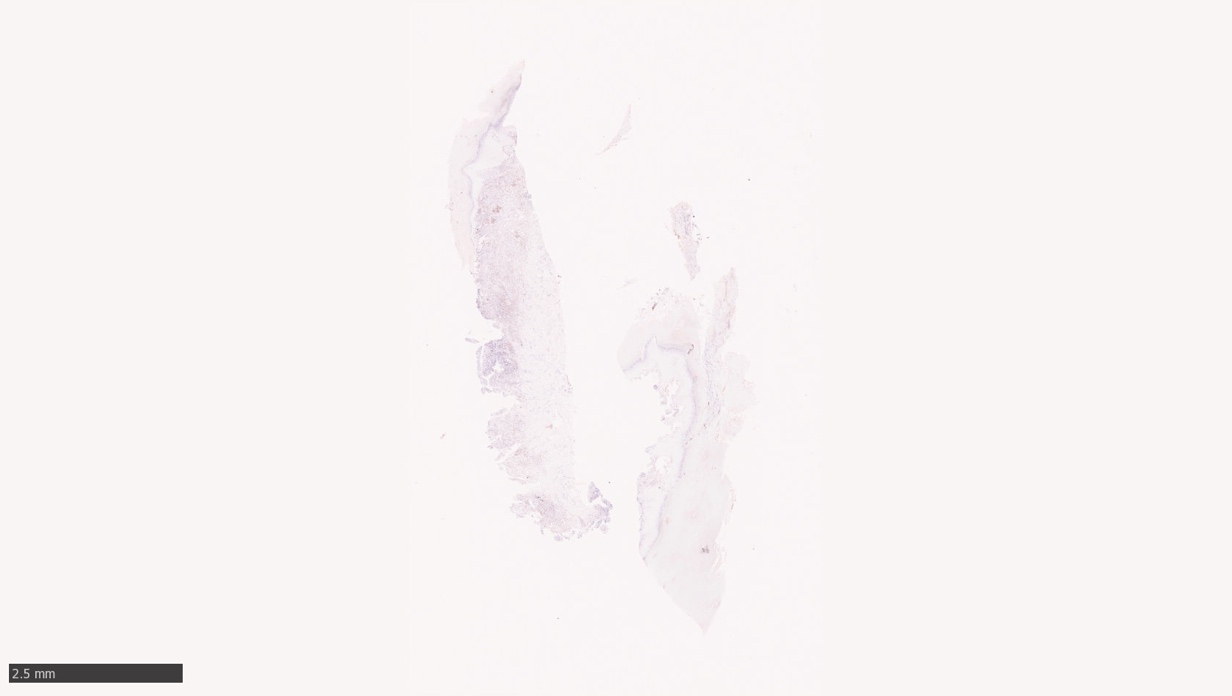


21-10026_IL18
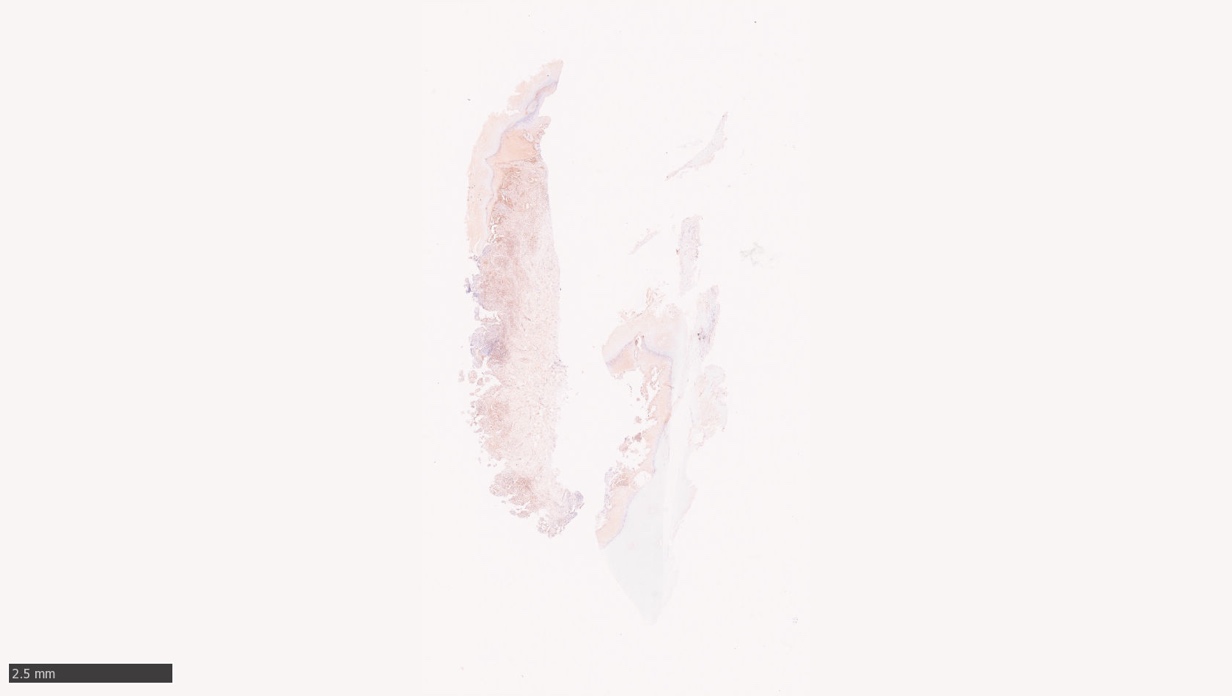
 21-10026_NLRP1
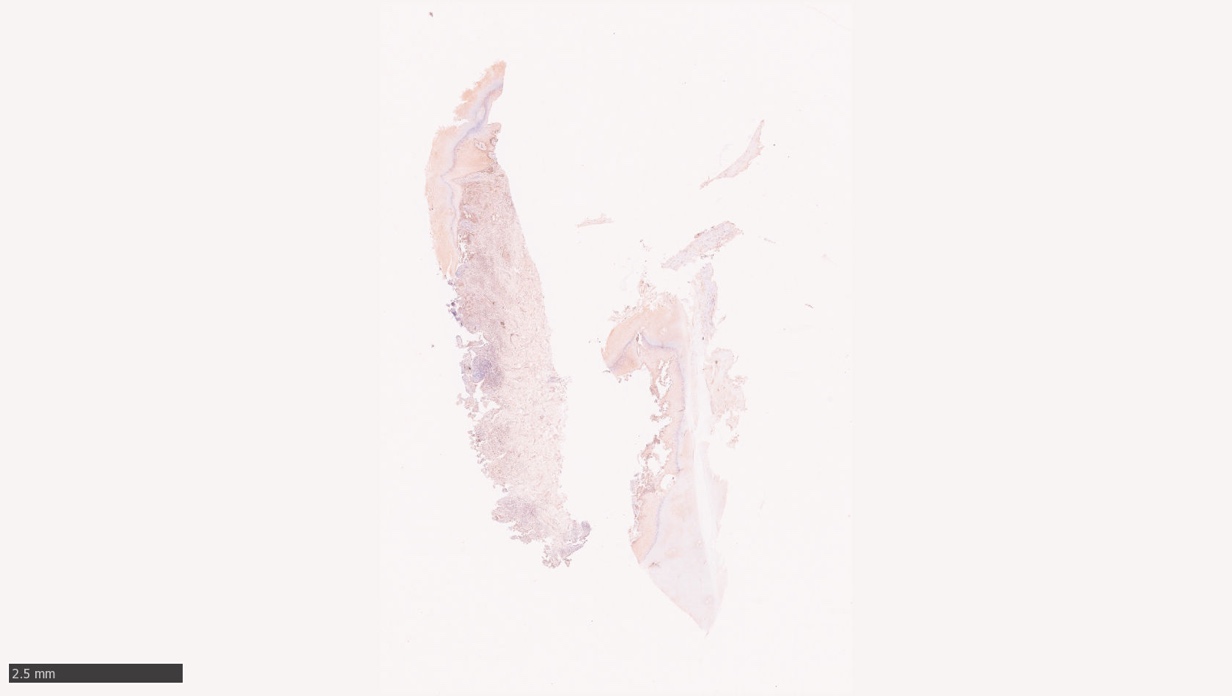


21-10457_CHMP4A
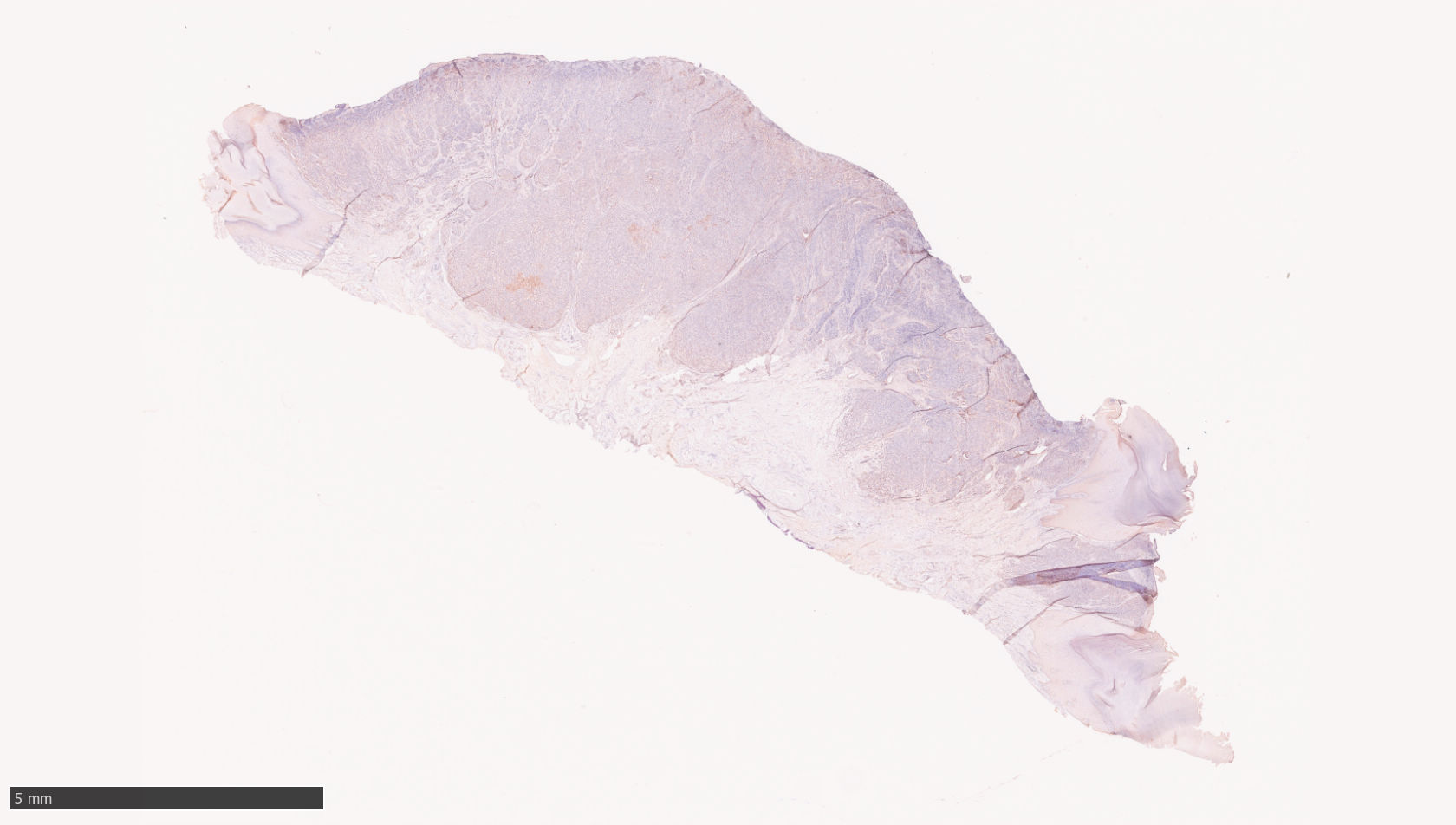
 21-10457_GSDMB
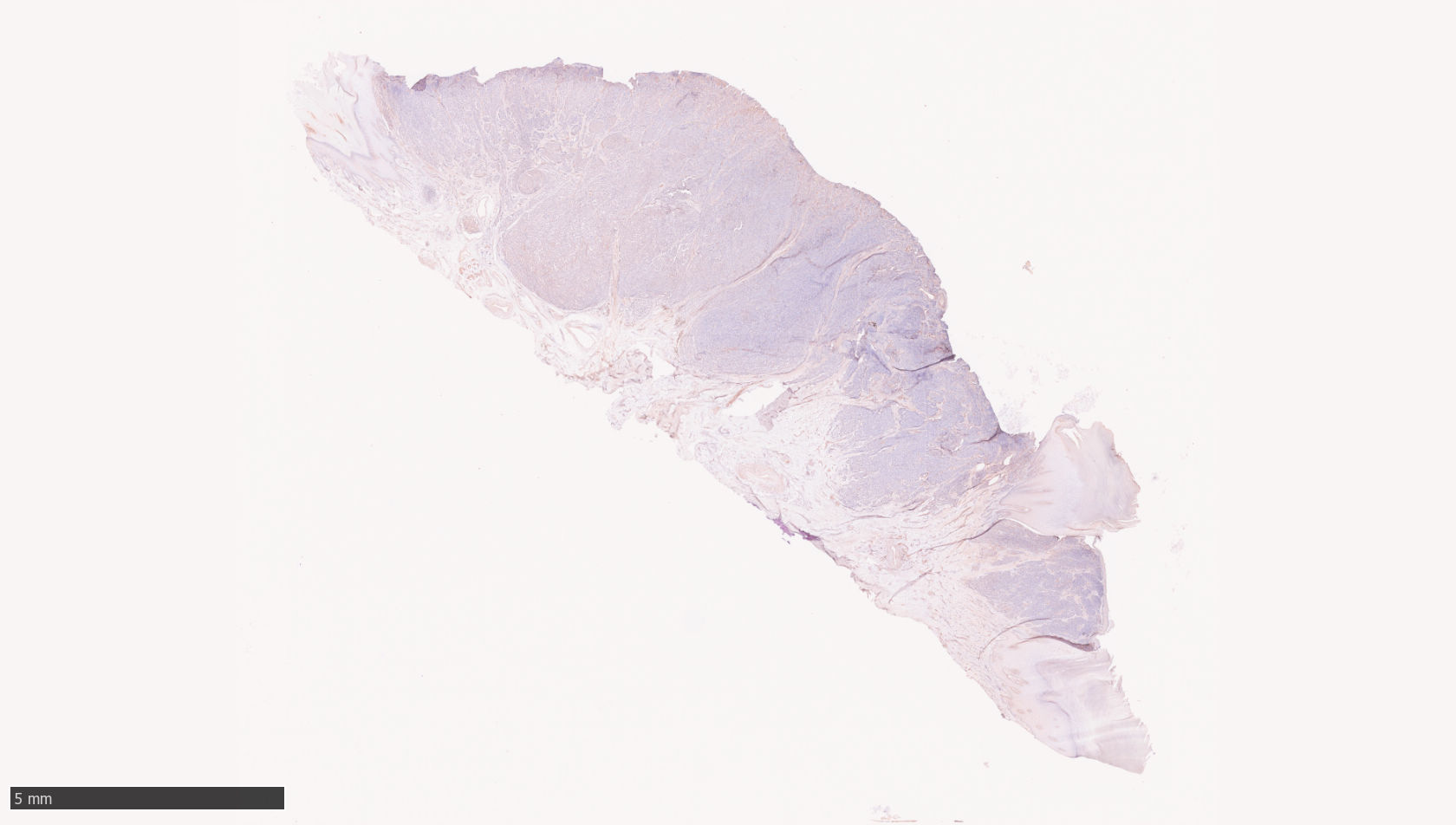


21-10457_GZMA
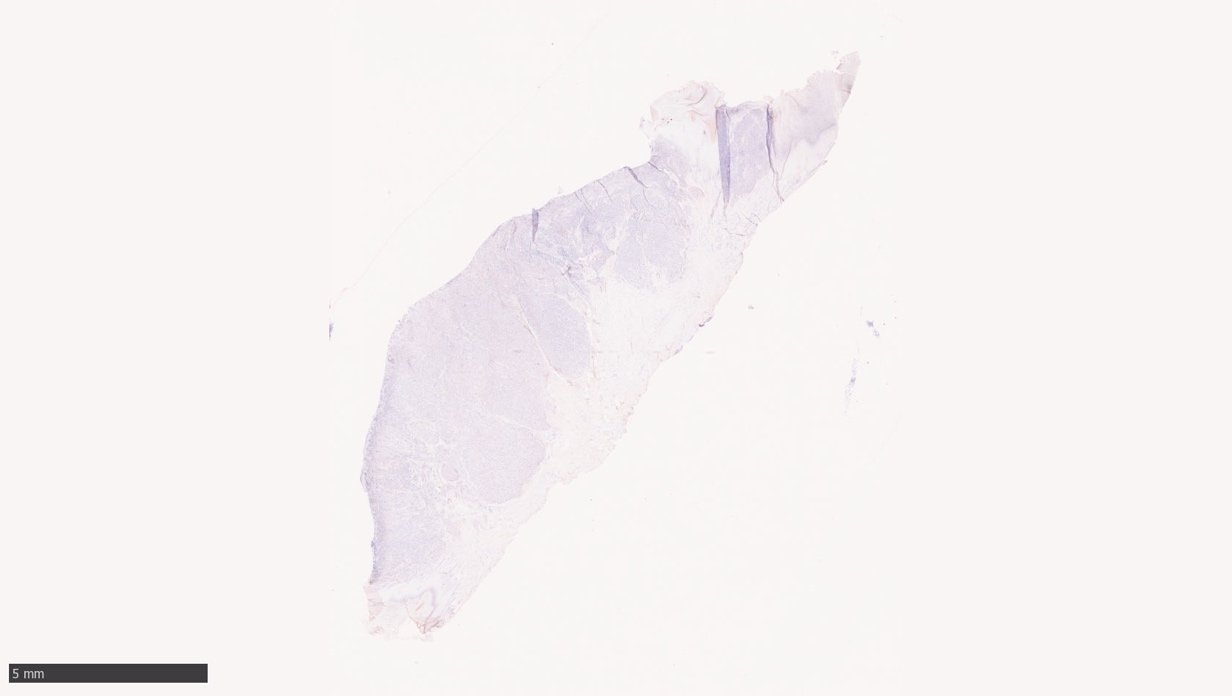
 21-10457_IL18
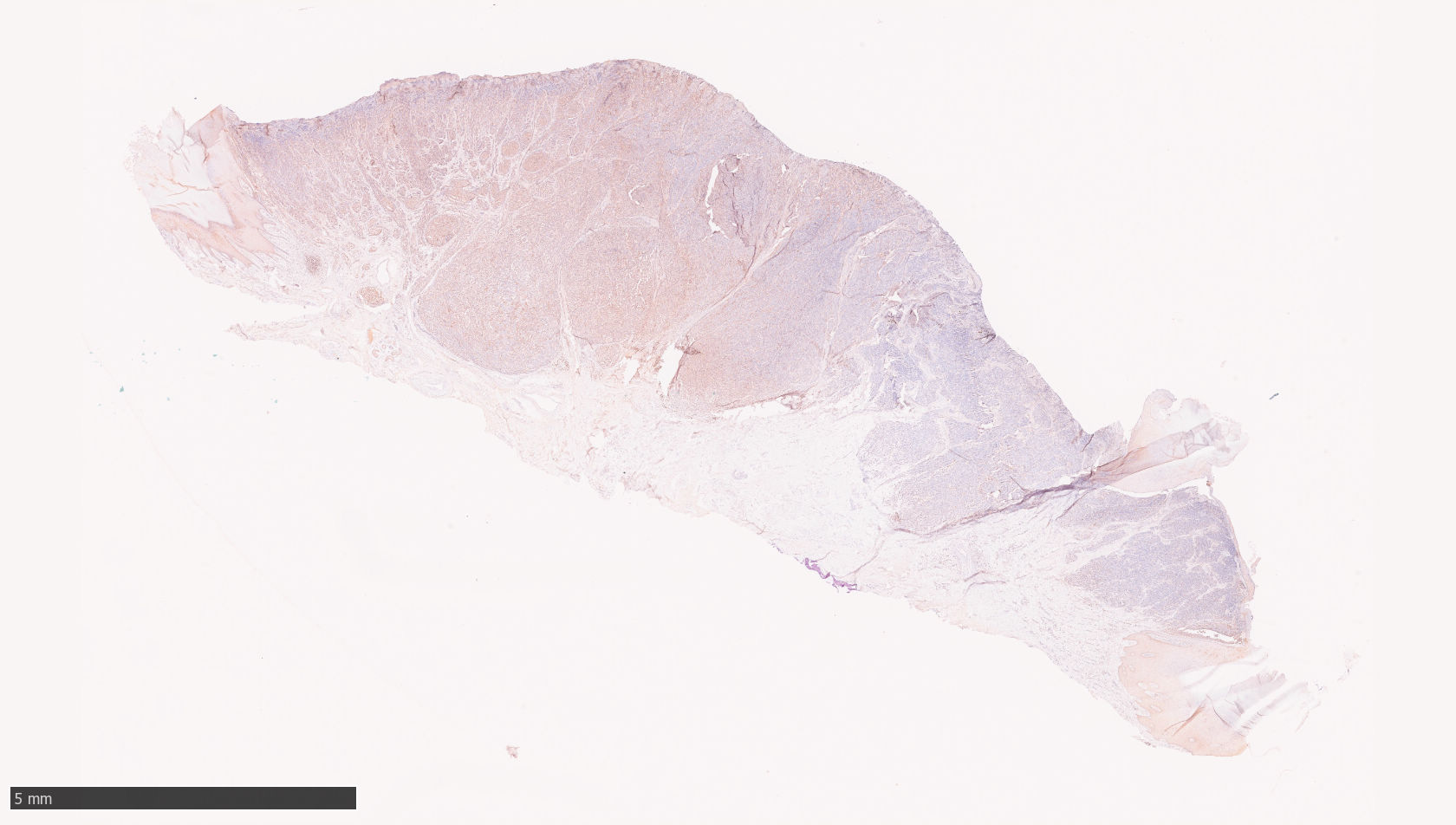


21-10457_NLRP1
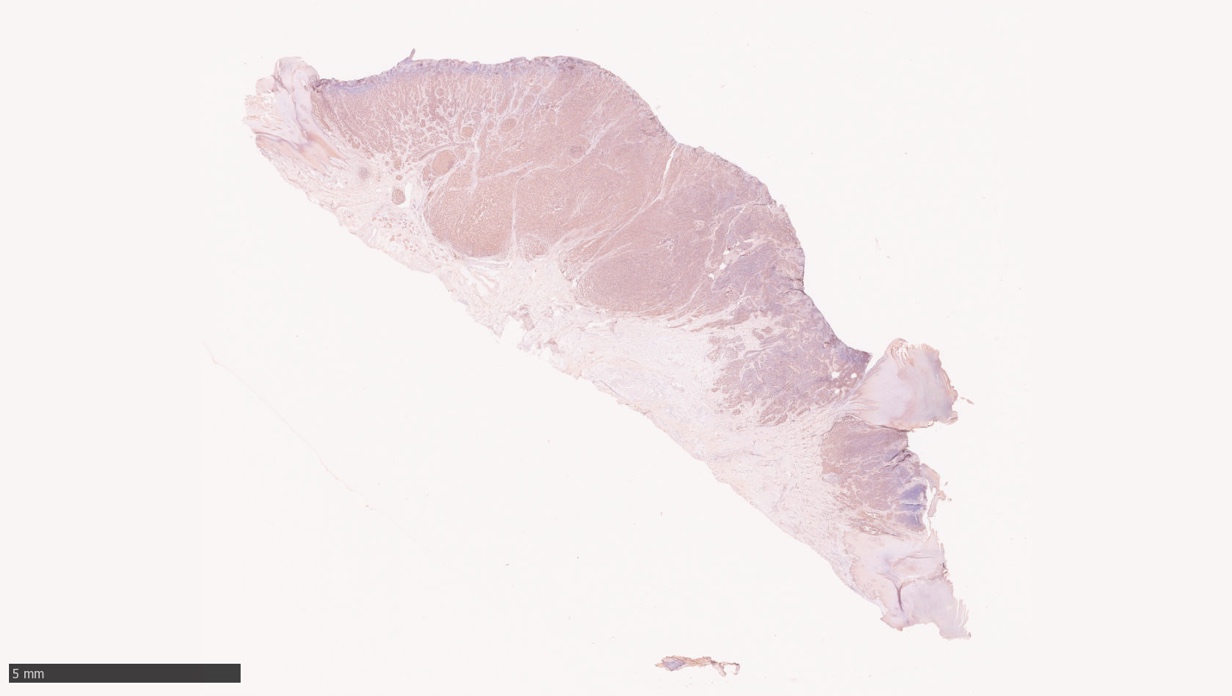
 CSX正49_CHMP4A


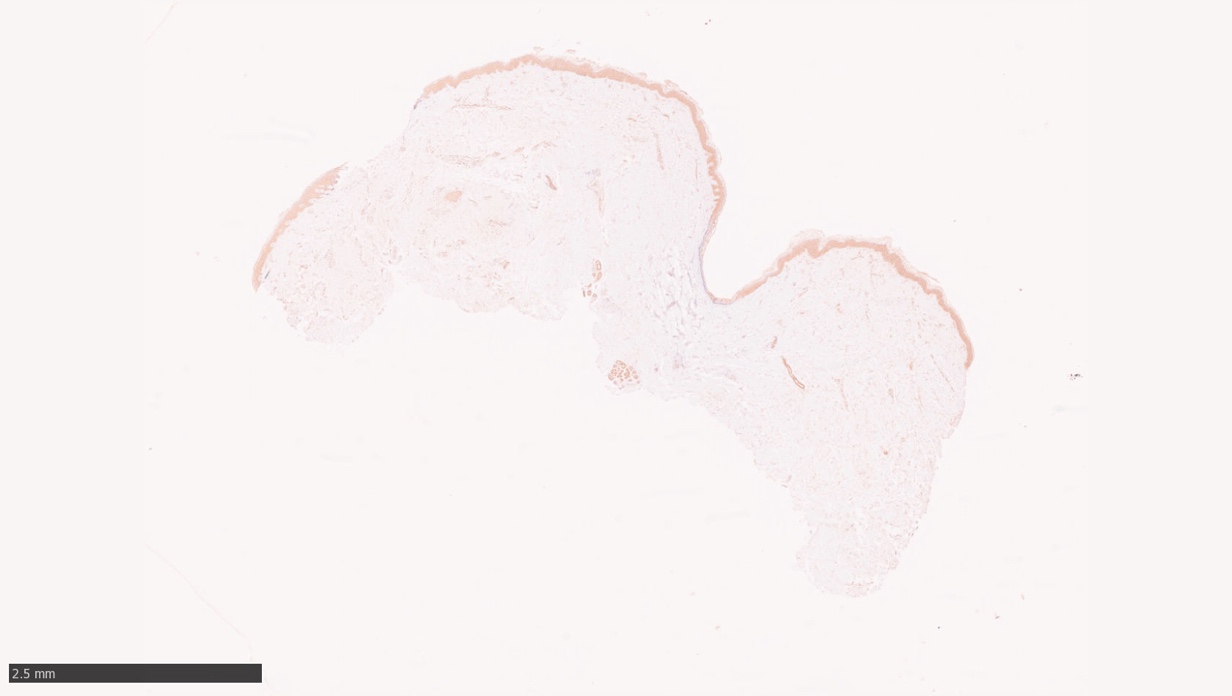


CSX正49_GSDMB


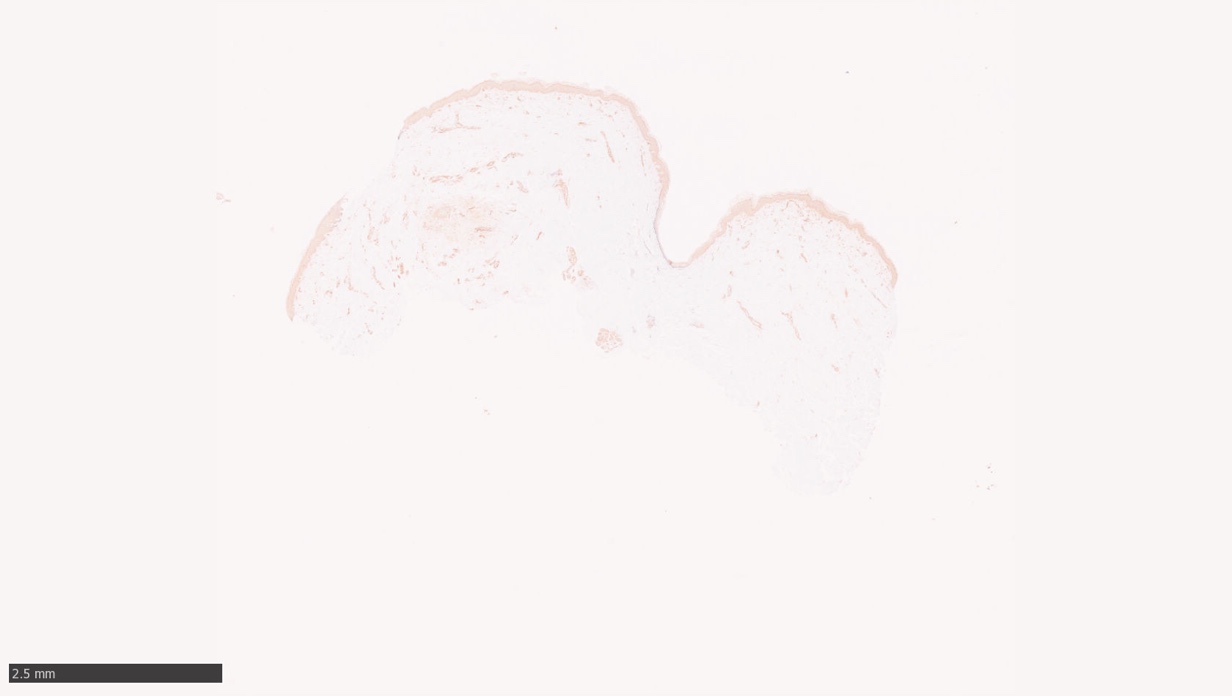


CSX正49_GZMA


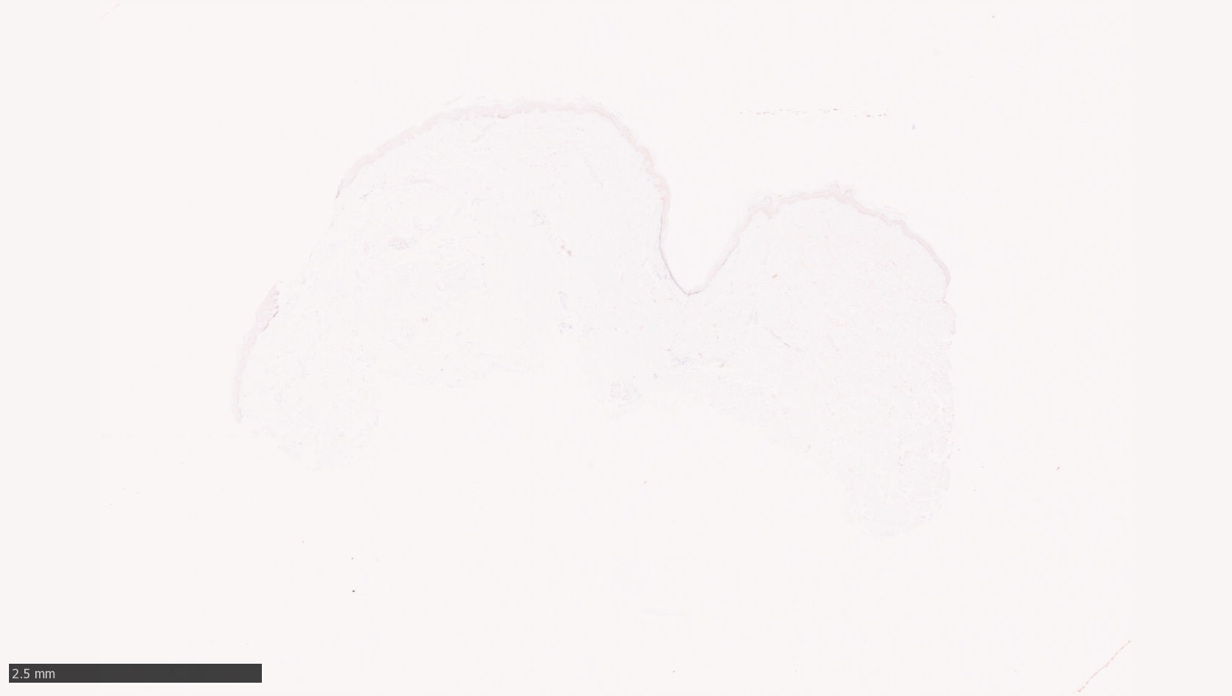


CSX正49_IL18


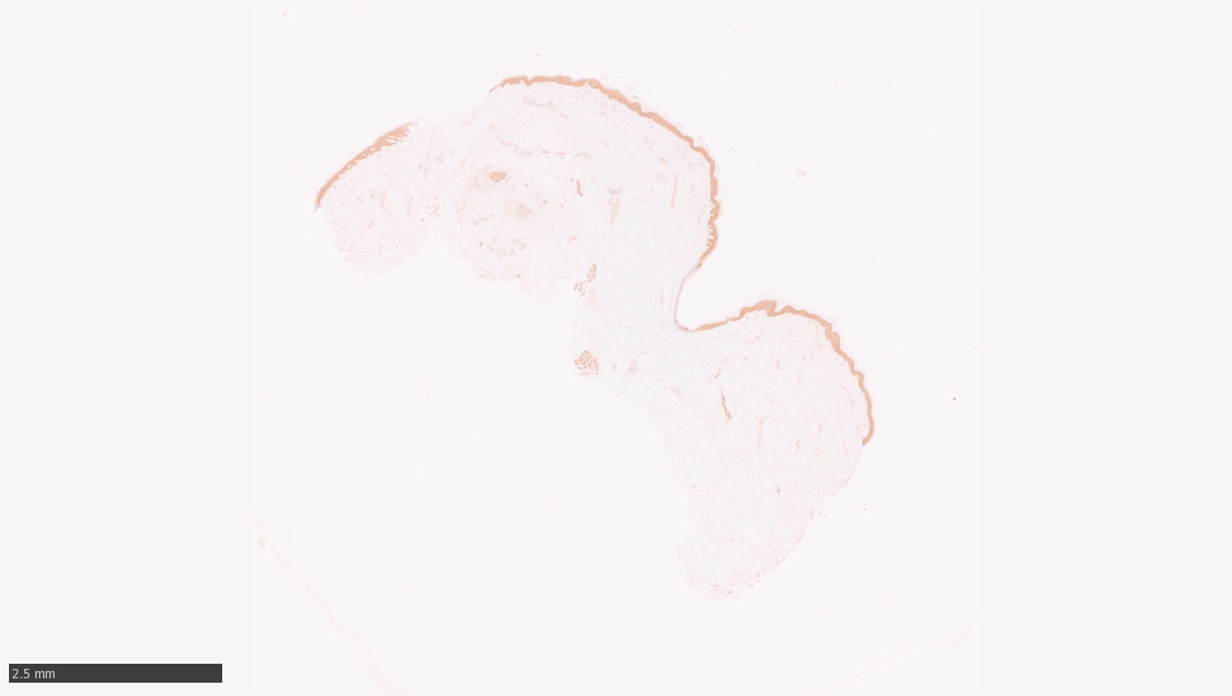


CSX正49_NLRP1


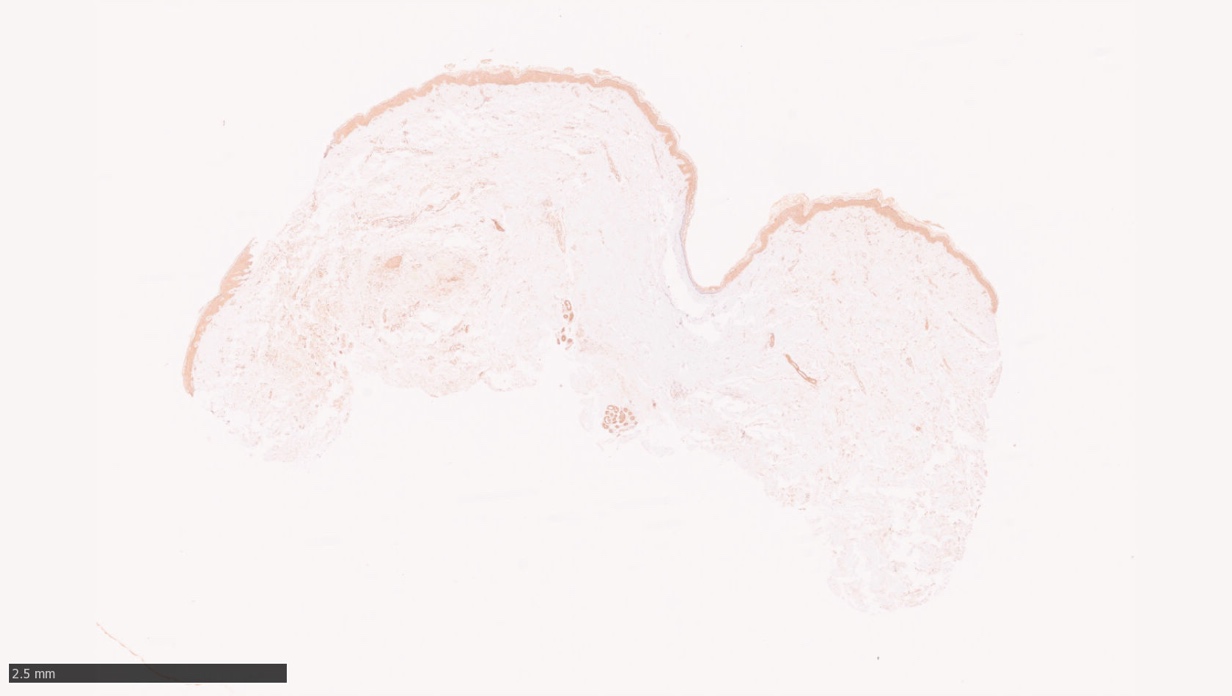


SNZ正58_CHMP4A


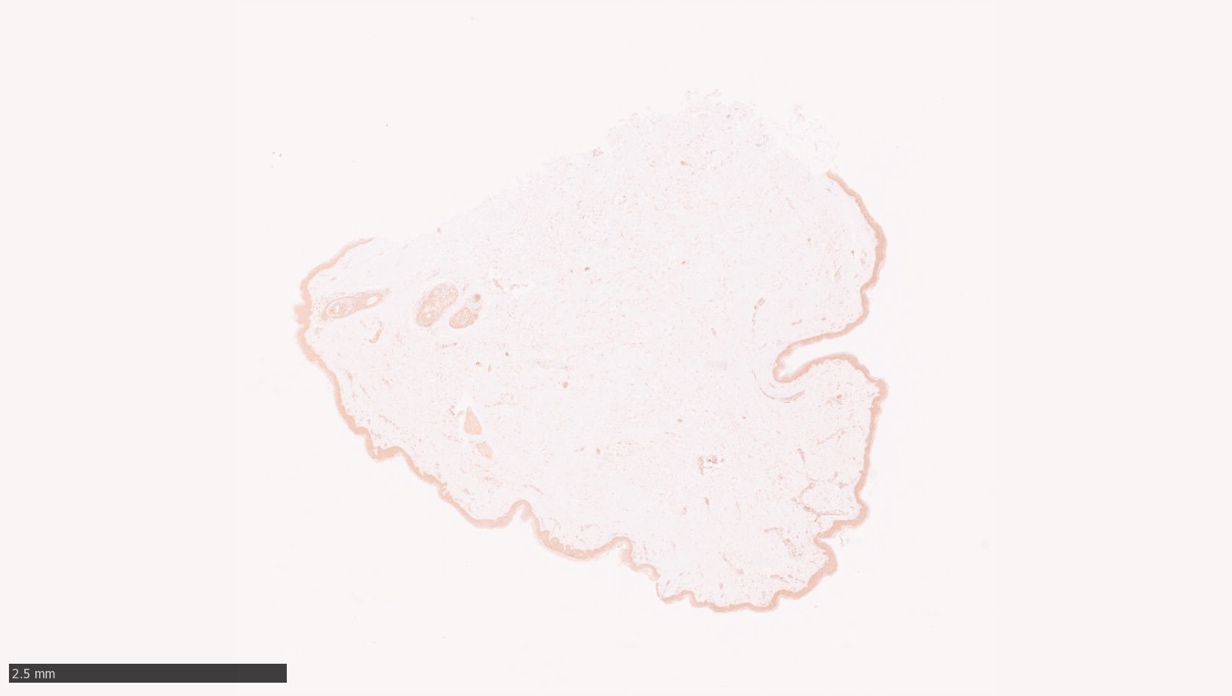
 SNZ正58_GSDMB


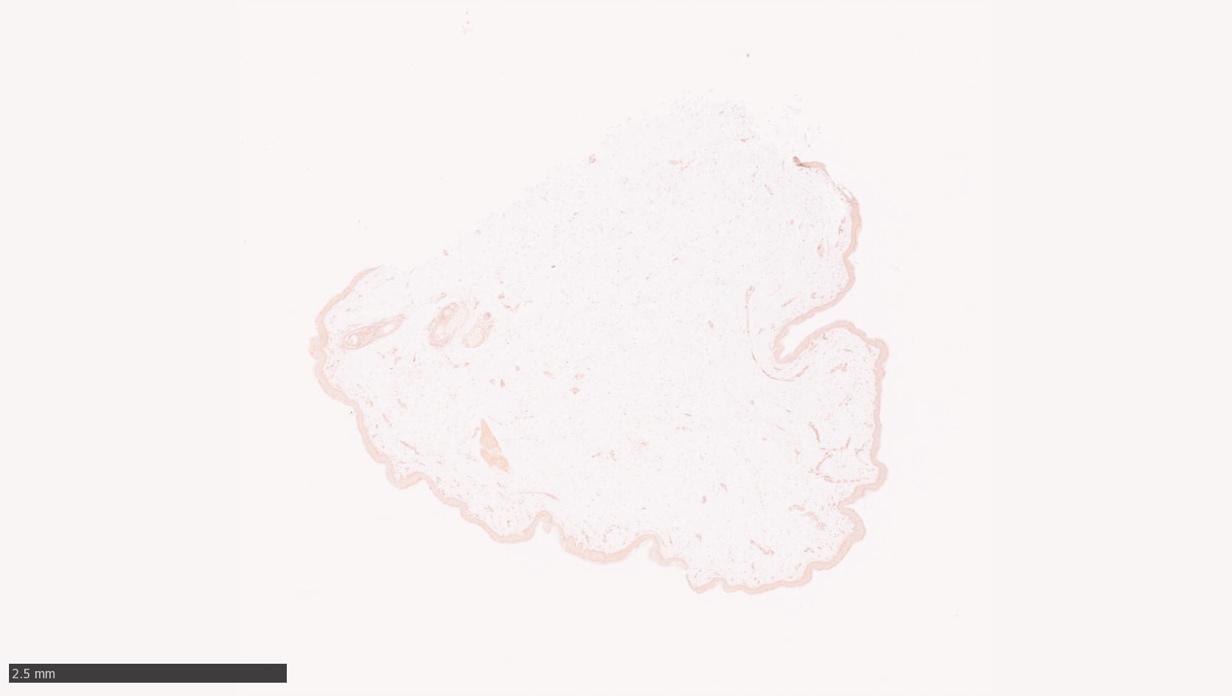


SNZ正58_GZMA


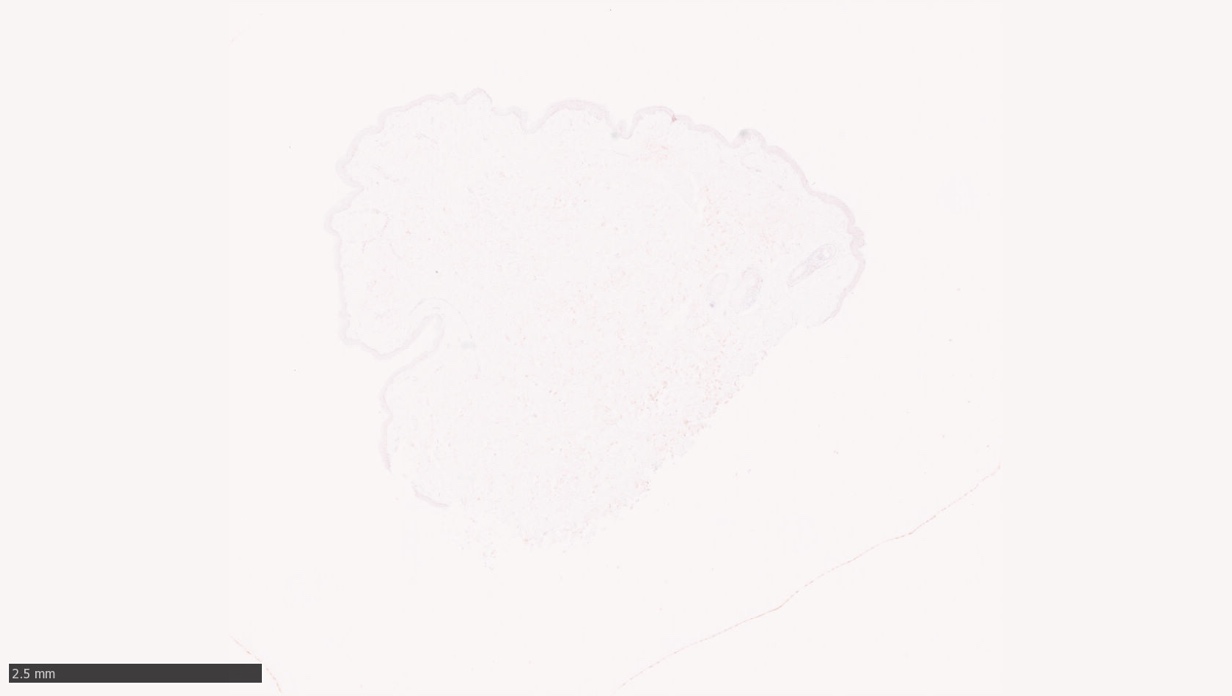
 SNZ正58_IL18


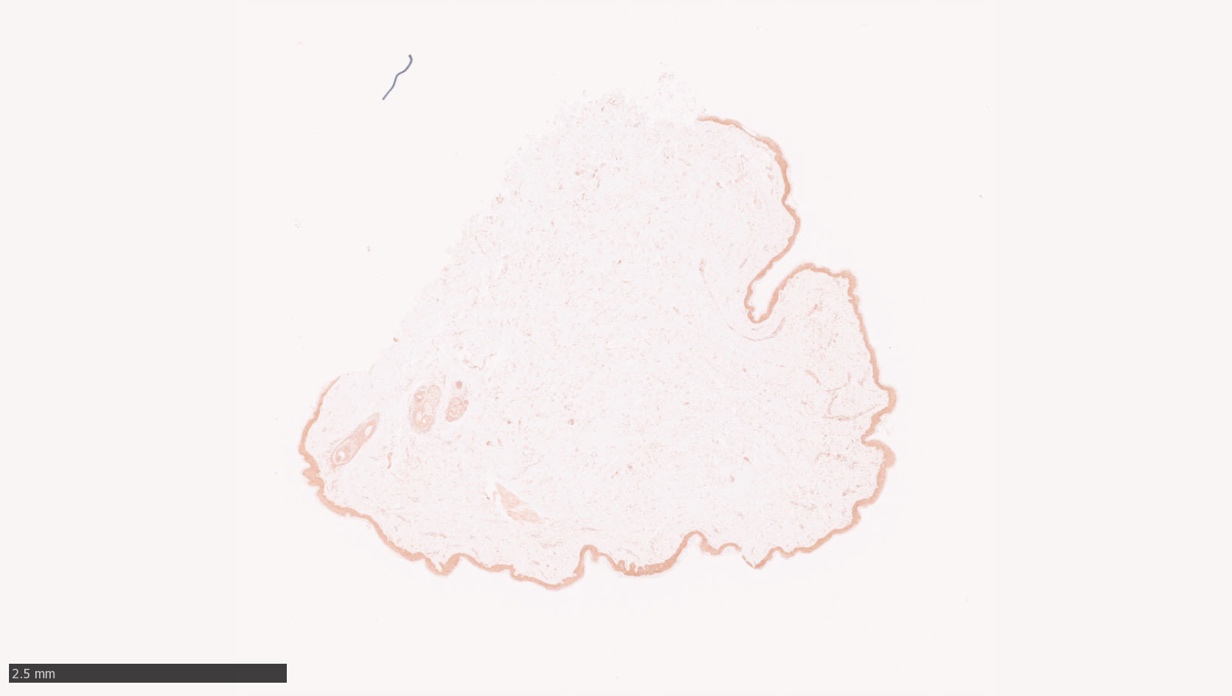


SNZ正58_NLRP1


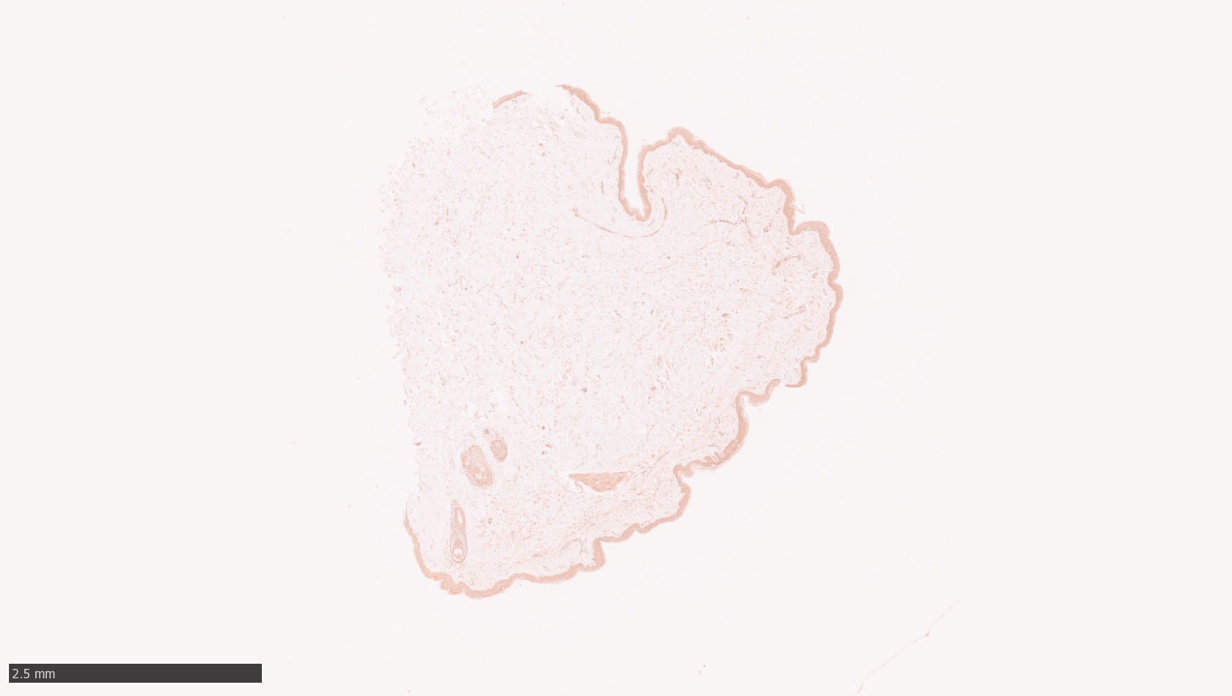
 WF正42_CHMP4A


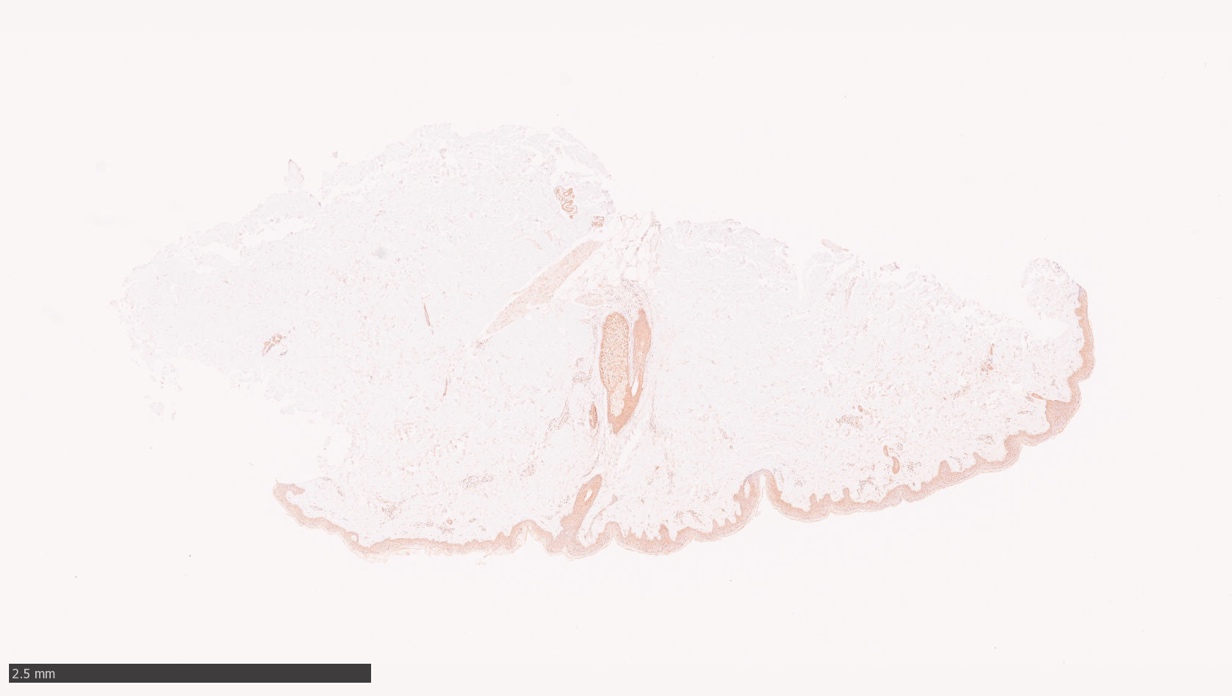


WF正42_GSDMB


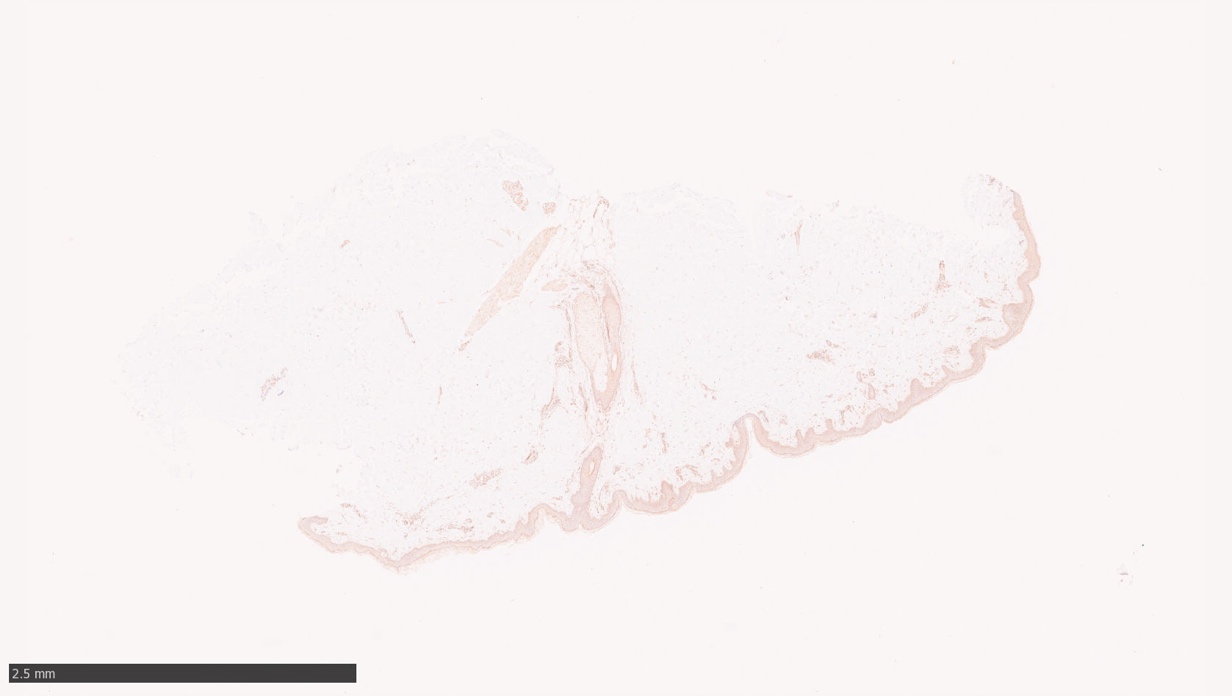
 WF正42_GZMA


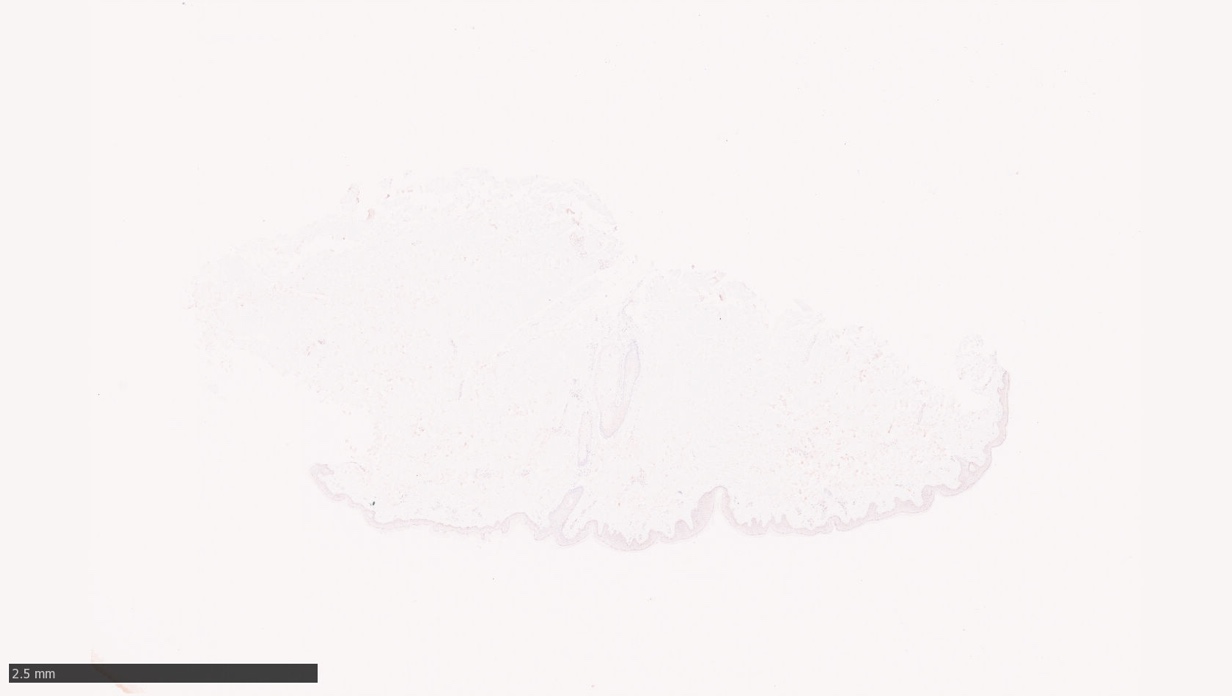


WF正42_IL18


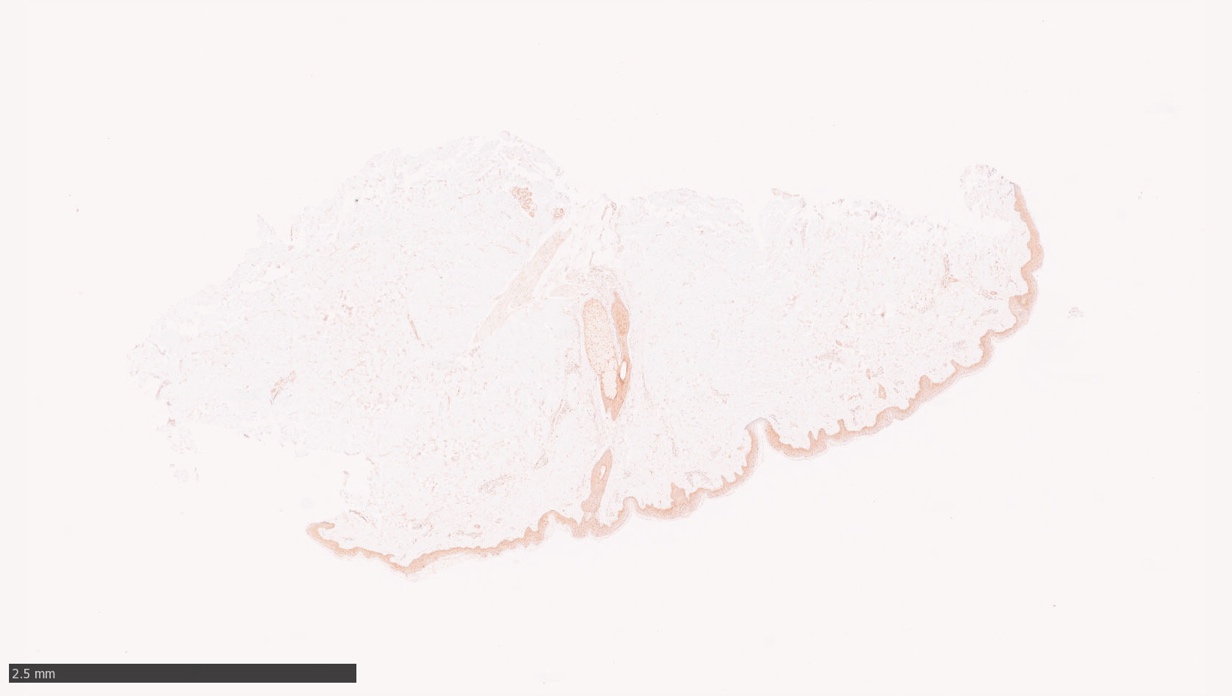
 WF正42_NLRP1


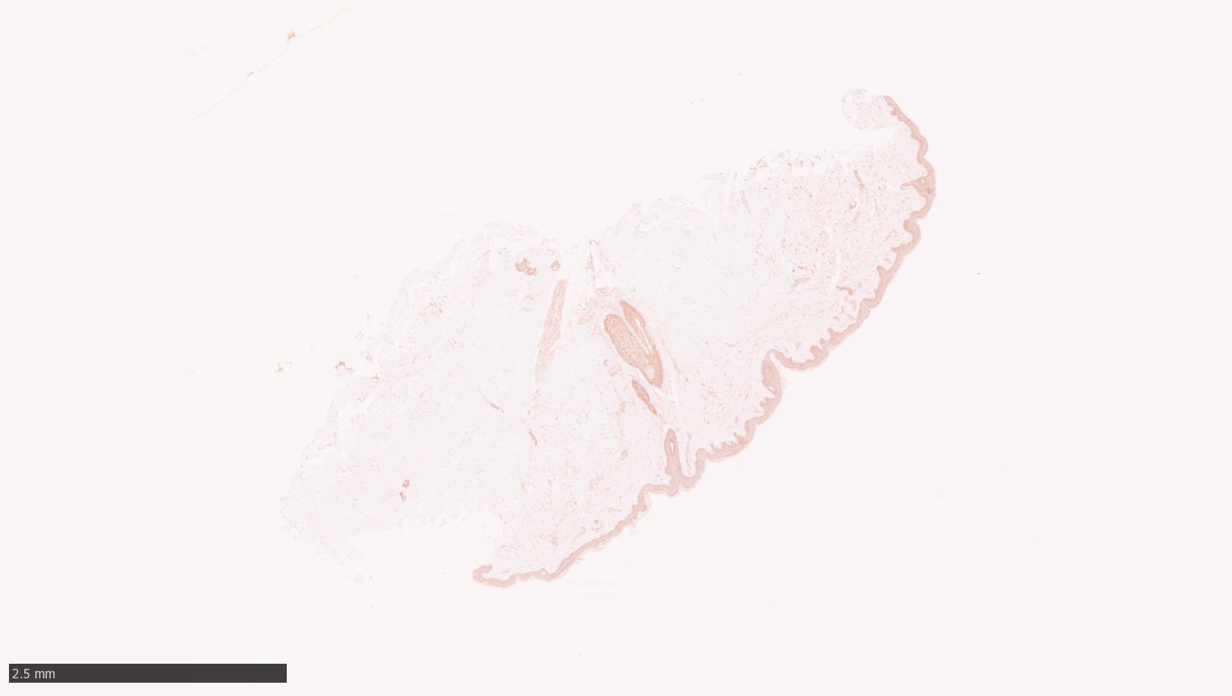


WXJ正43_CHMP4A


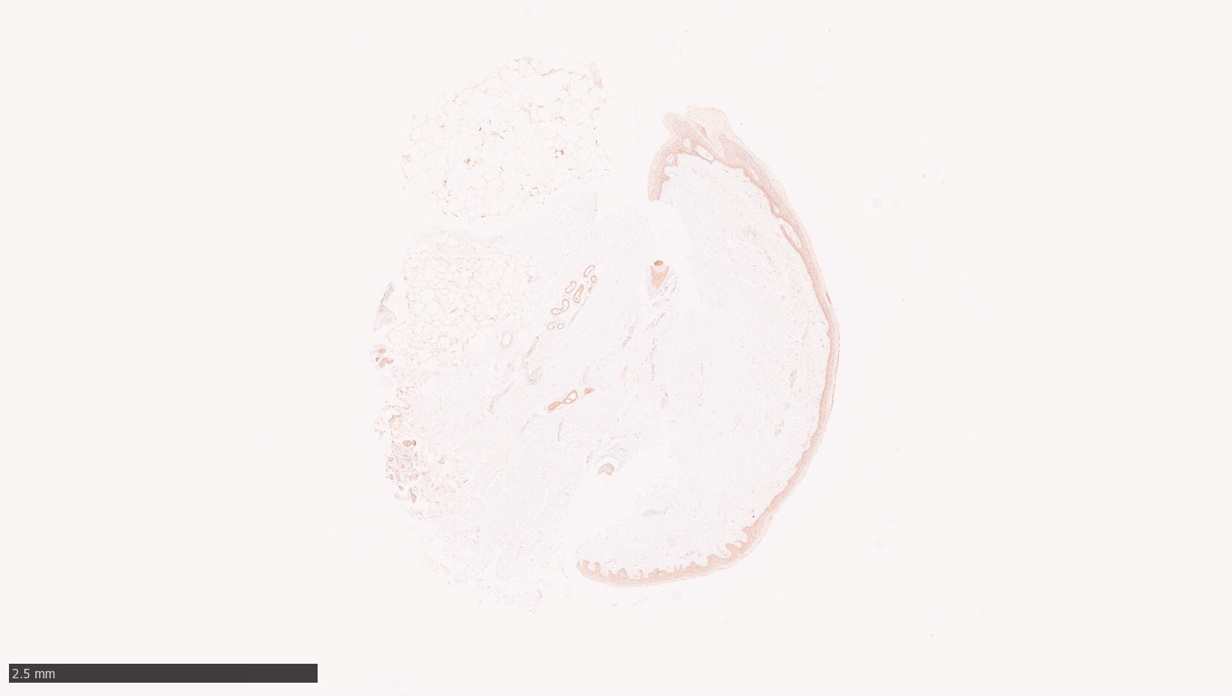
 WXJ正43_GSDMB


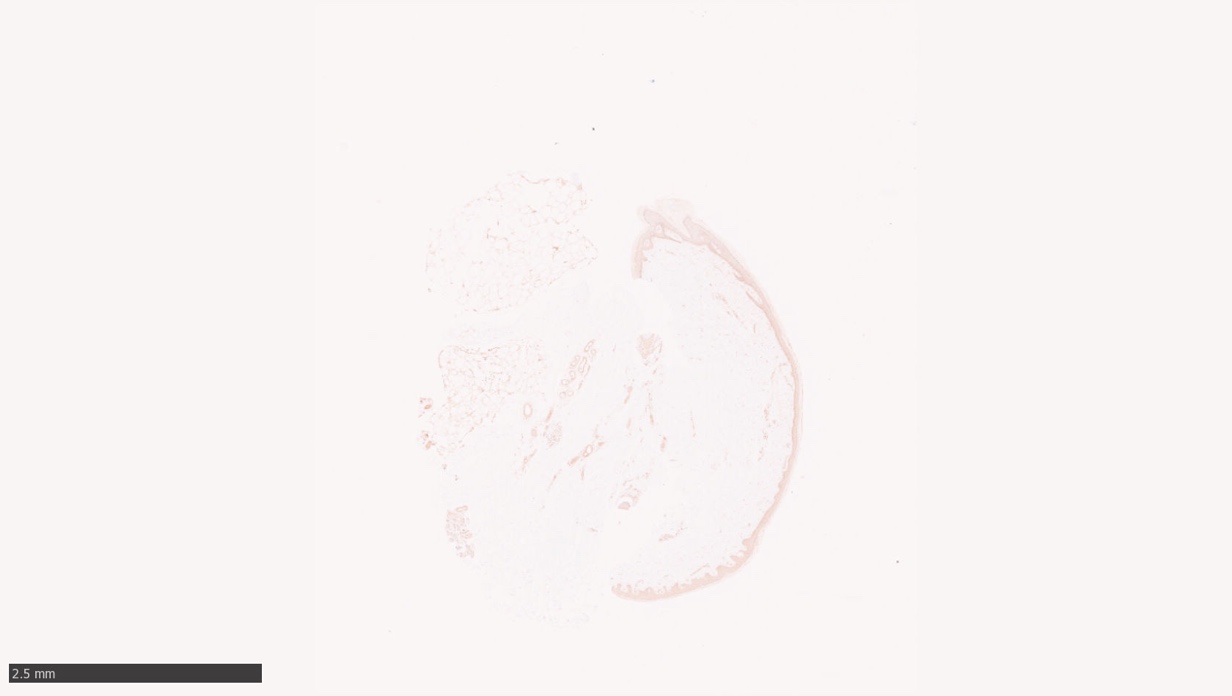


WXJ正43_GZMA


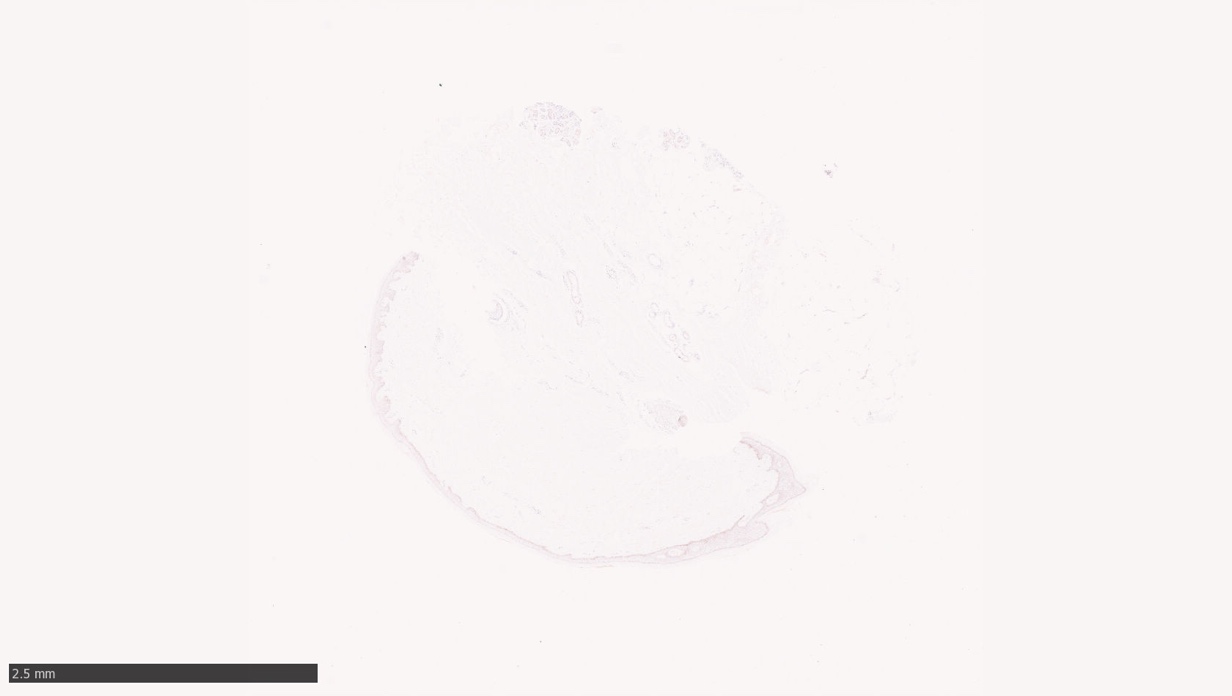
 WXJ正43_IL18


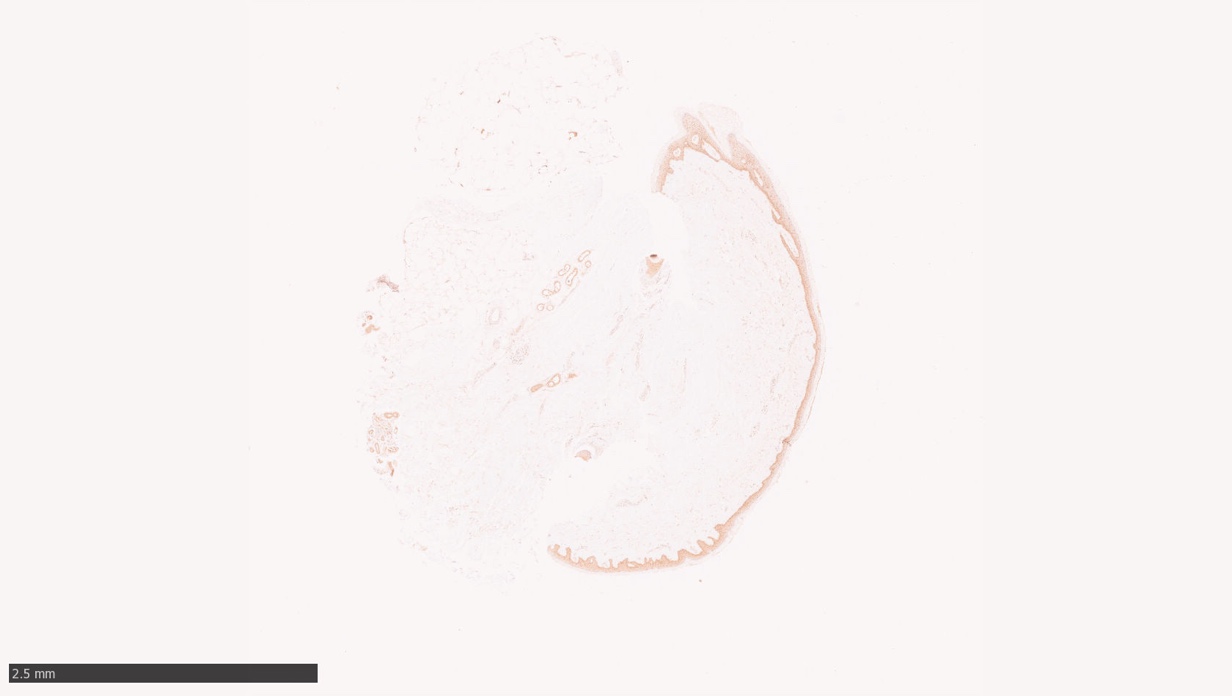


WXJ正43_NLRP1


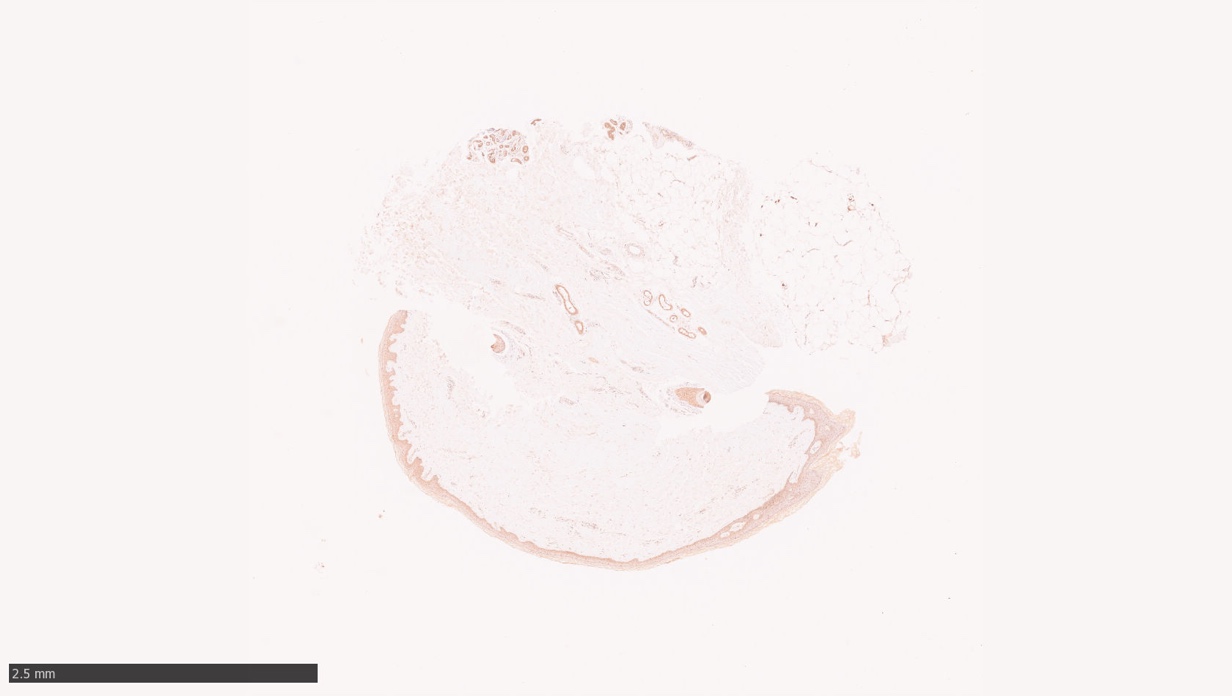


XH正47_CHMP4A


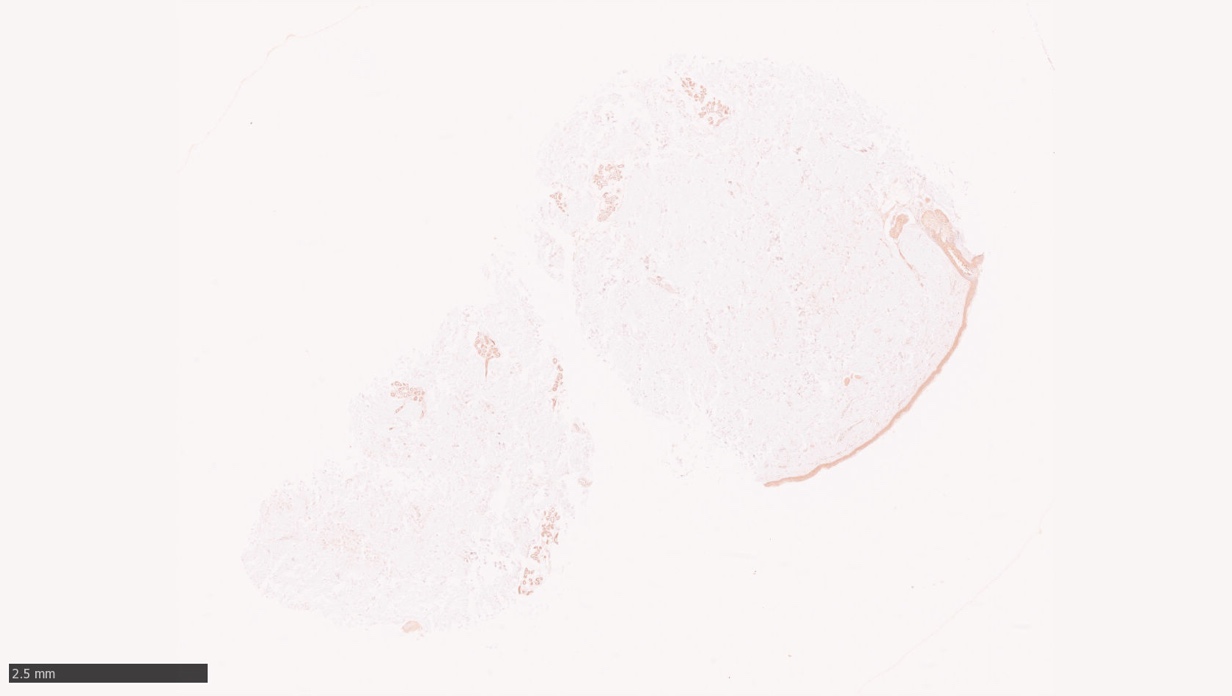


XH正47_GSDMB


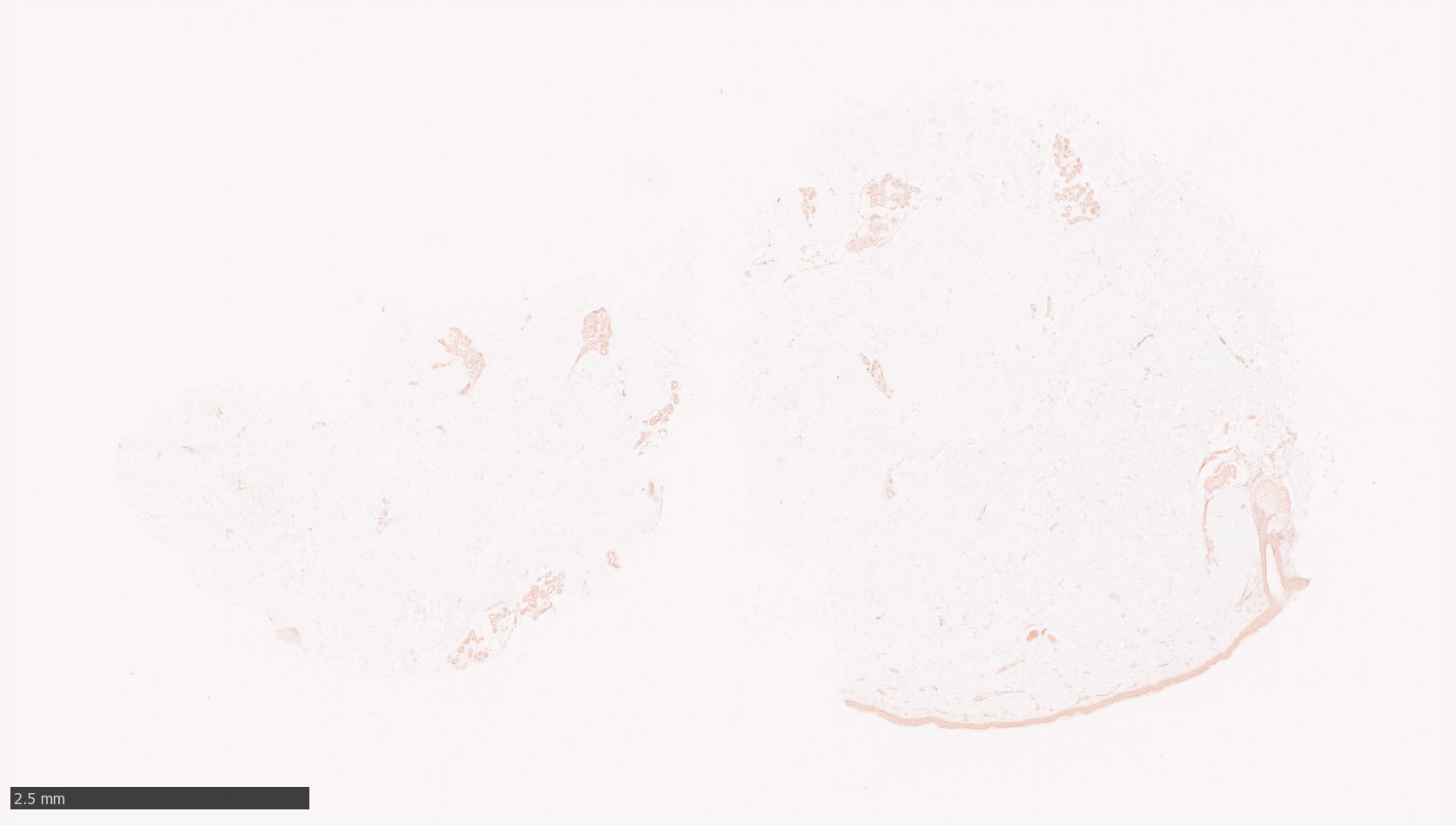
 XH正47_GZMA


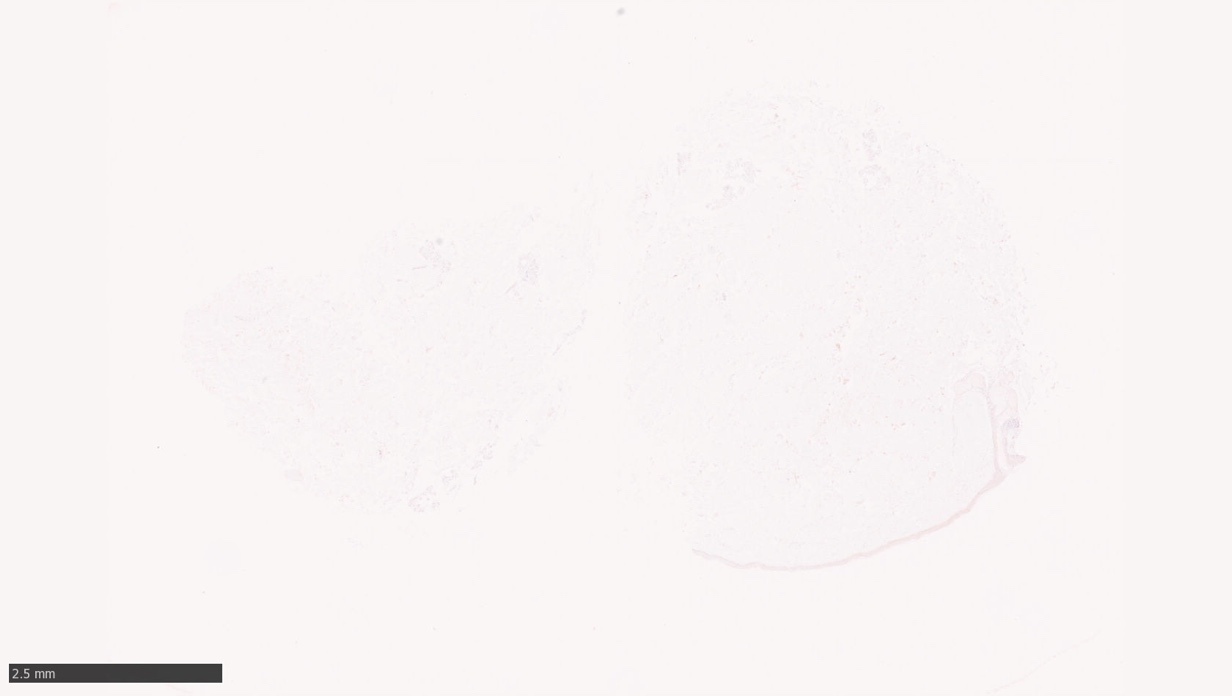


XH正47_IL18


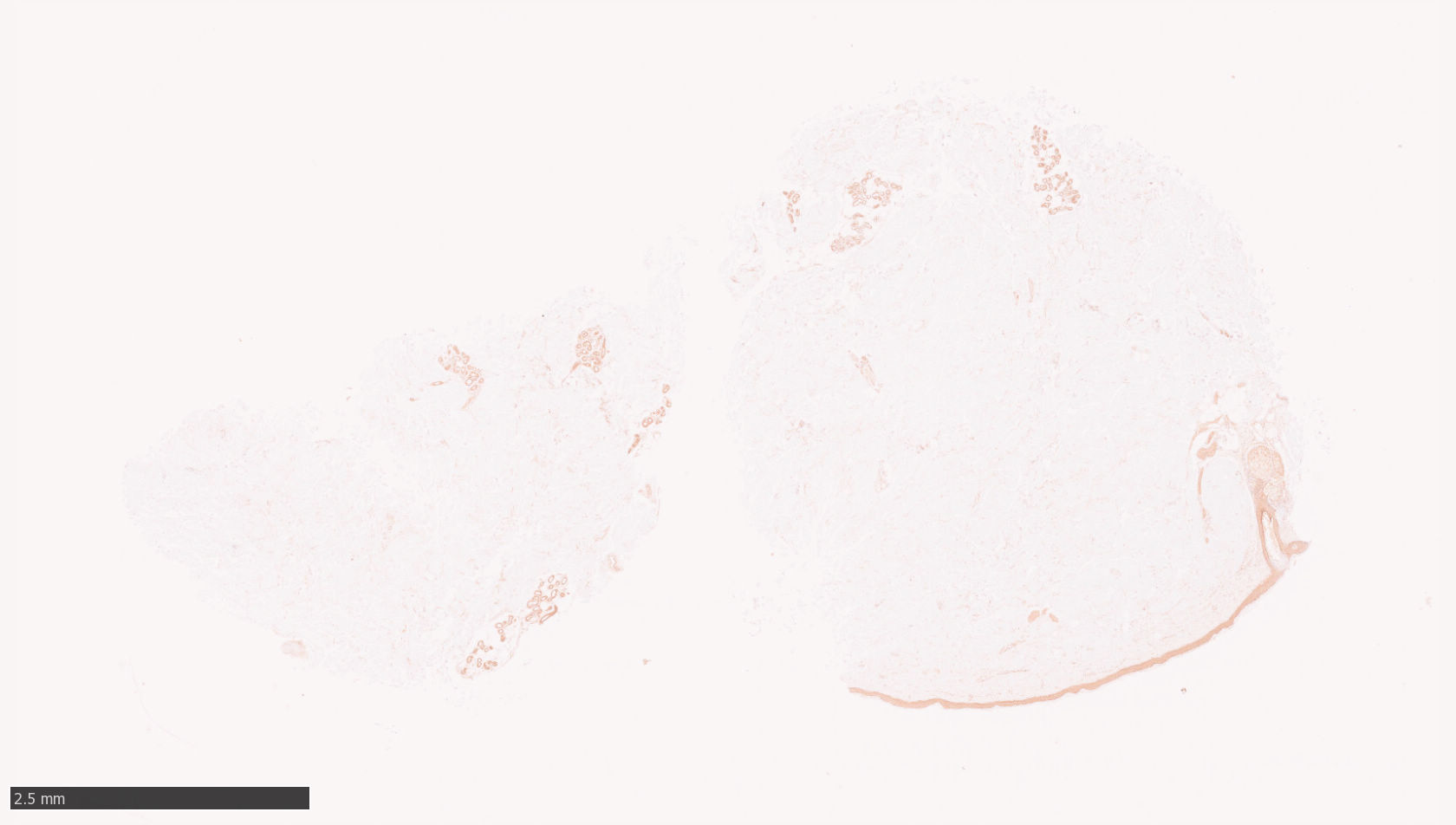


XH正47_NLRP1


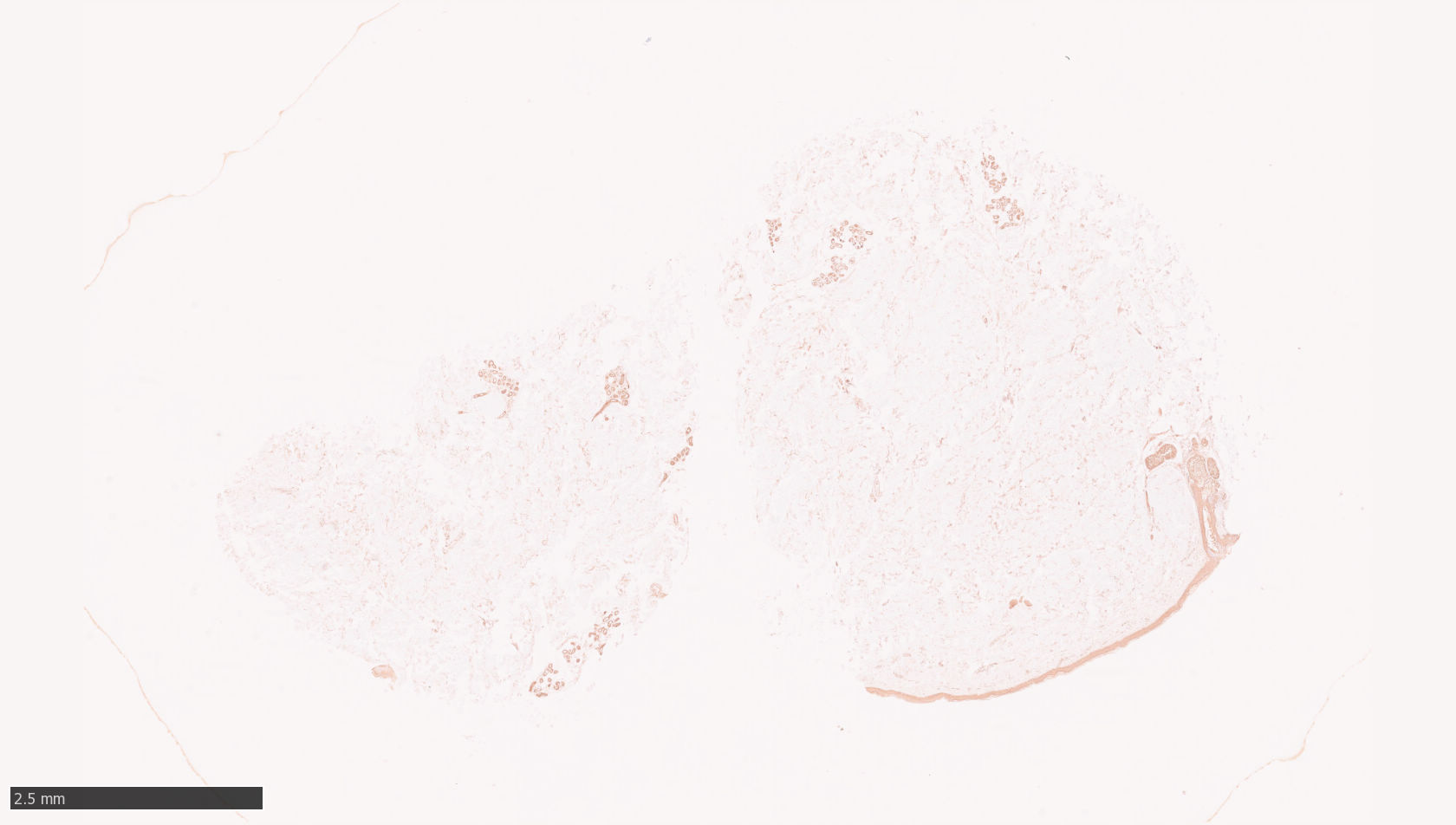


IHC uncropped figure-2

20-1261_CD8
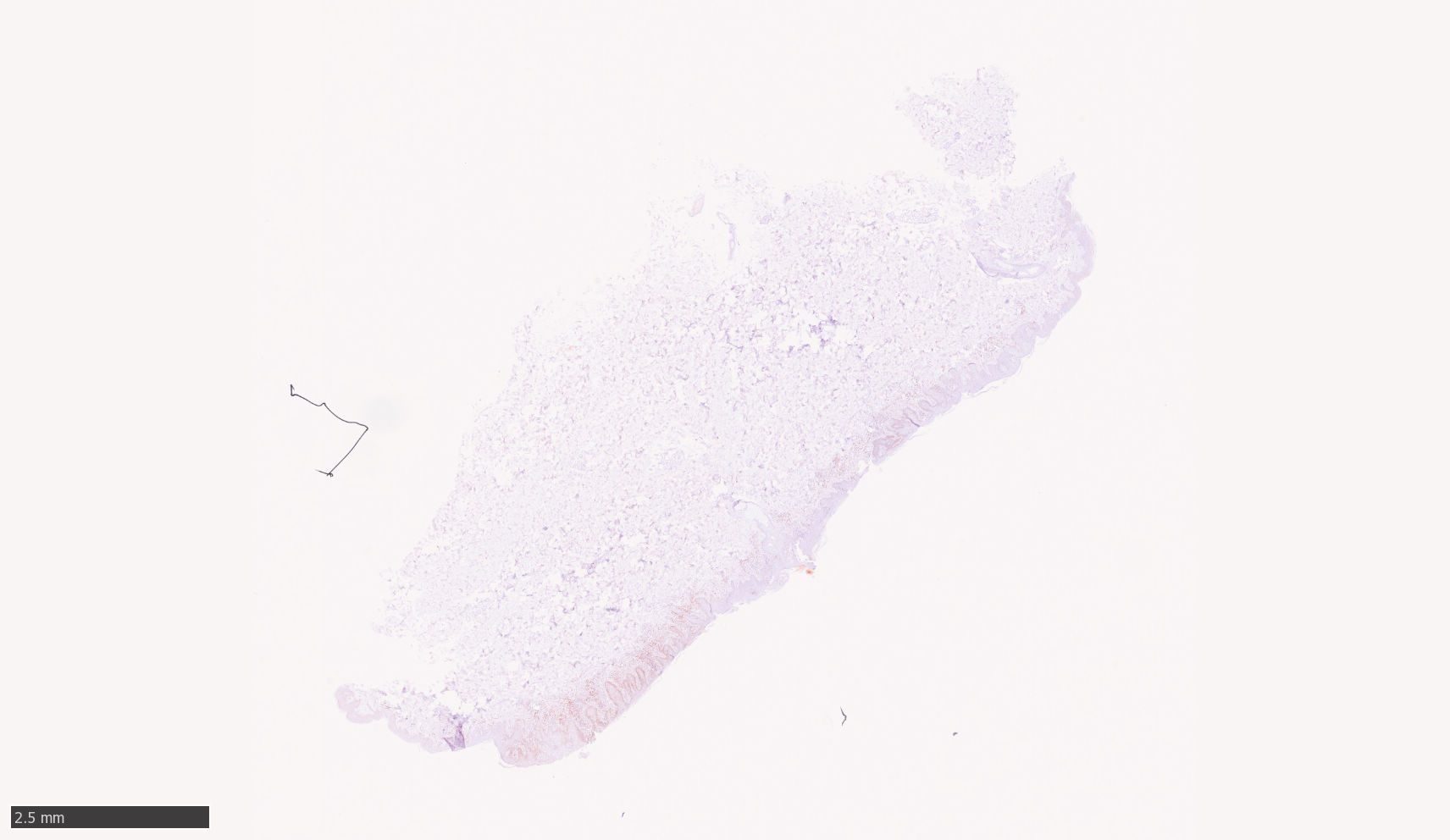


20-1261_CD57
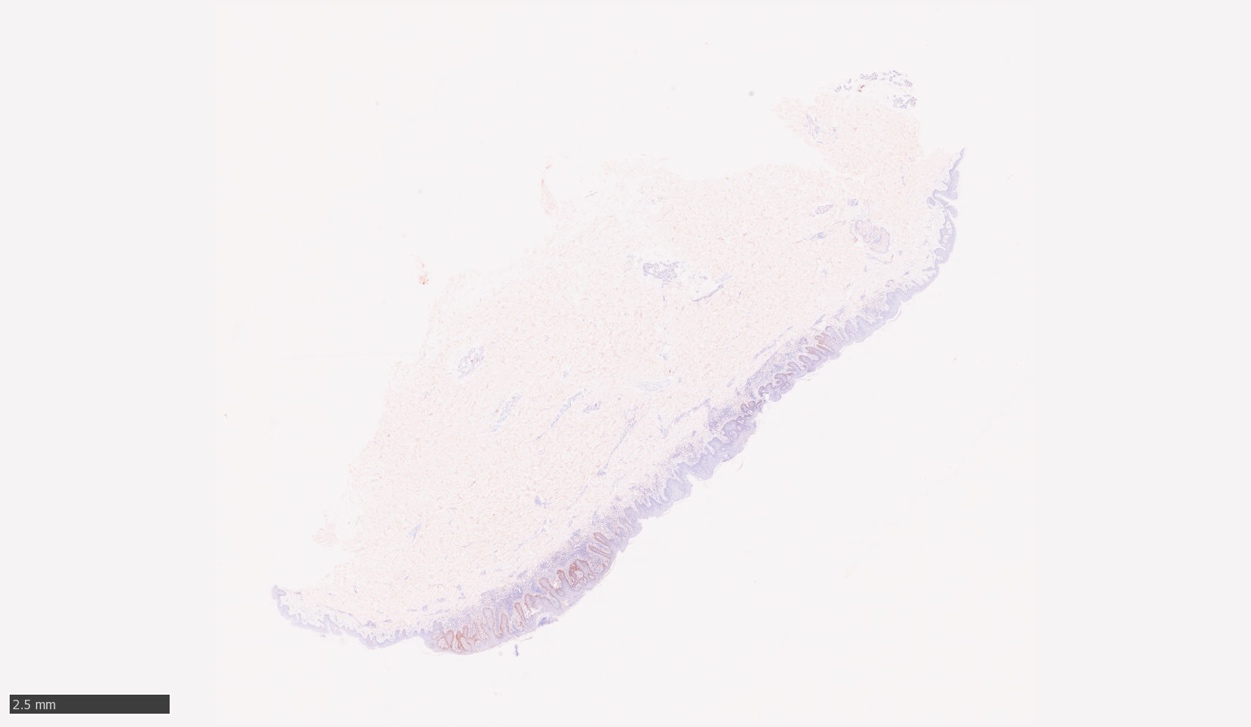


20-1261_CHMP4A
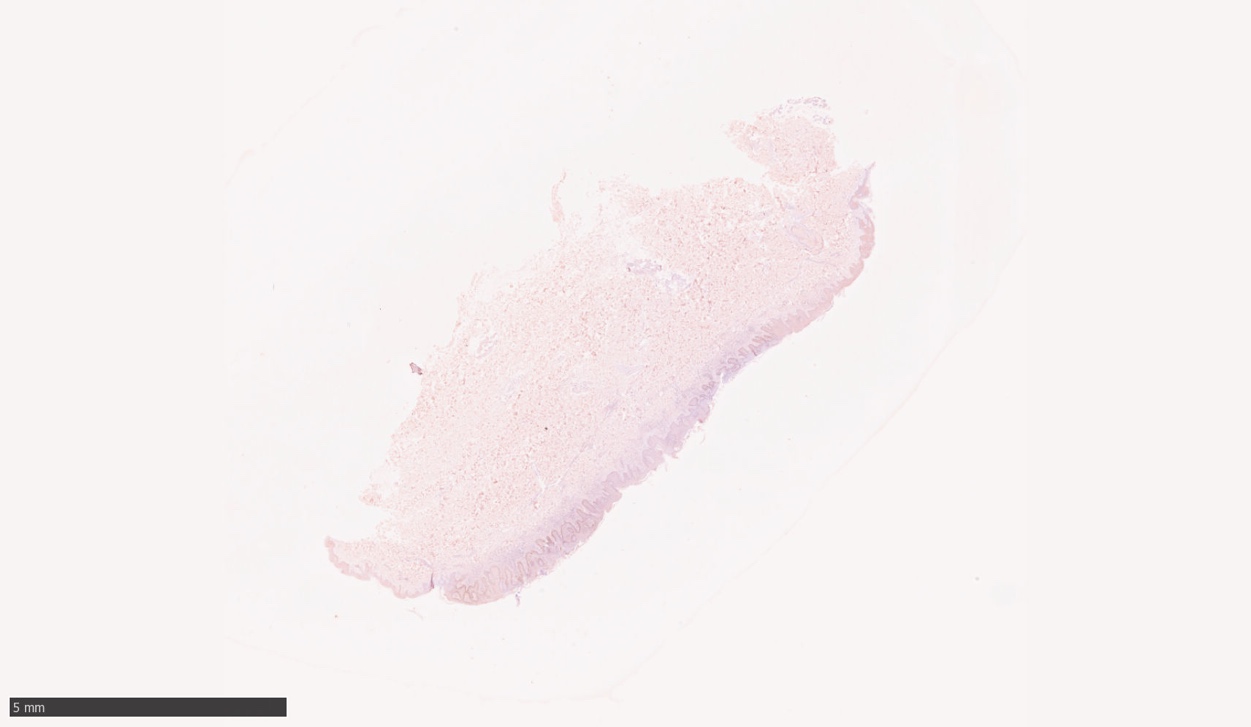
 20-1261_GSDMB
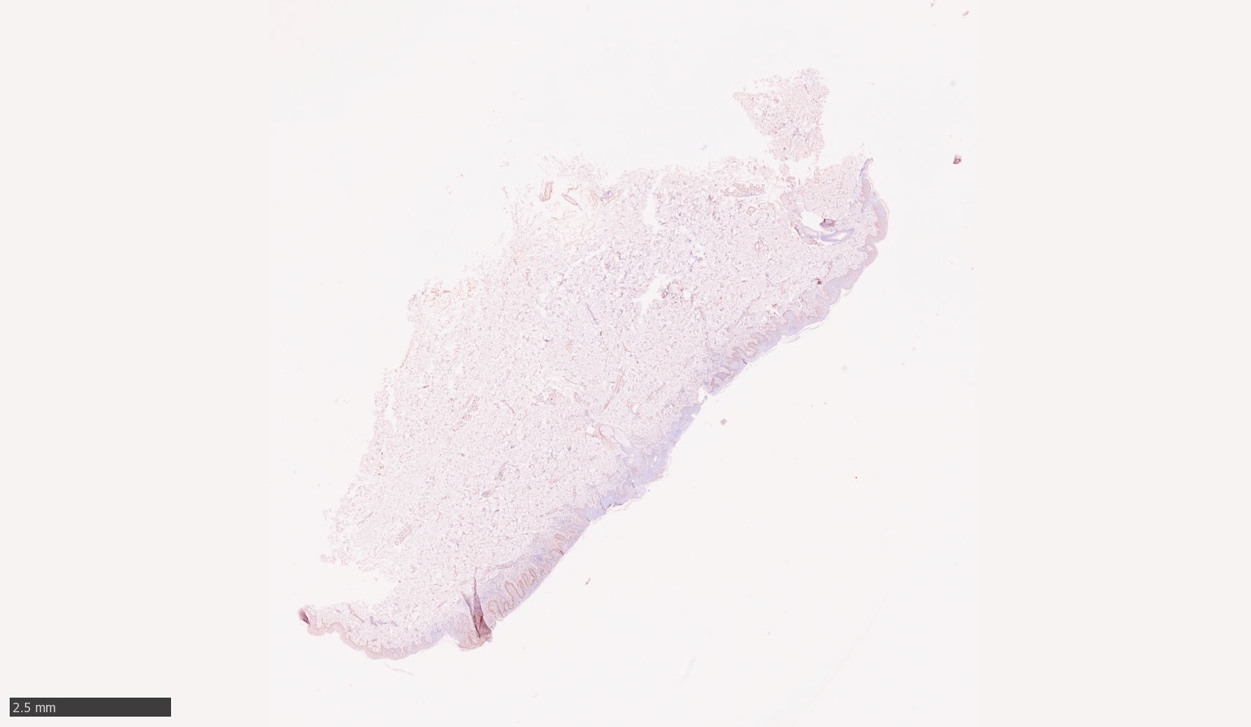


20-1261_GZMA
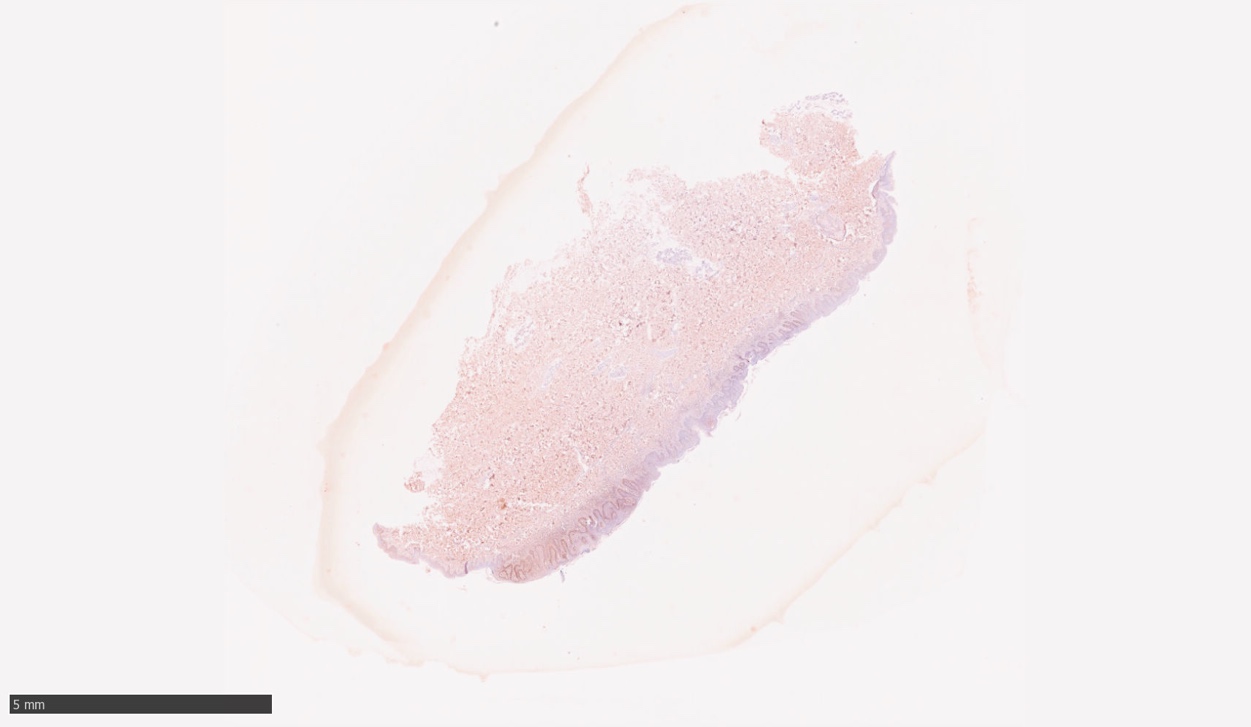
 20-1261_IL18
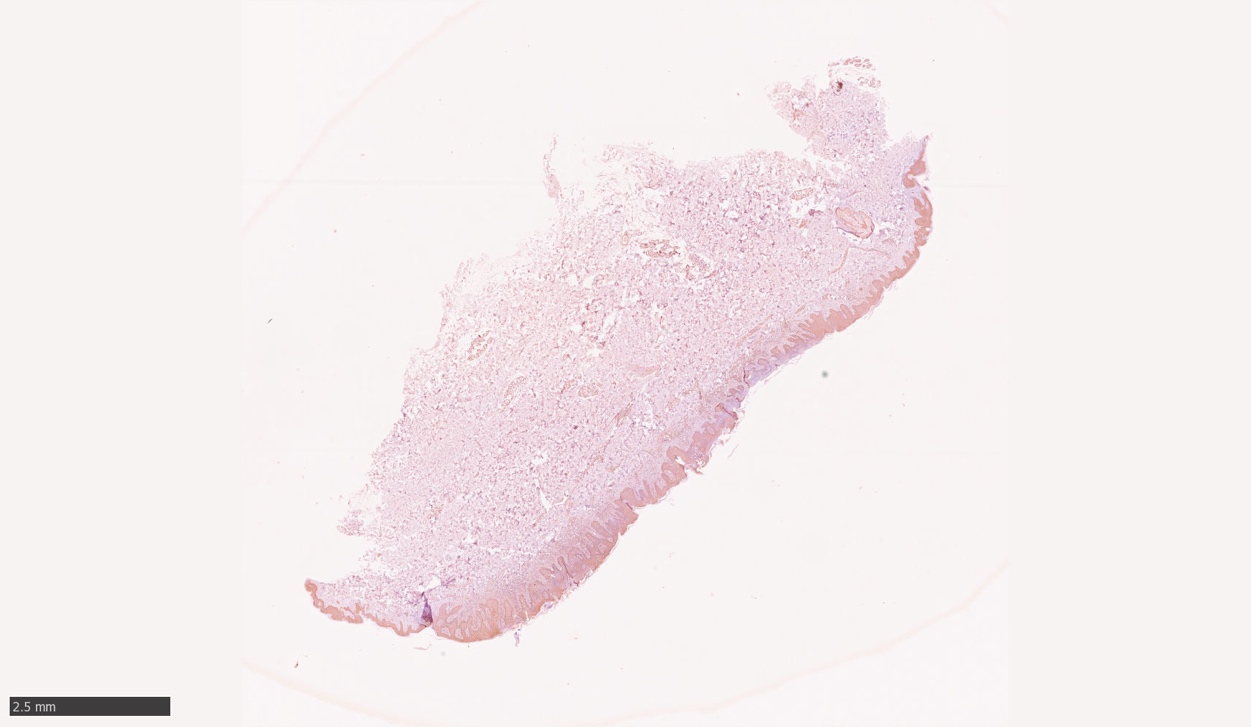


20-1261_NLRP1
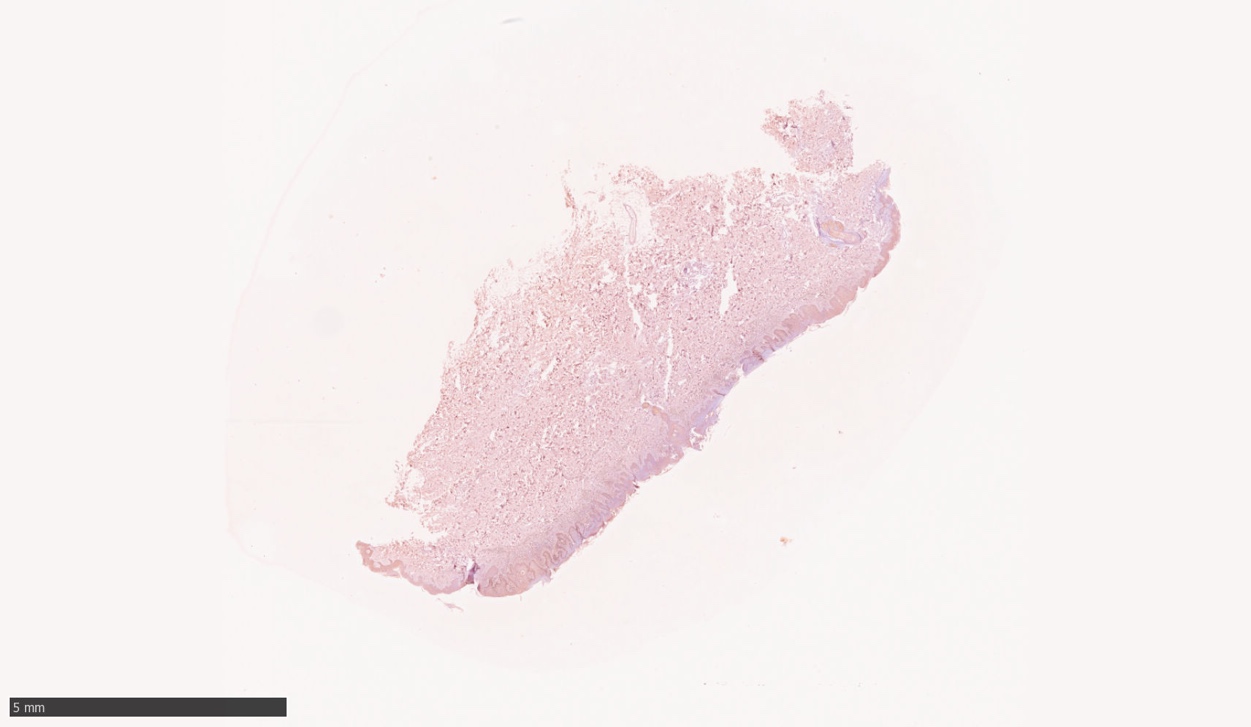
 20-7188_CD8
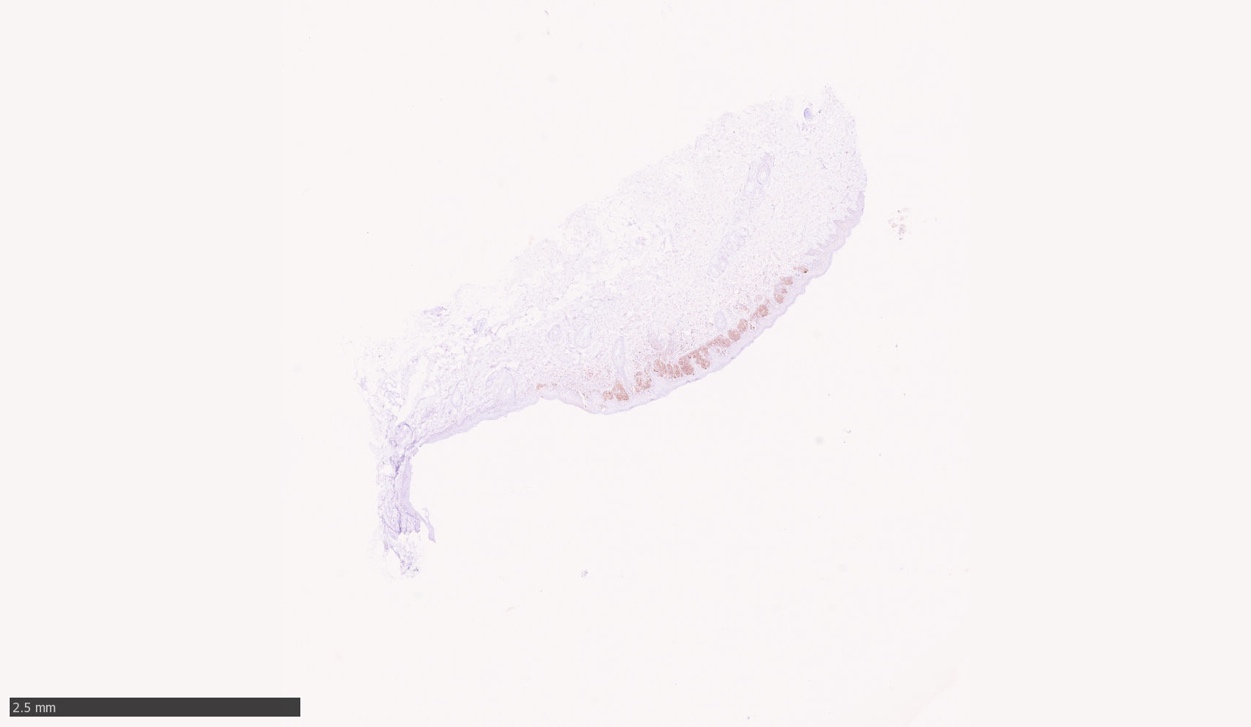


20-7188_CD57
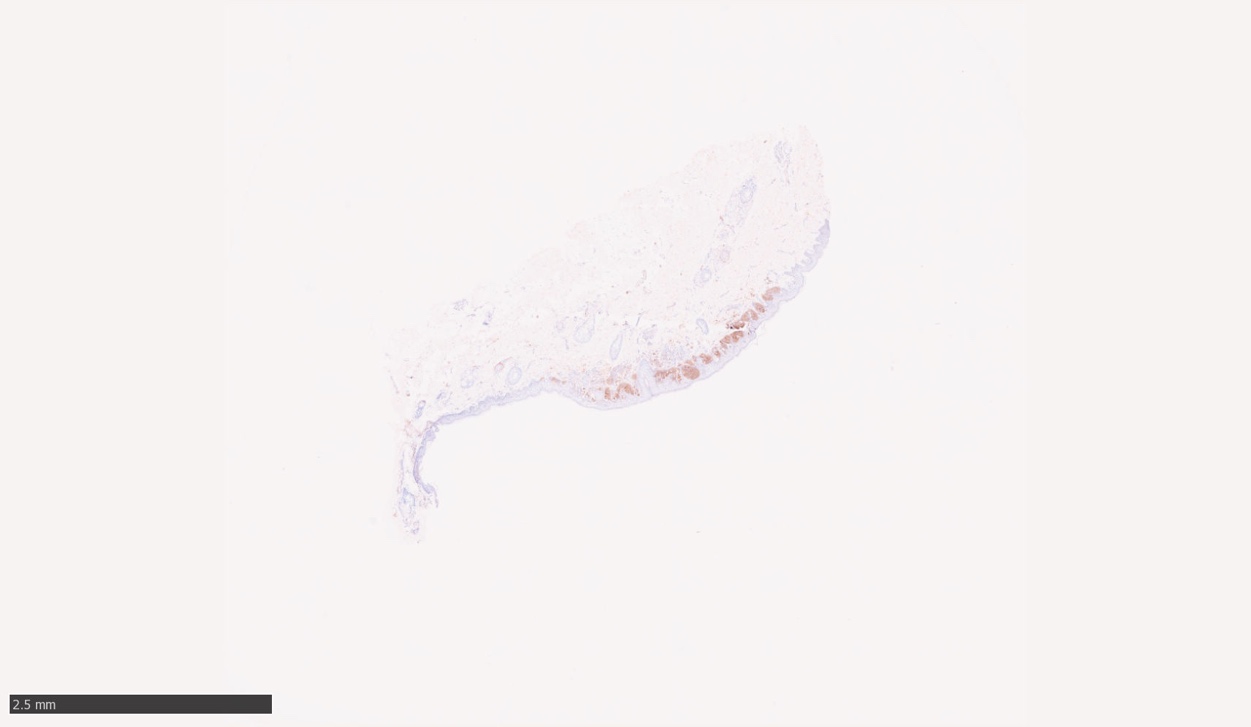
 20-7188_CHMP4A
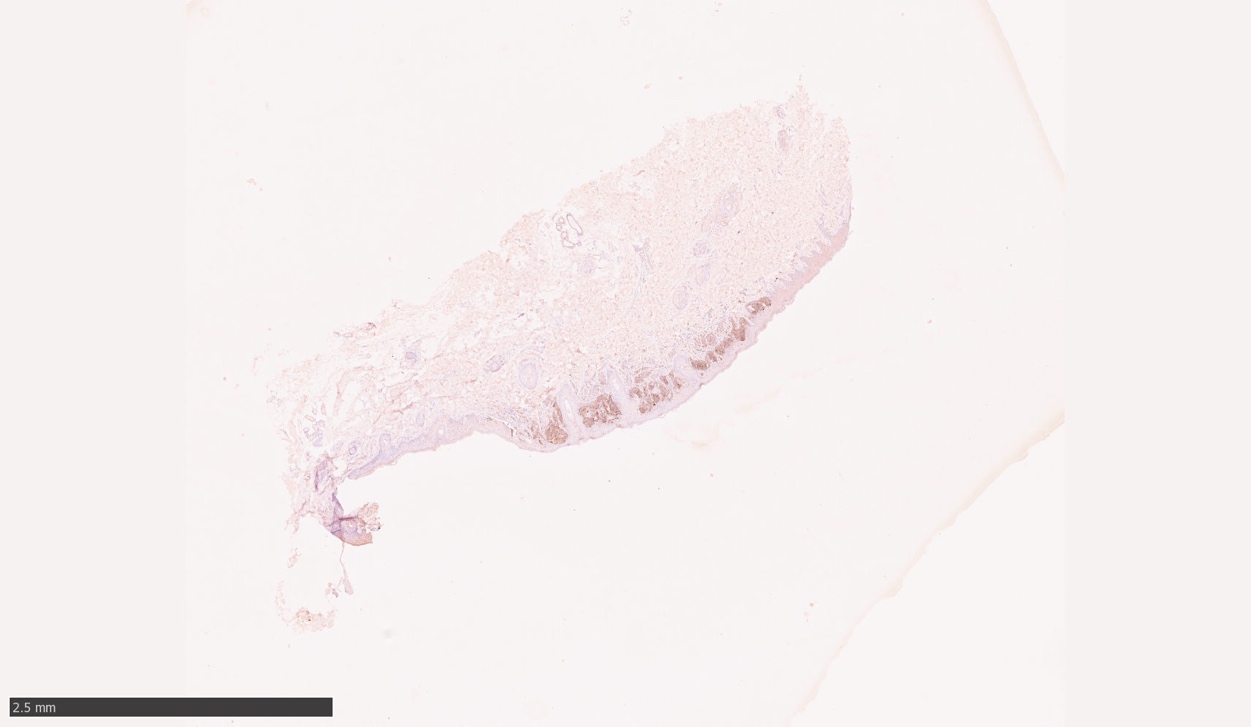


20-7188_GSDMB
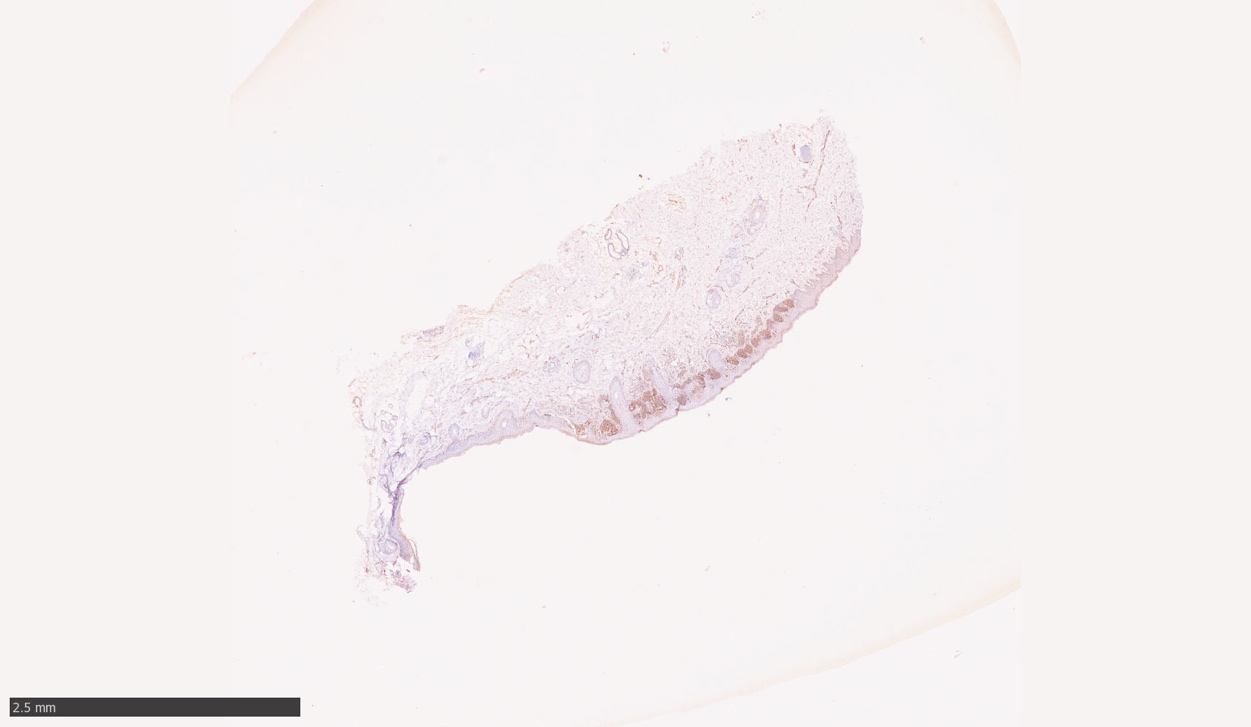
 20-7188_GZMA
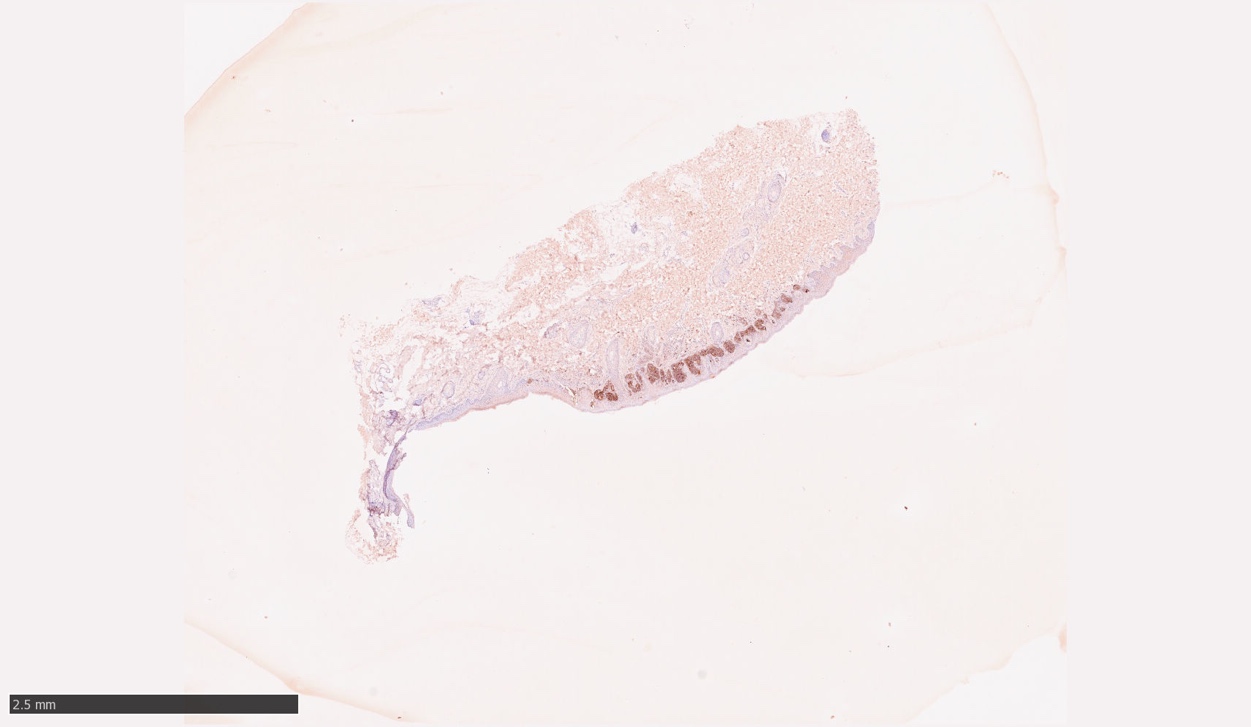


20-7188_IL18
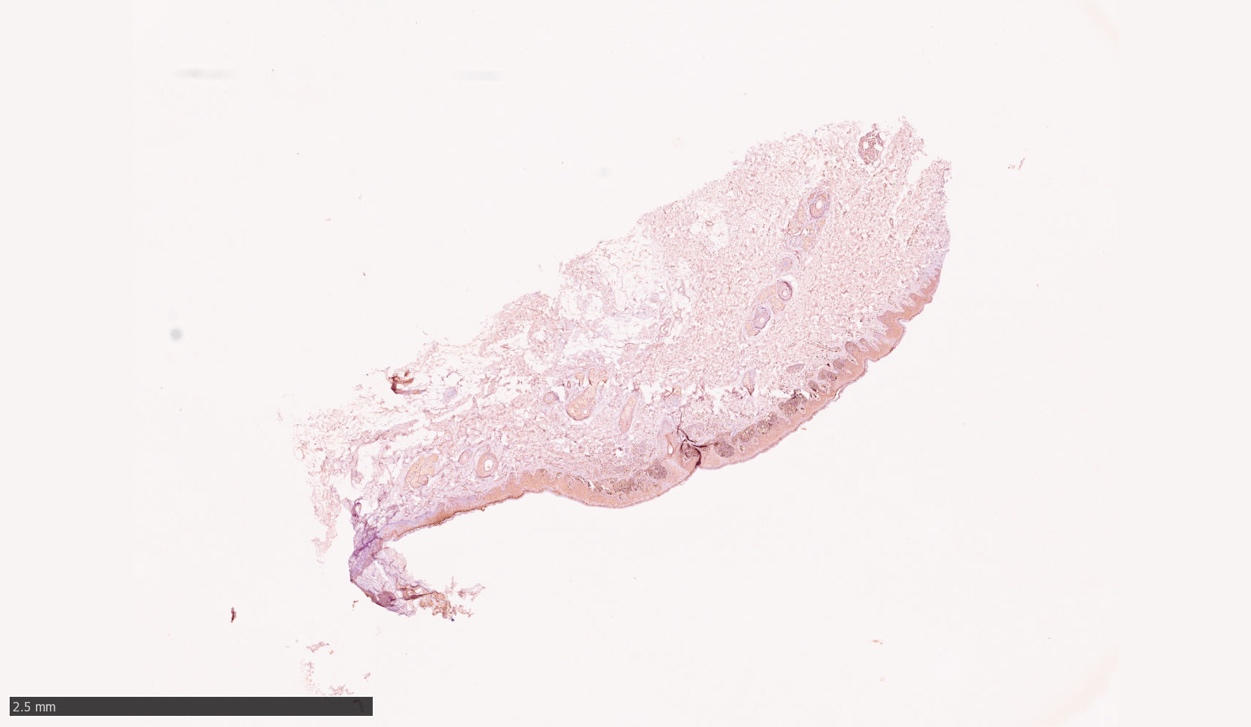
 20-7188_NLRP1
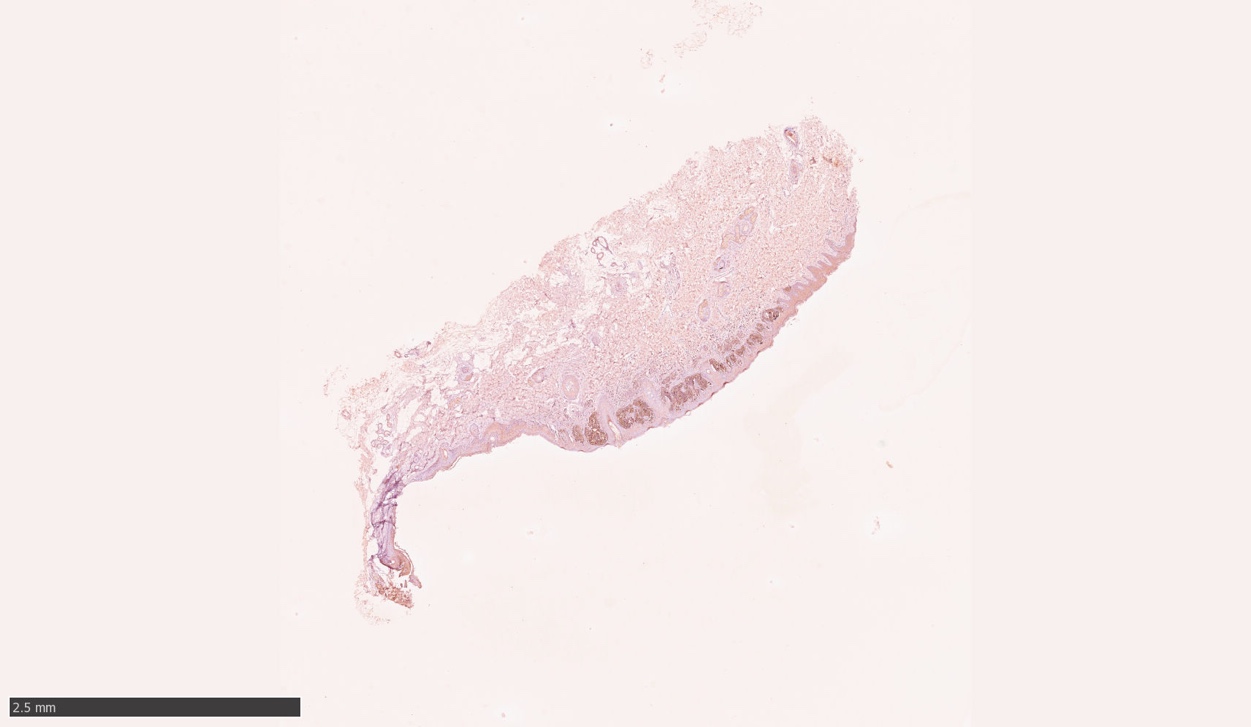


20-7503_CD8
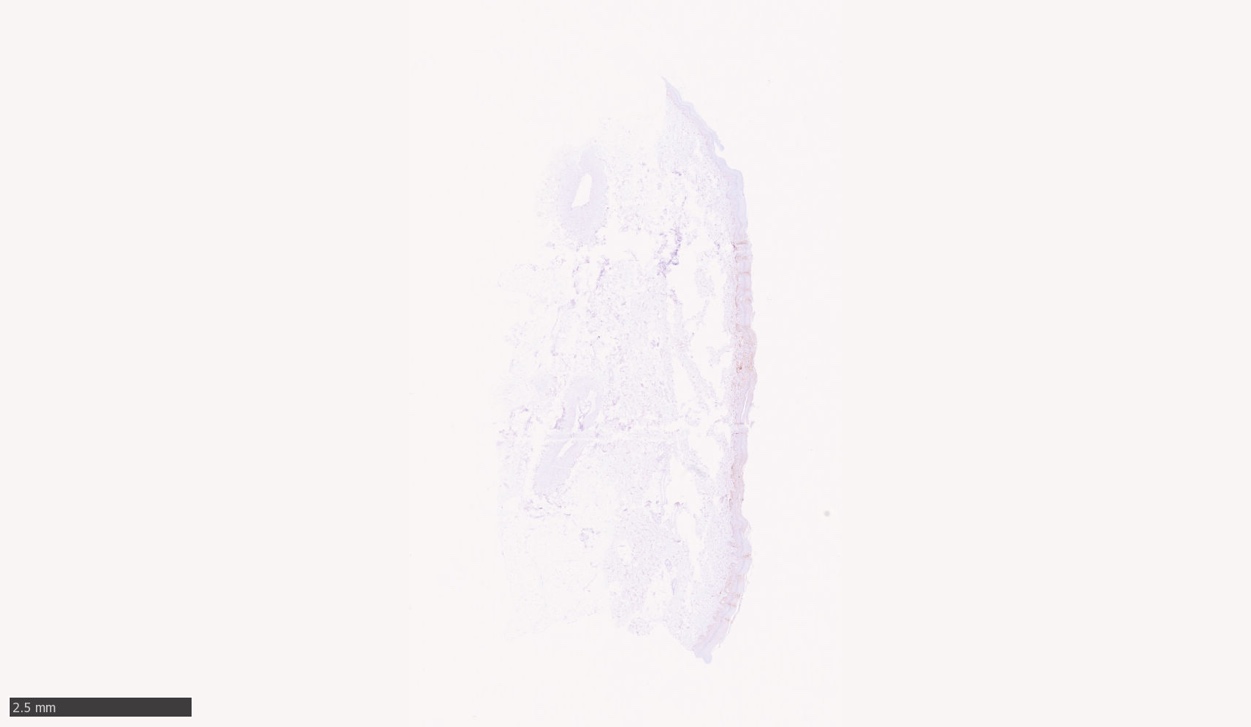


20-7503_CD57
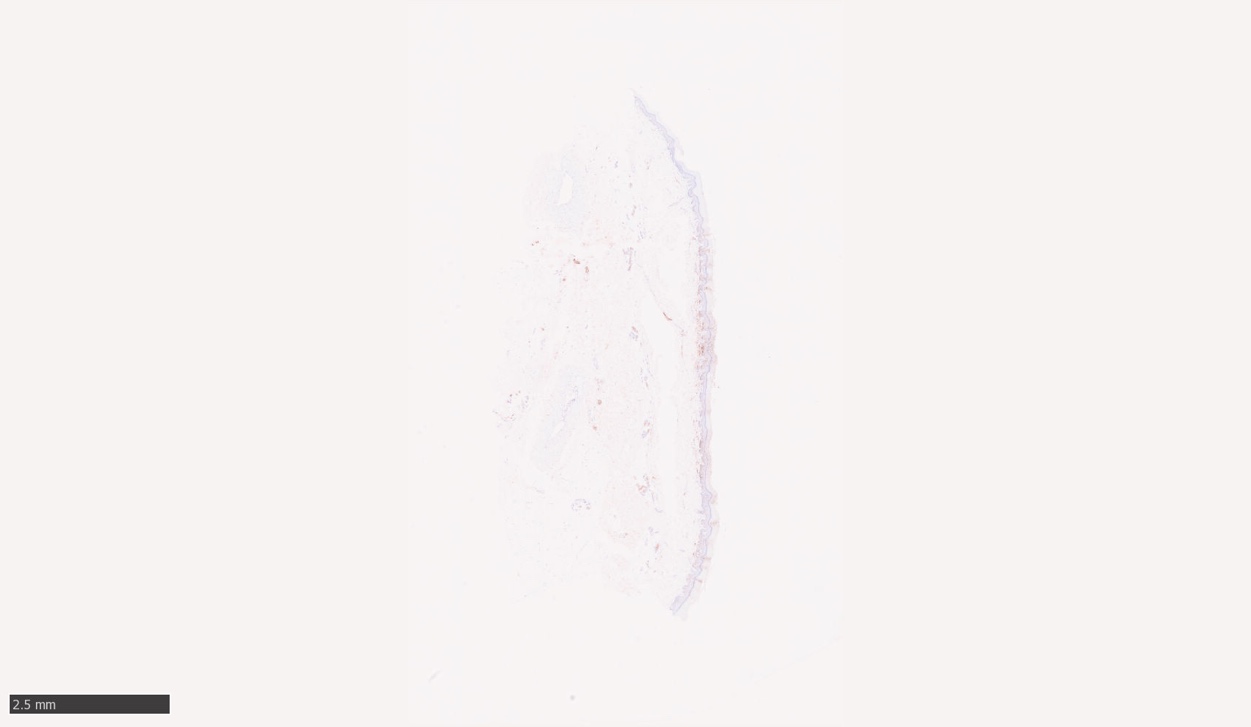


20-7503_CHMP4A
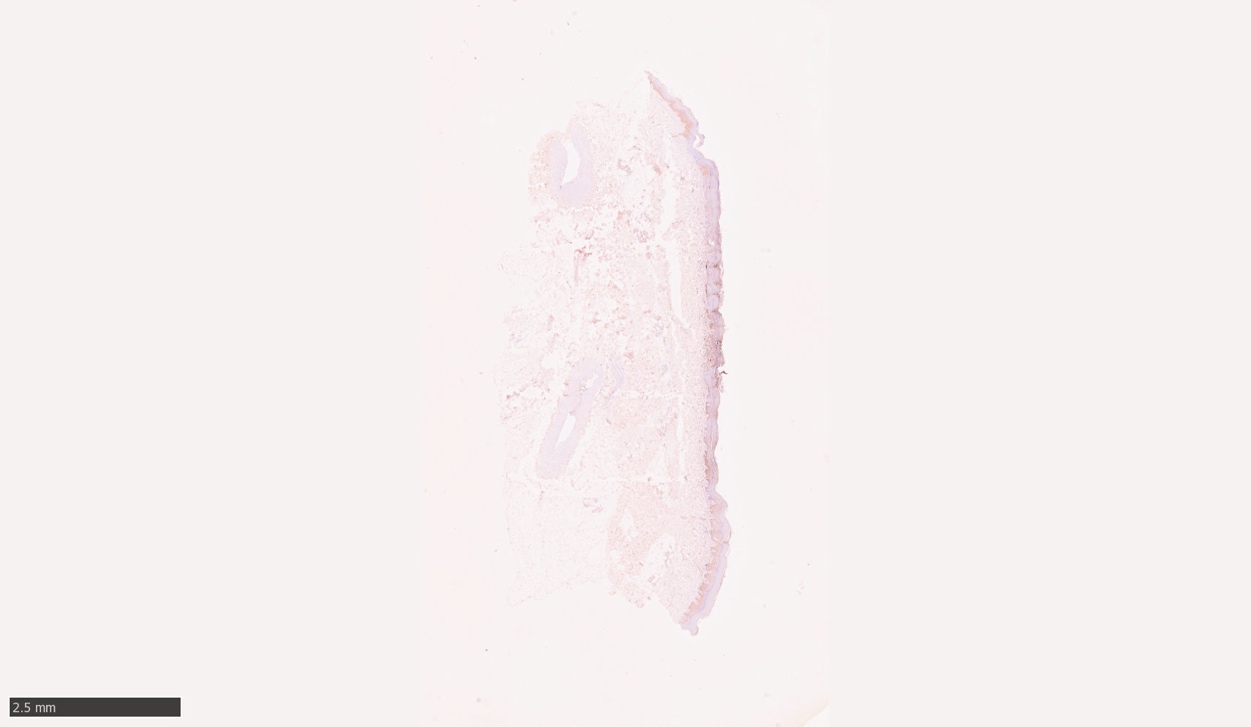


20-7503_GSDMB
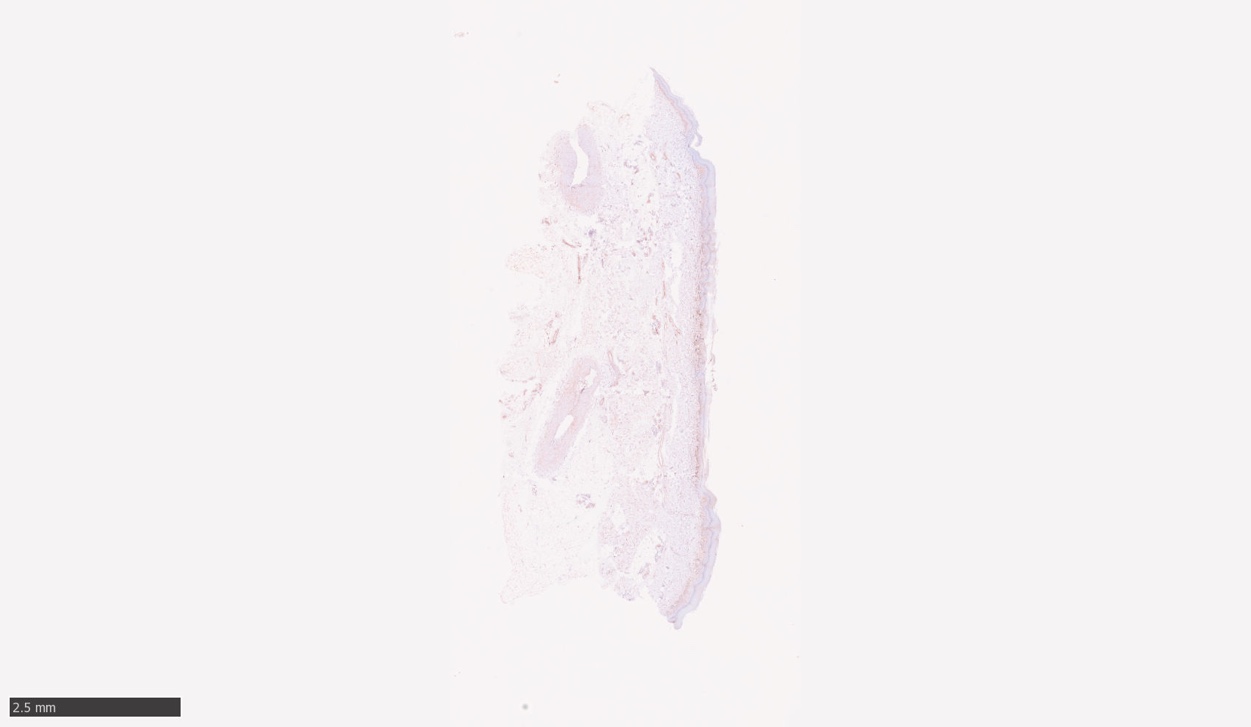


20-7503_GZMA
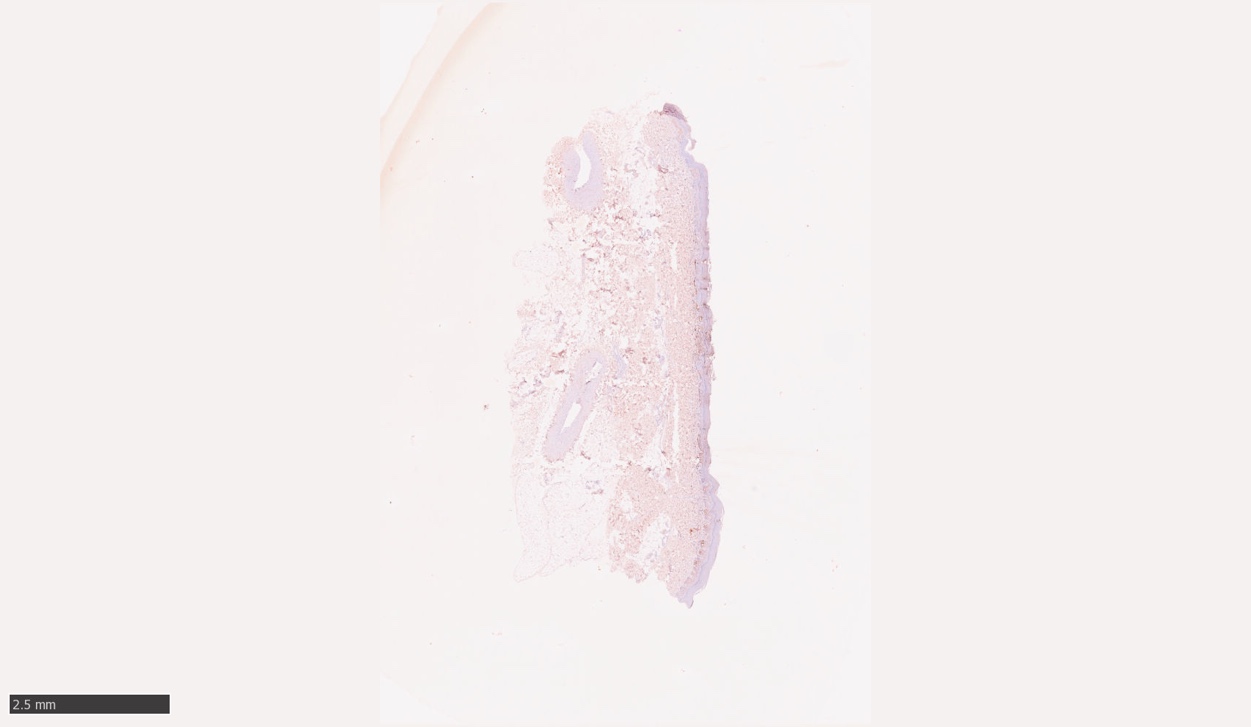


20-7503_IL18
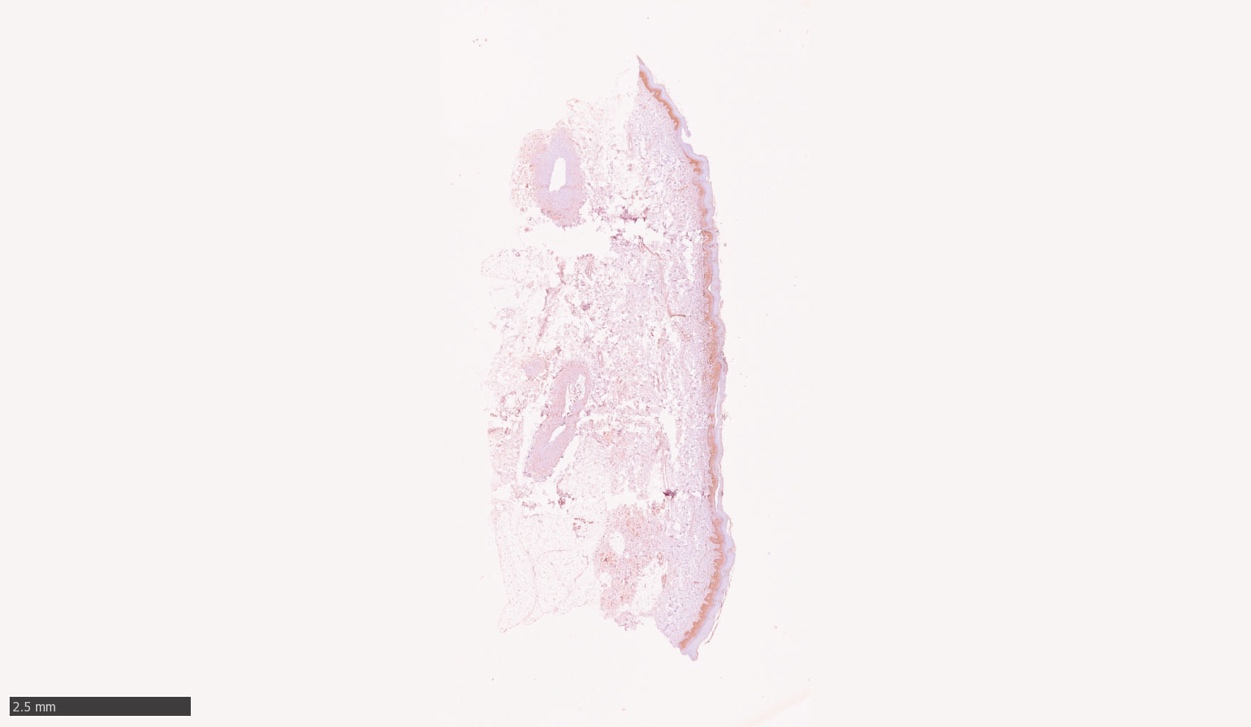


20-7503_NLRP1
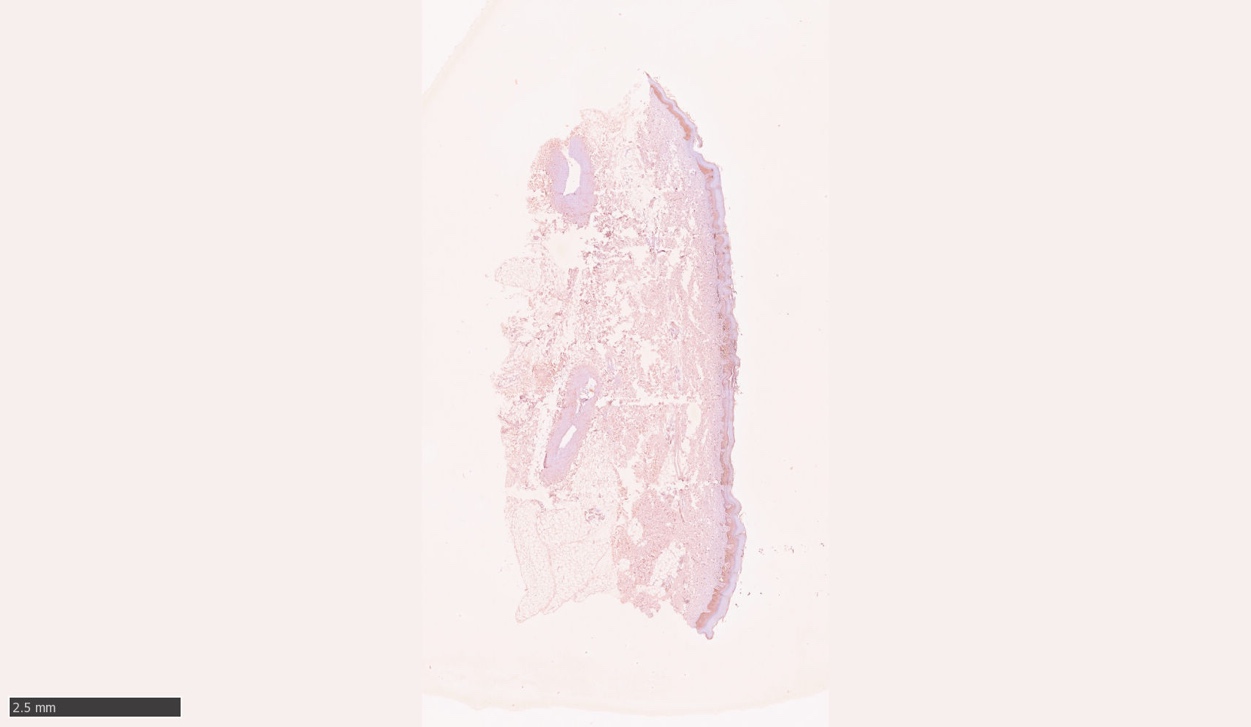


21-4582_CD8
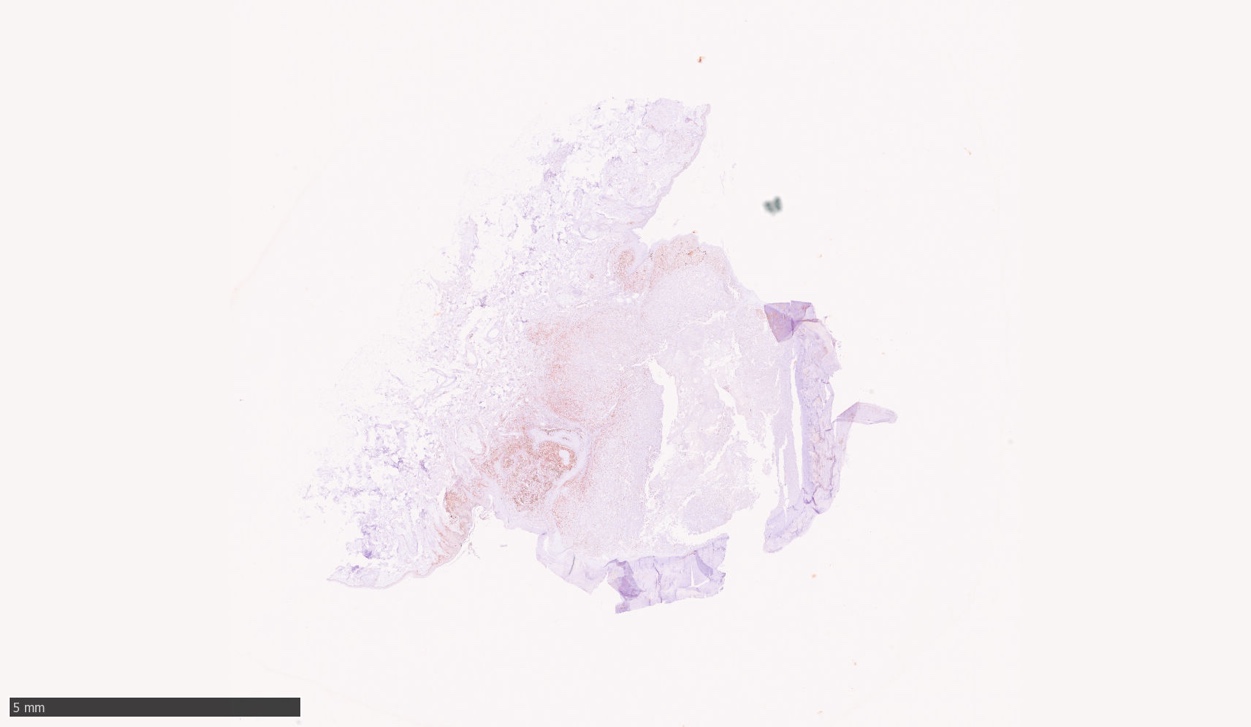


21-4582_CD57
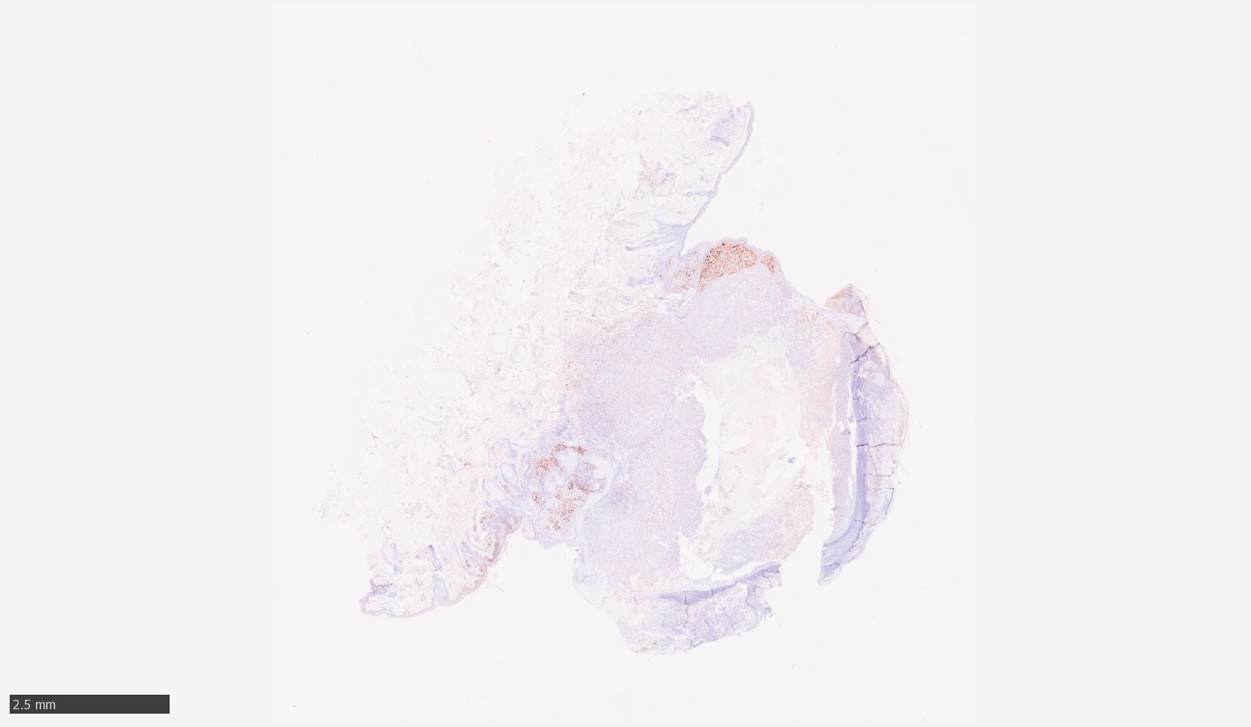


21-4582_CHMP4A
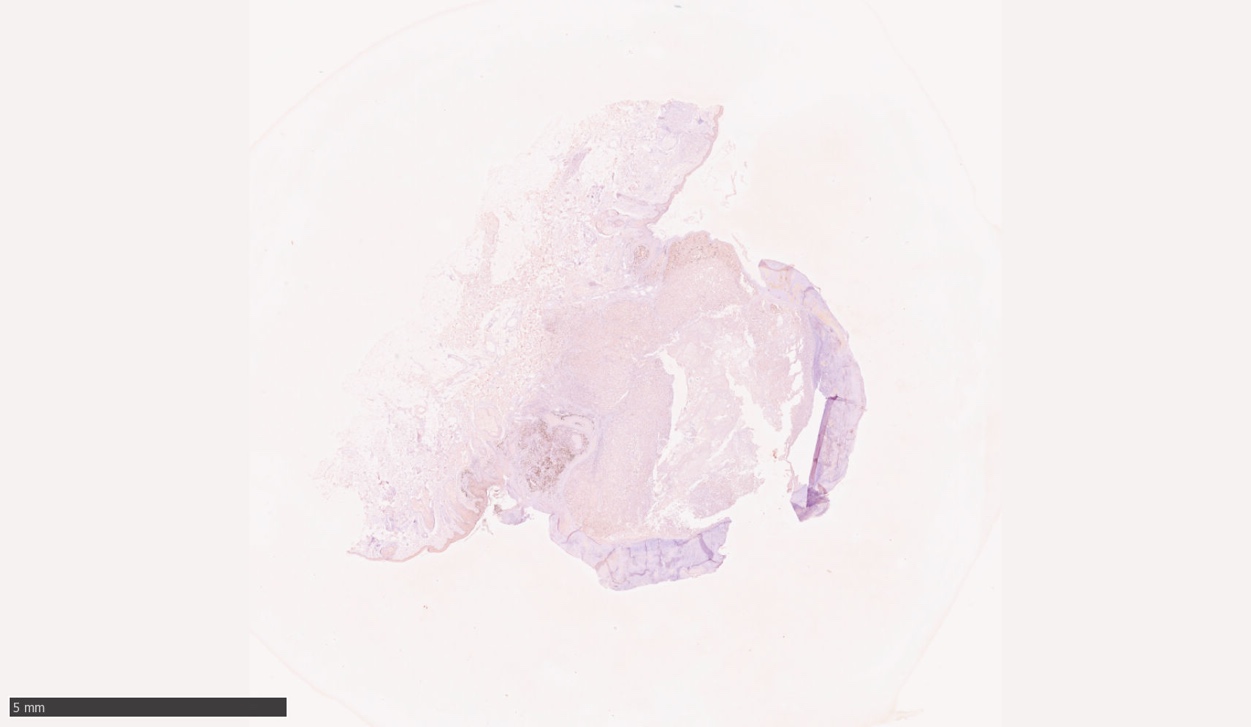


21-4582_GSDMB
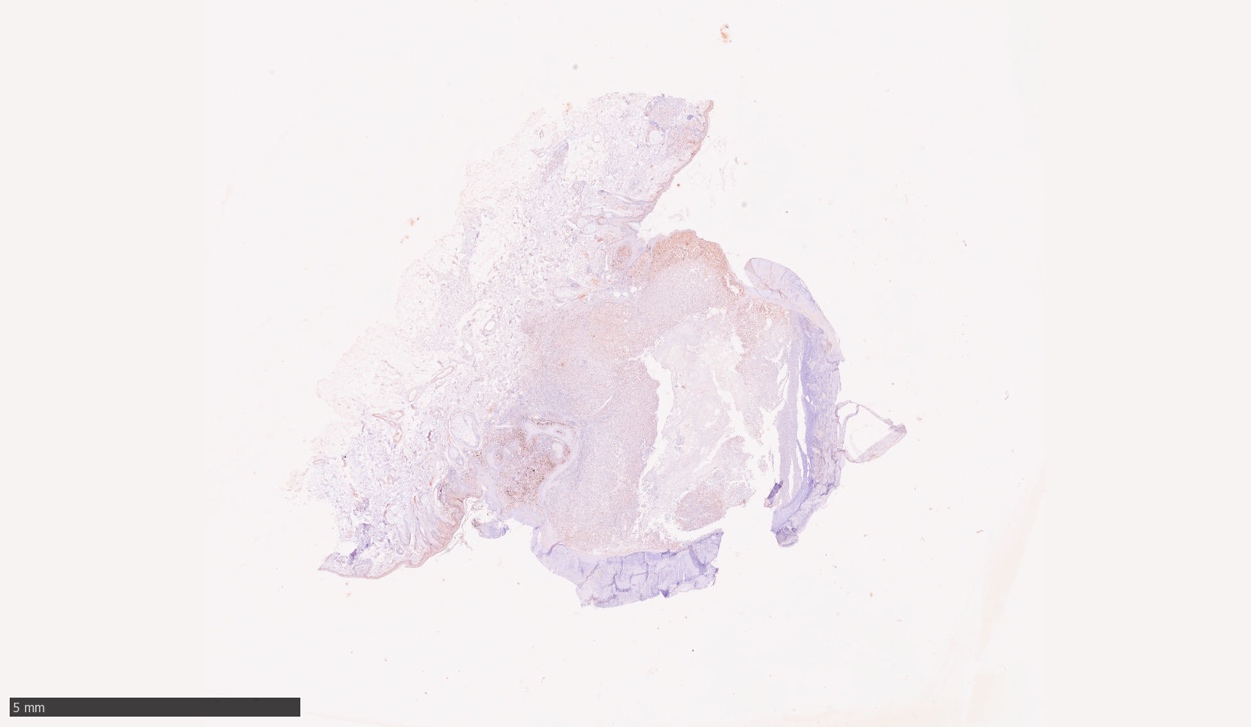


21-4582_GZMA
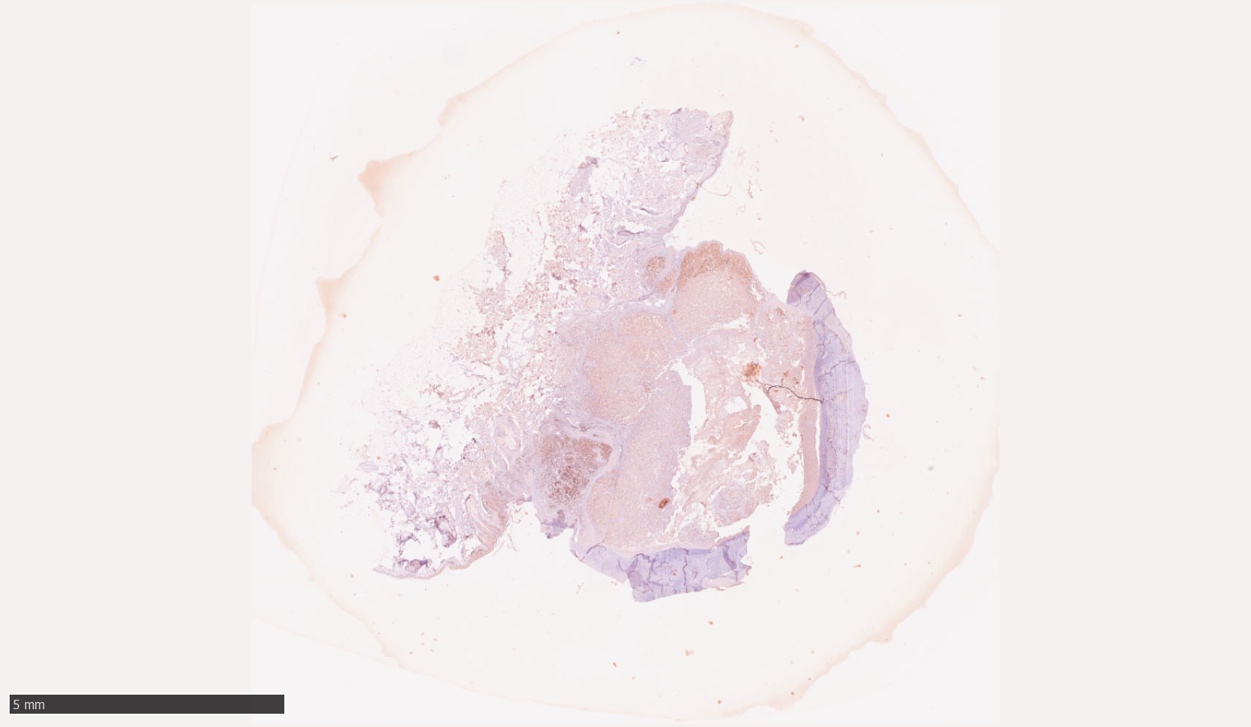


21-4582_IL18
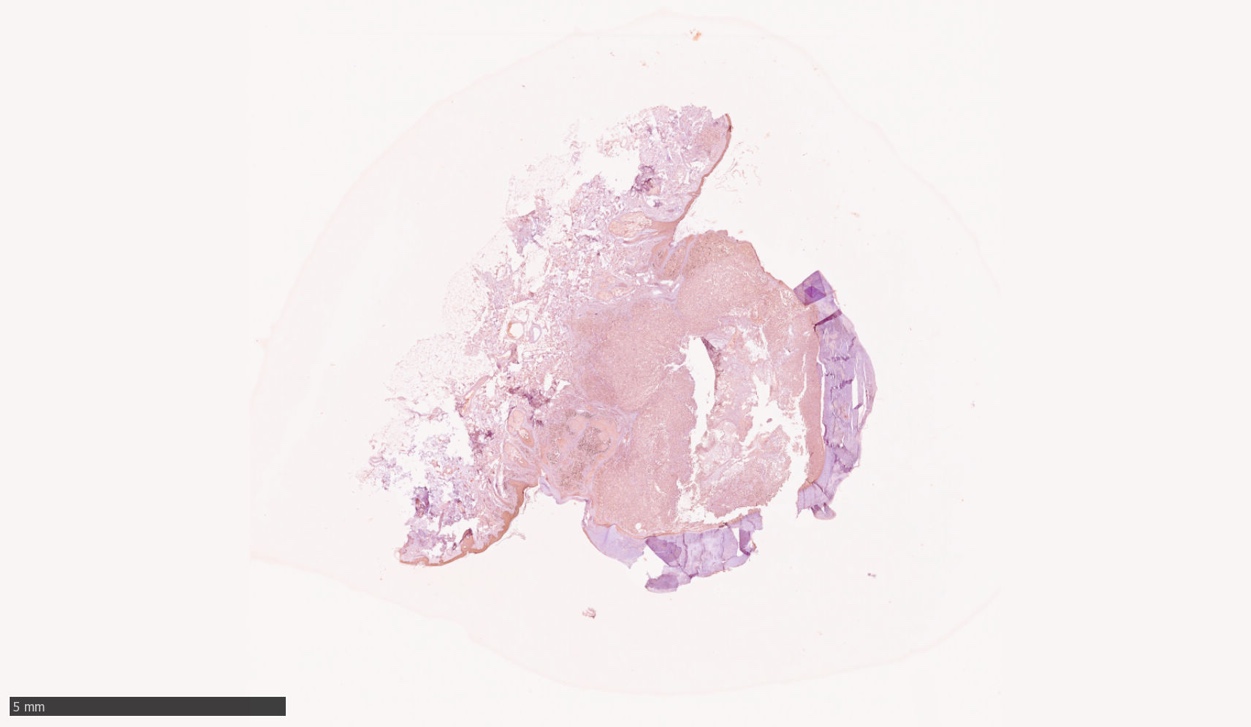
 21-4582_NLRP1
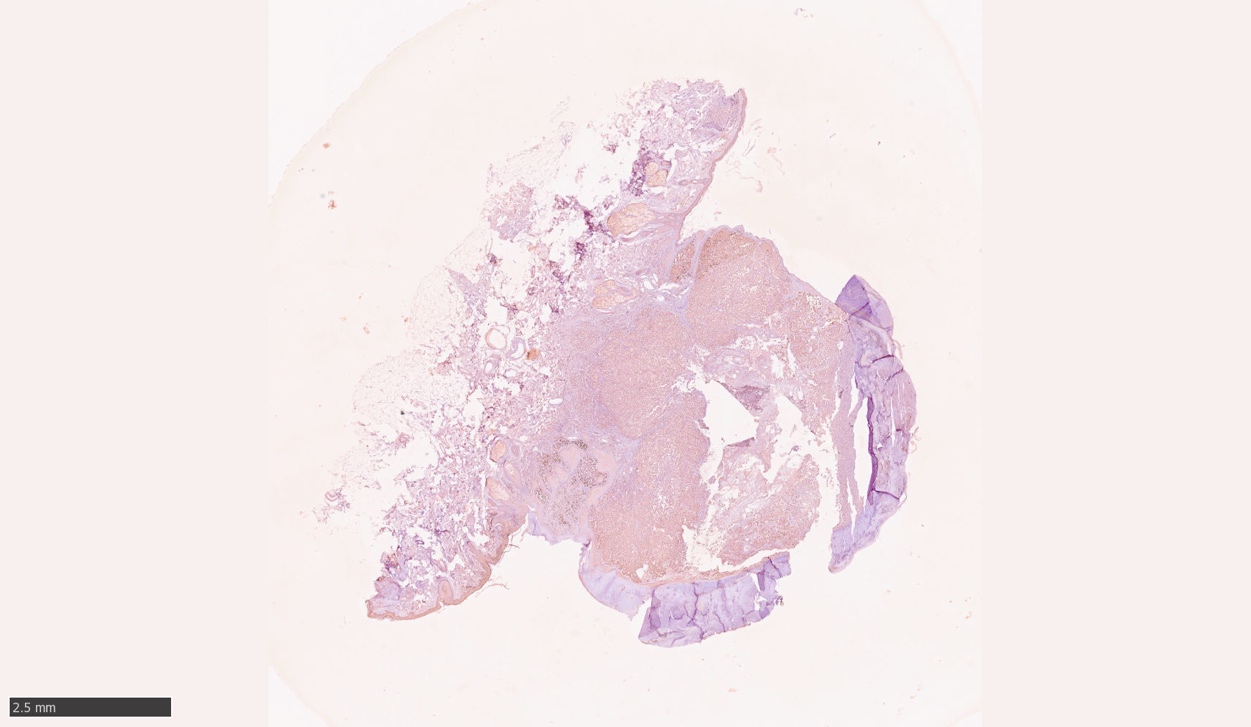


22-7459_CD8
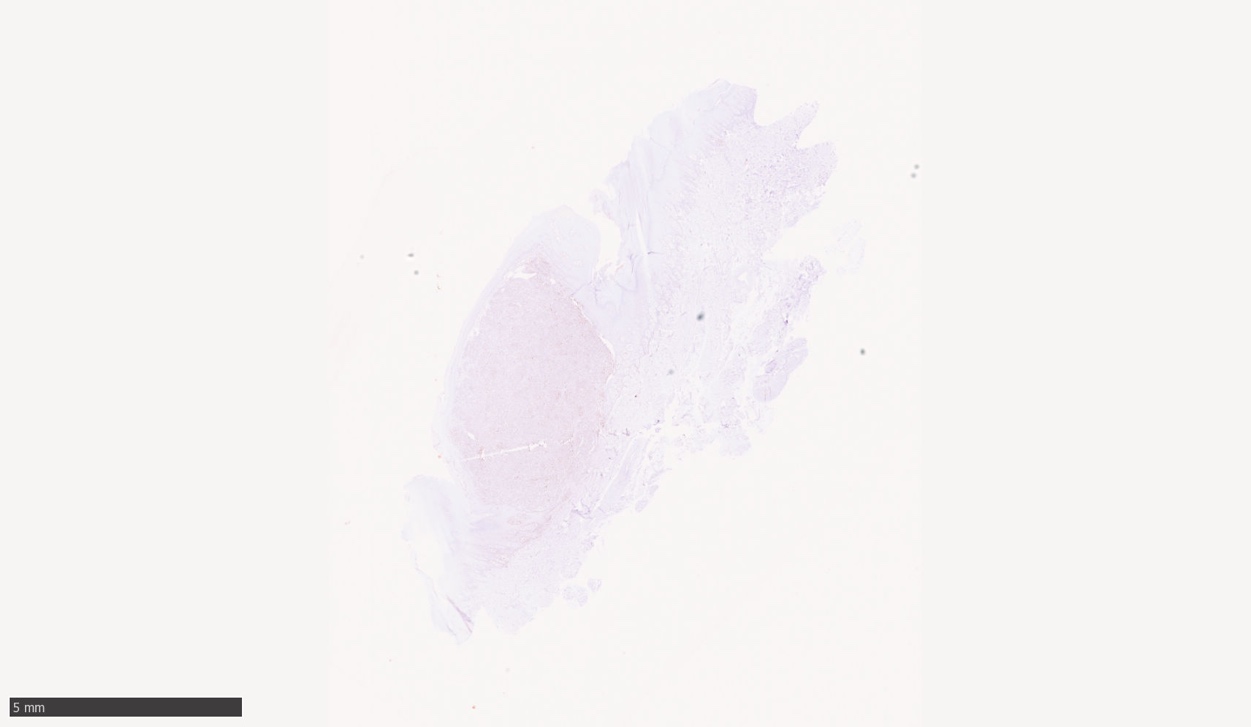


22-7459_CD57
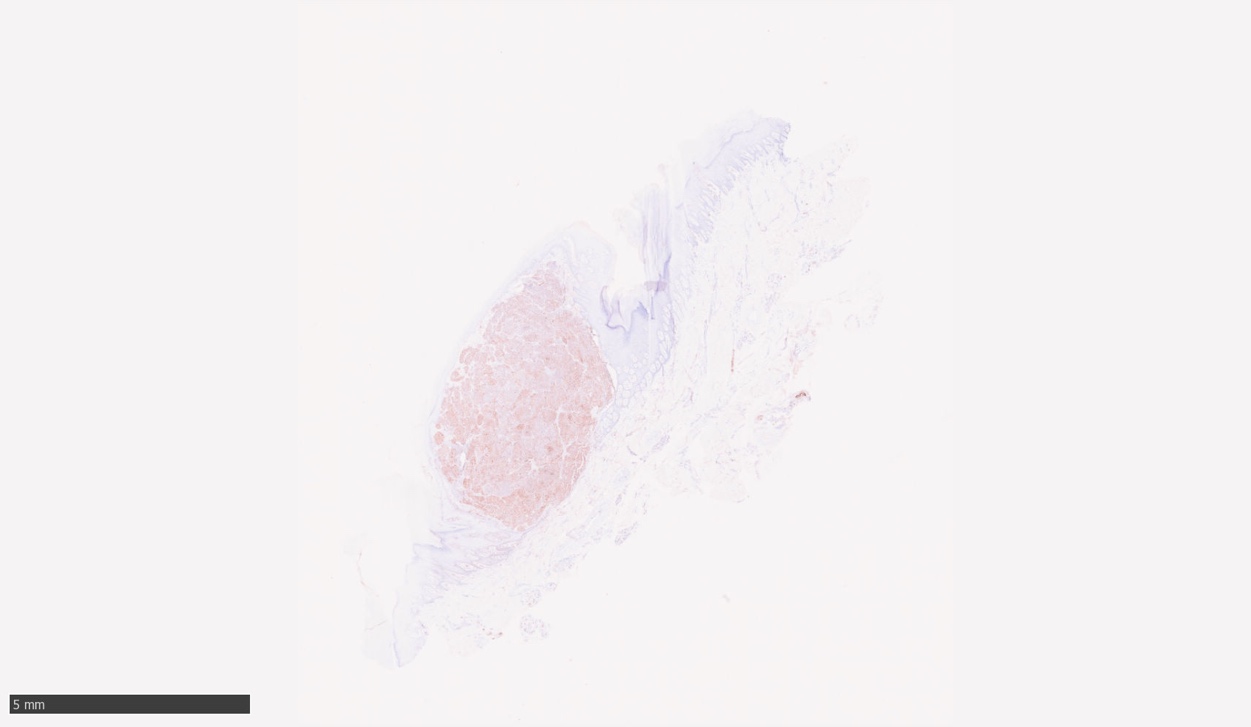


22-7459_CHMP4A
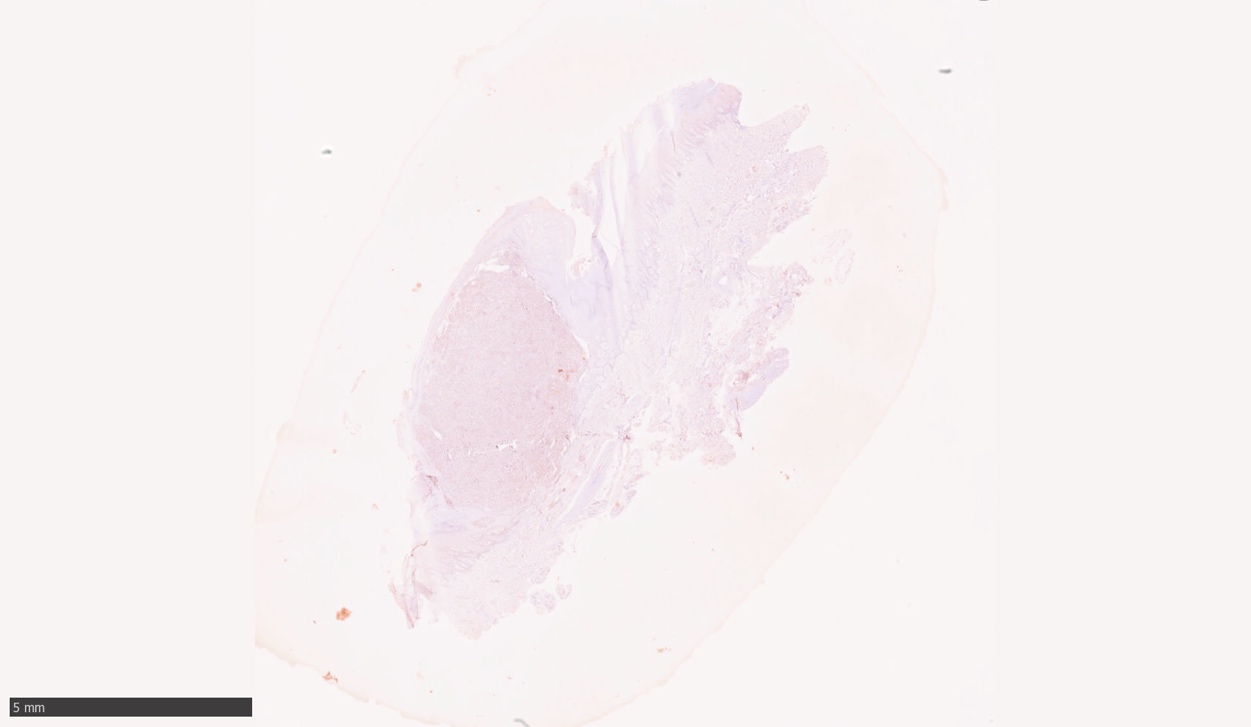


22-7459_GSDMB
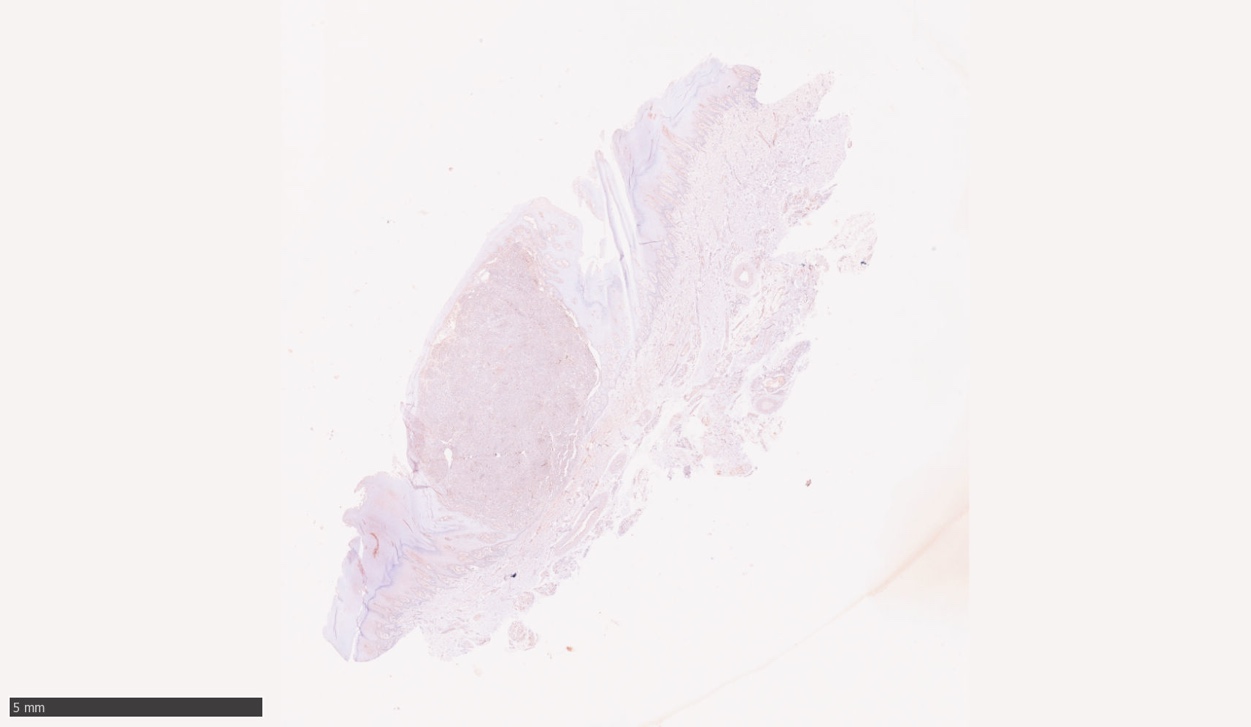


22-7459_GZMA
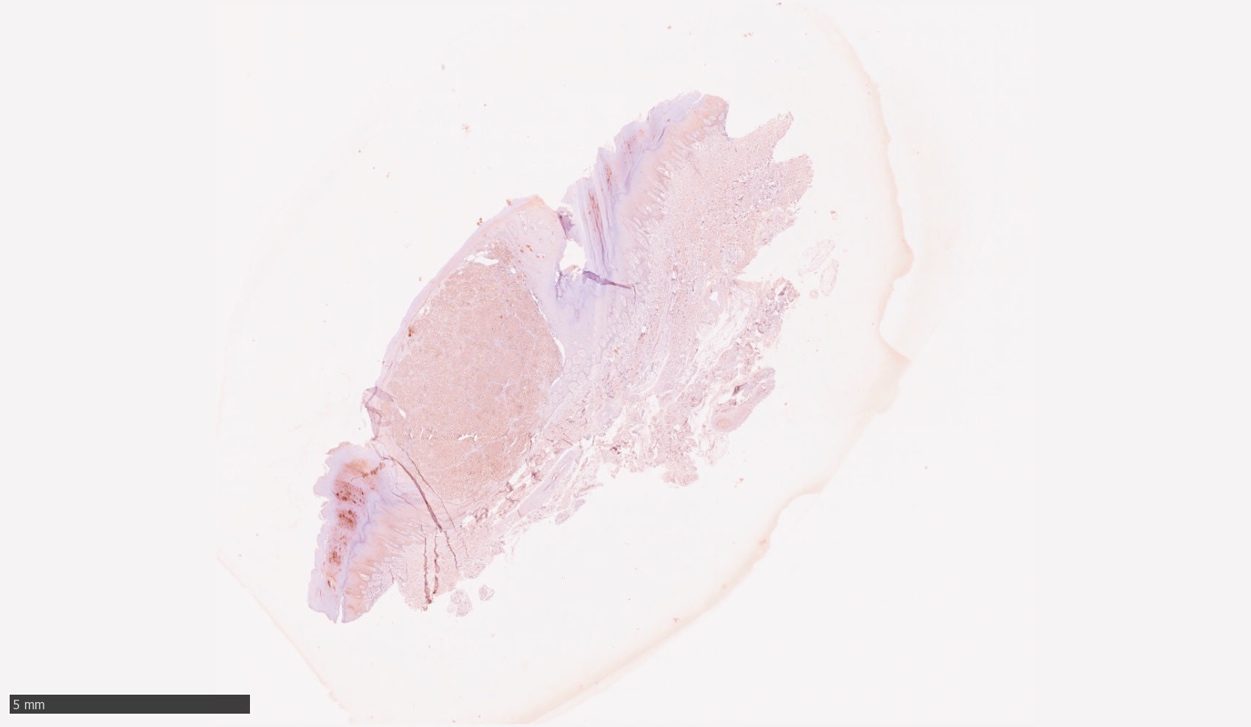


22-7459_IL18
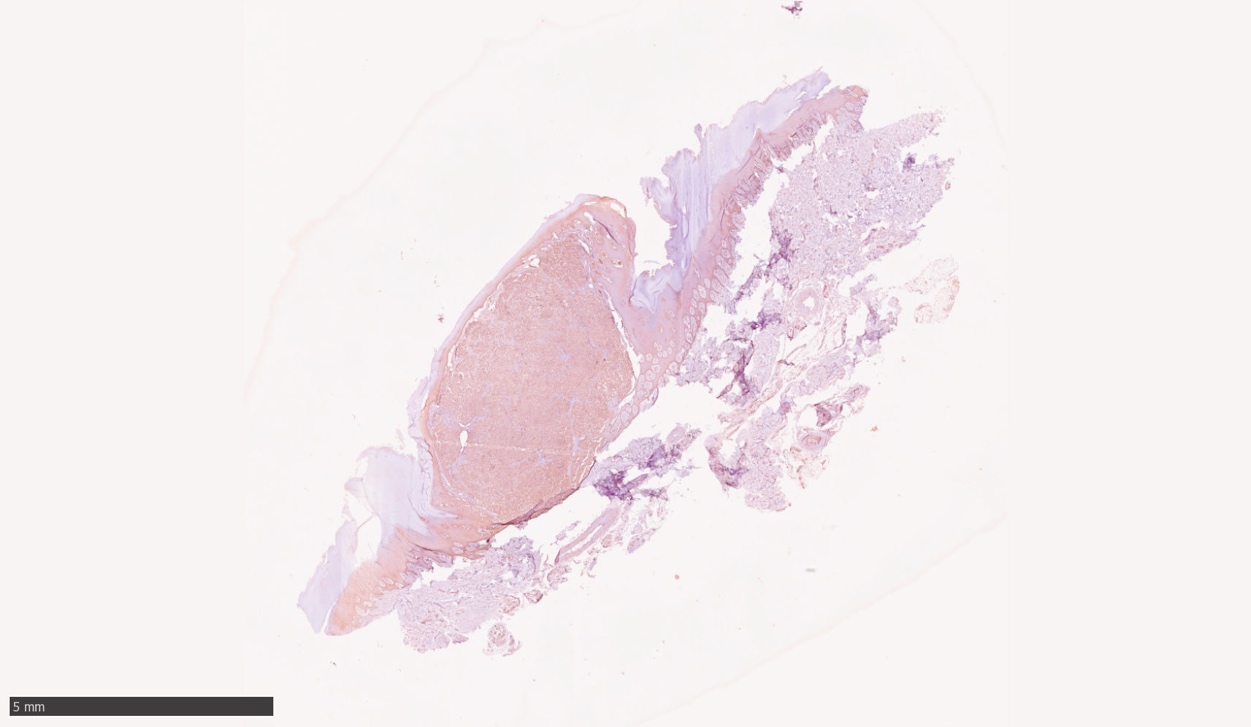


22-7459_NLRP1
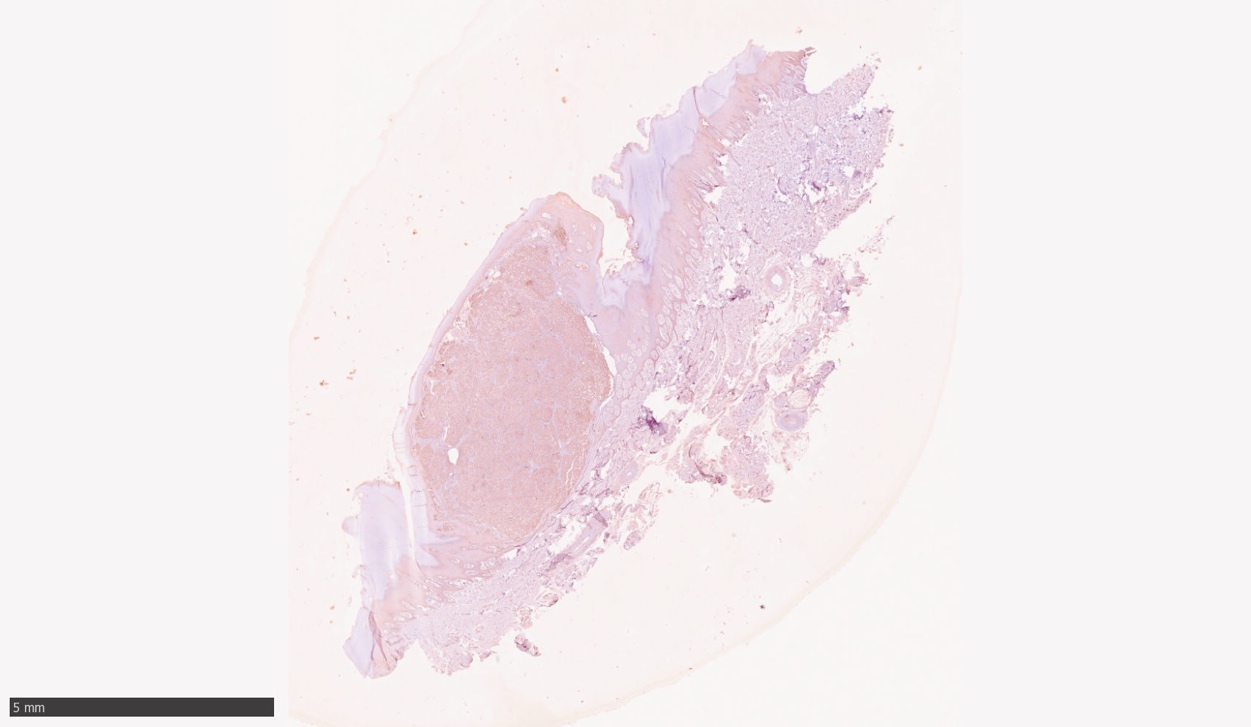


23-4488_CD8
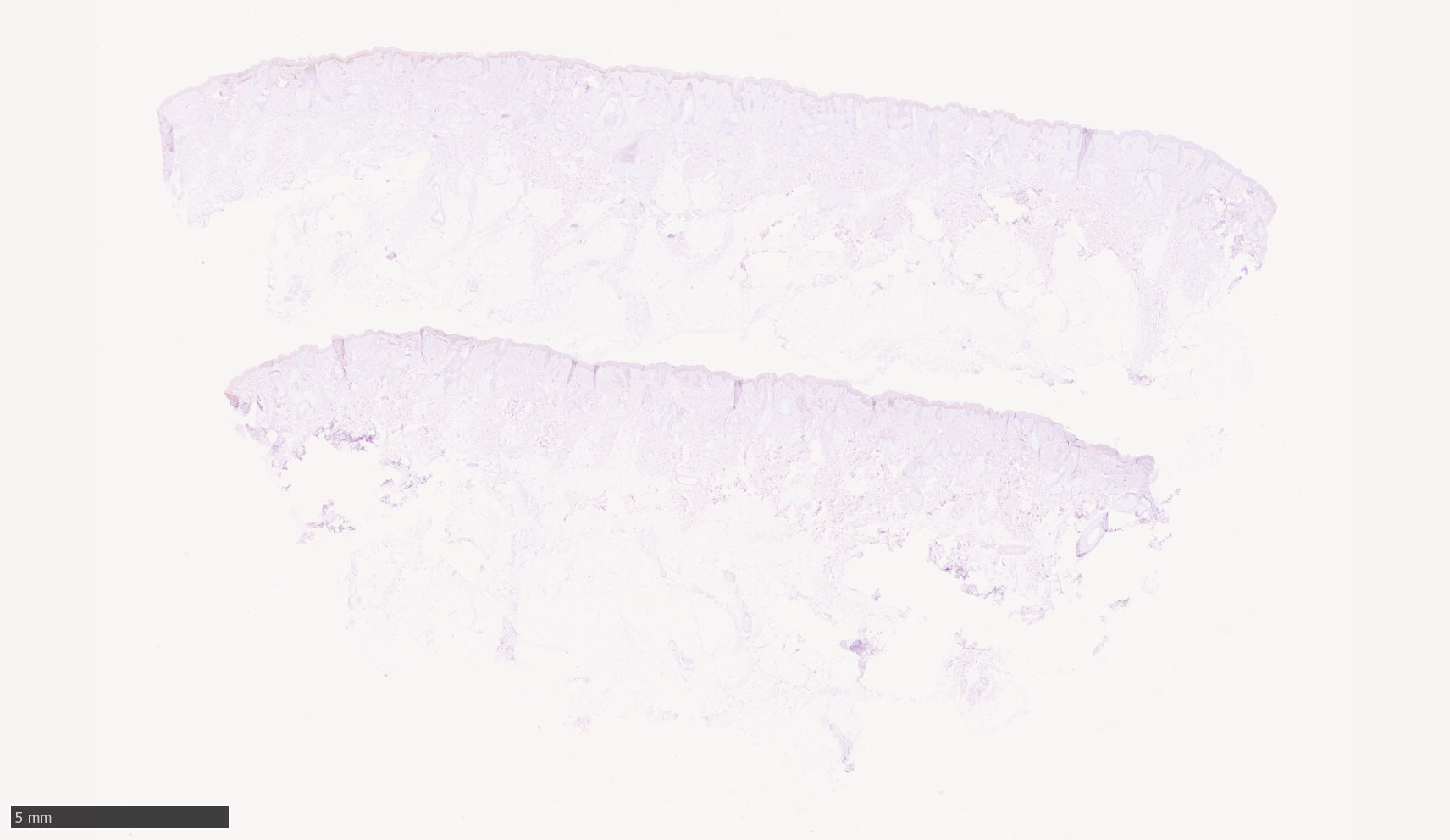


23-4488_CD57
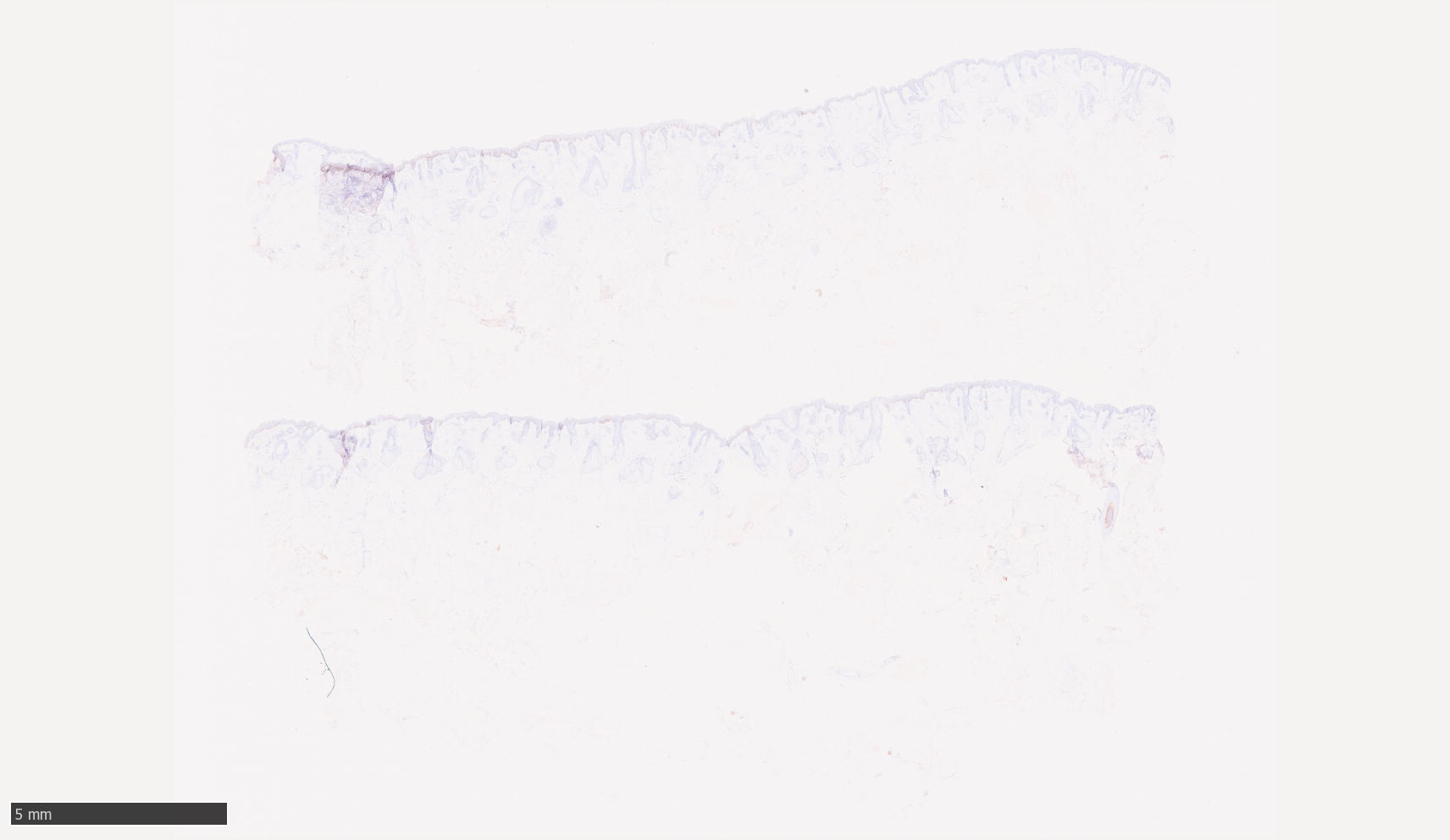


23-4488_CHMP4A
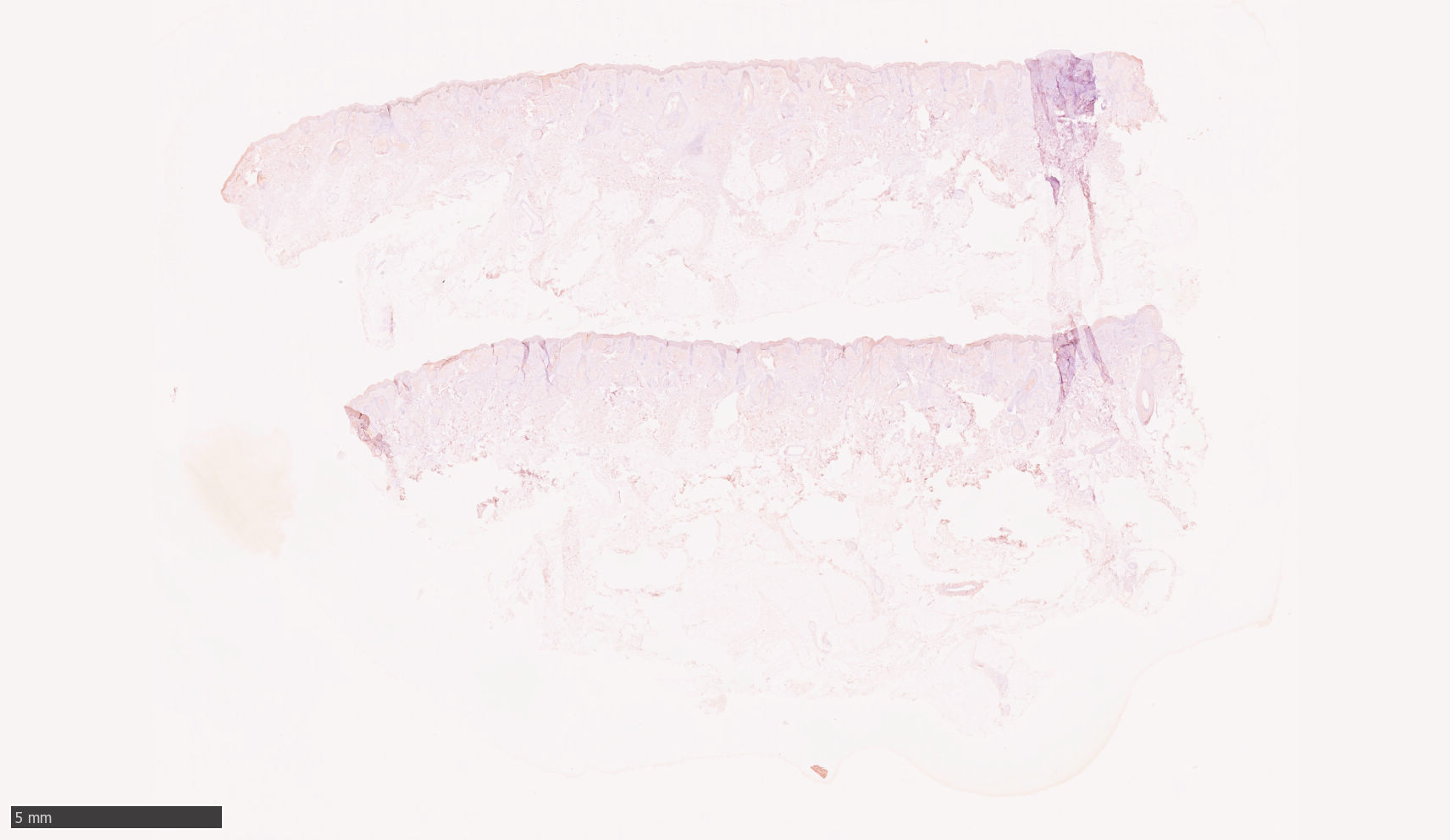


23-4488_GSDMB
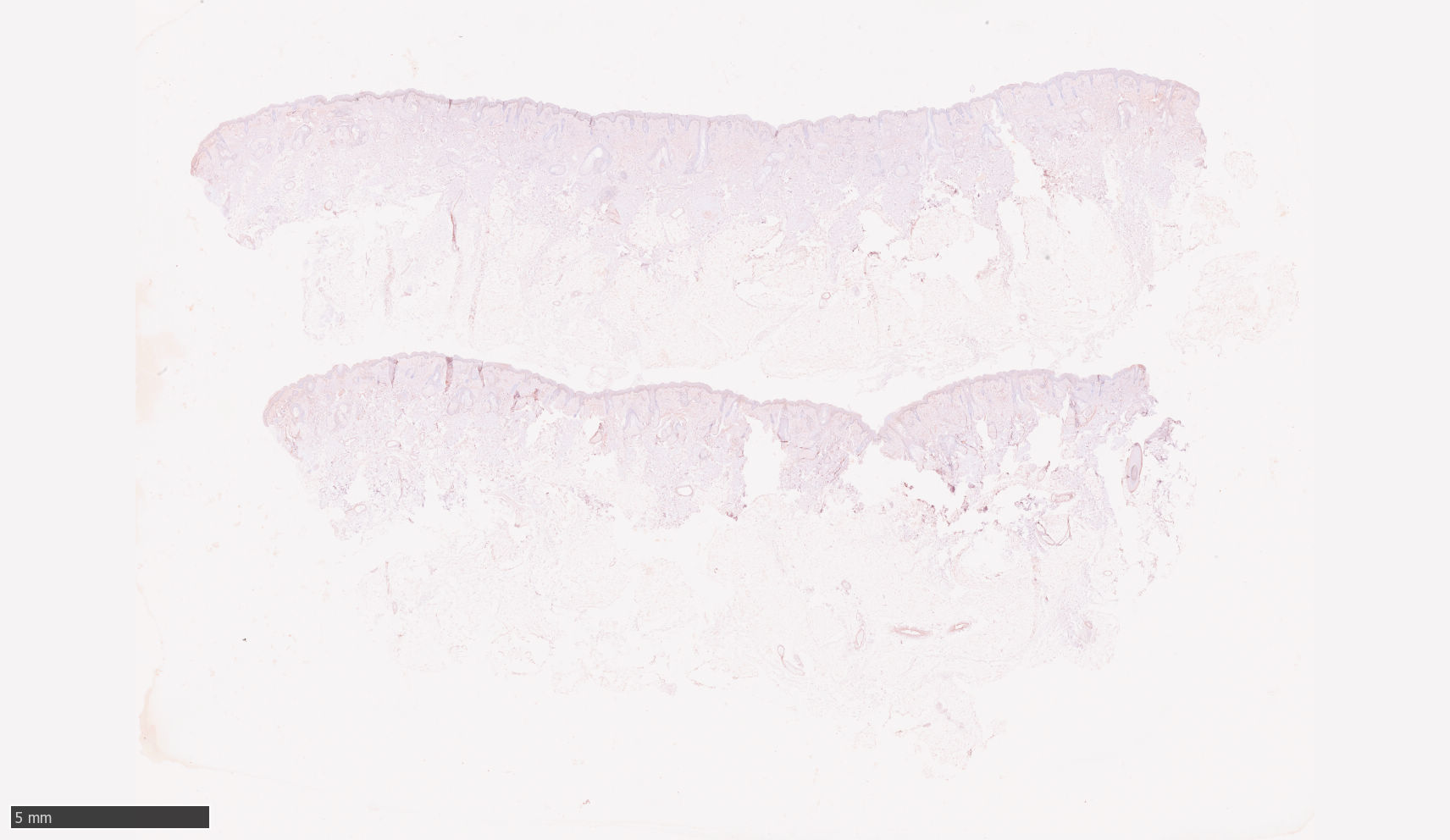


23-4488_GZMA
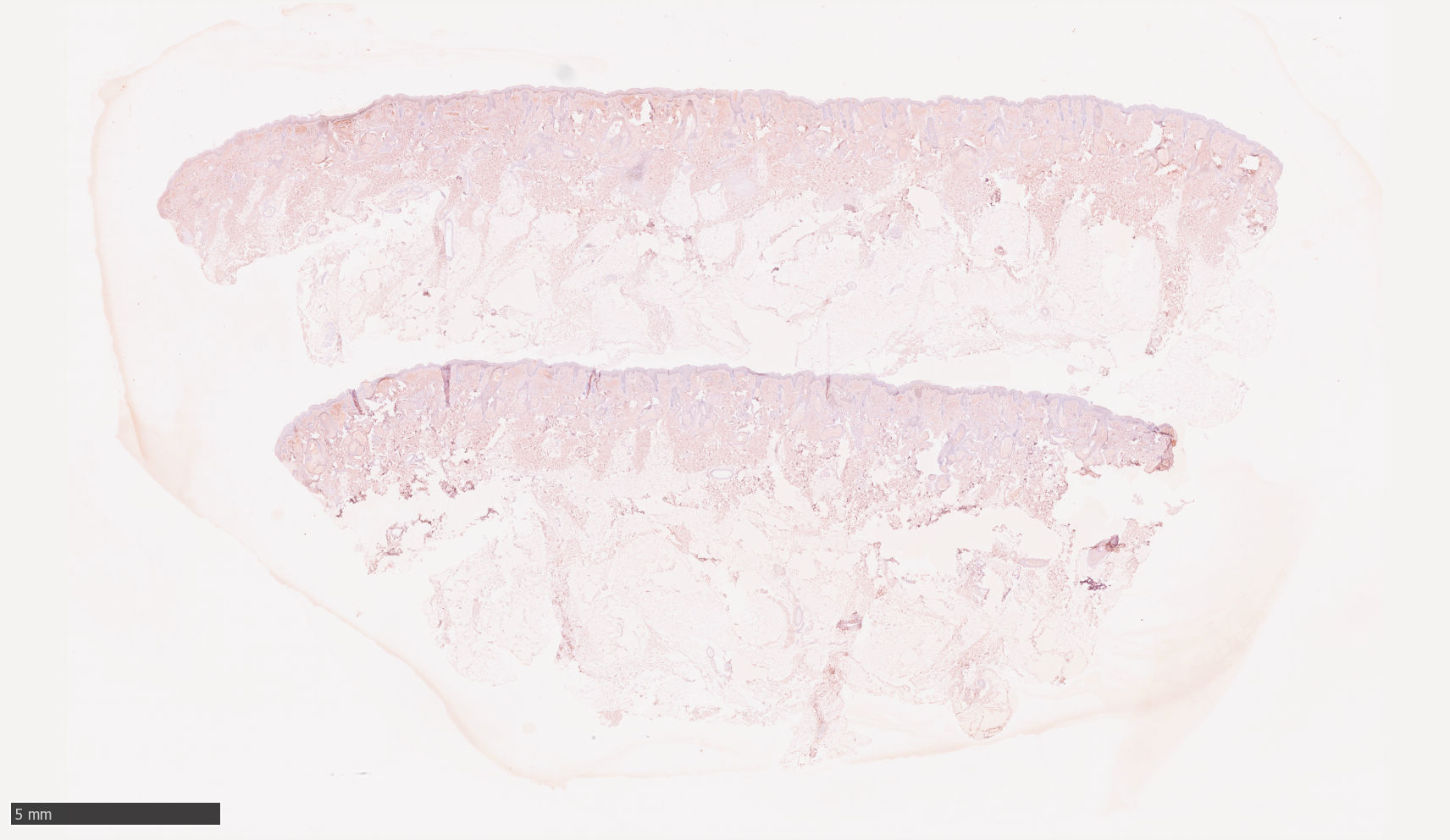


23-4488_IL18
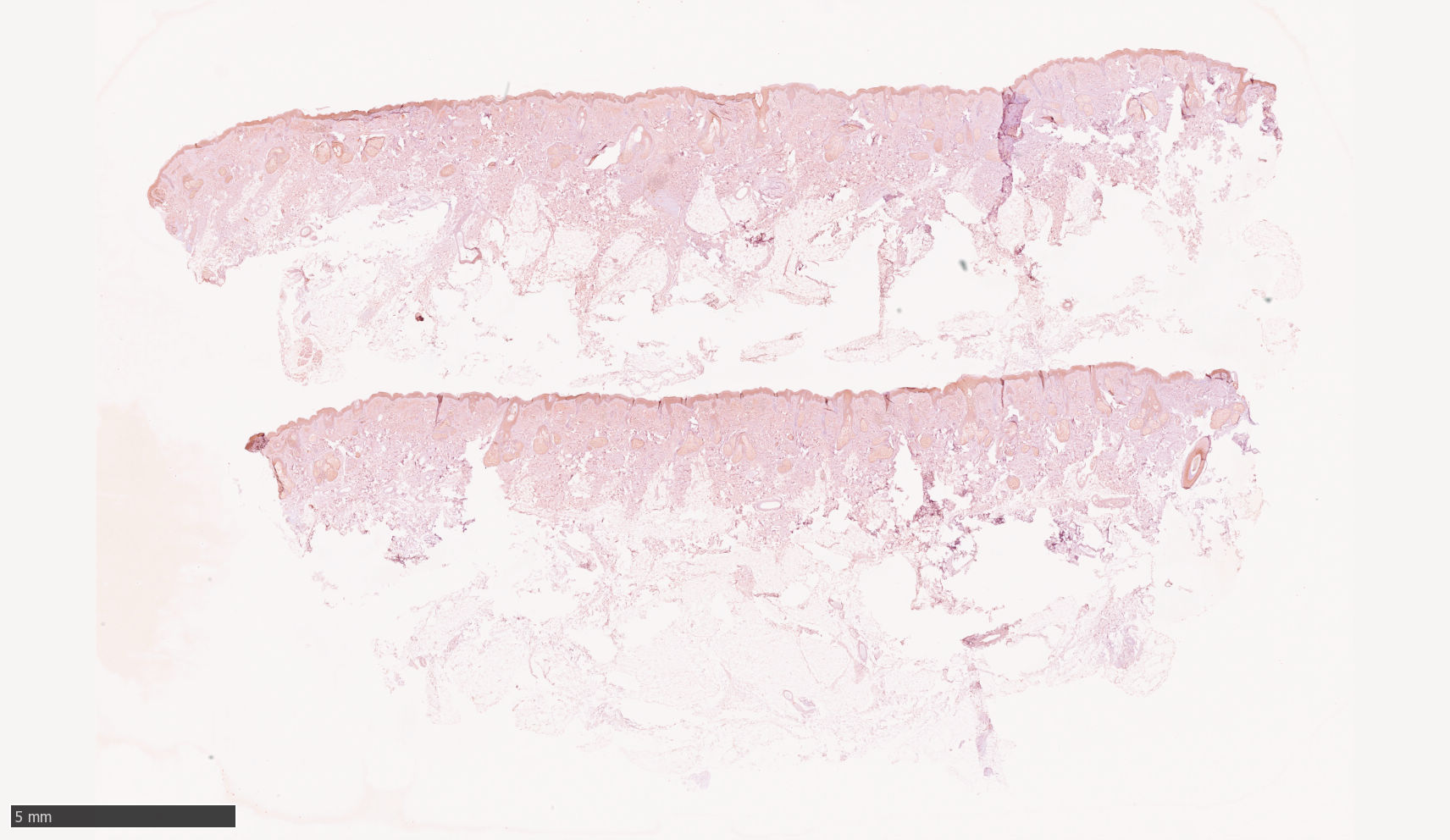


23-4488_NLRP1
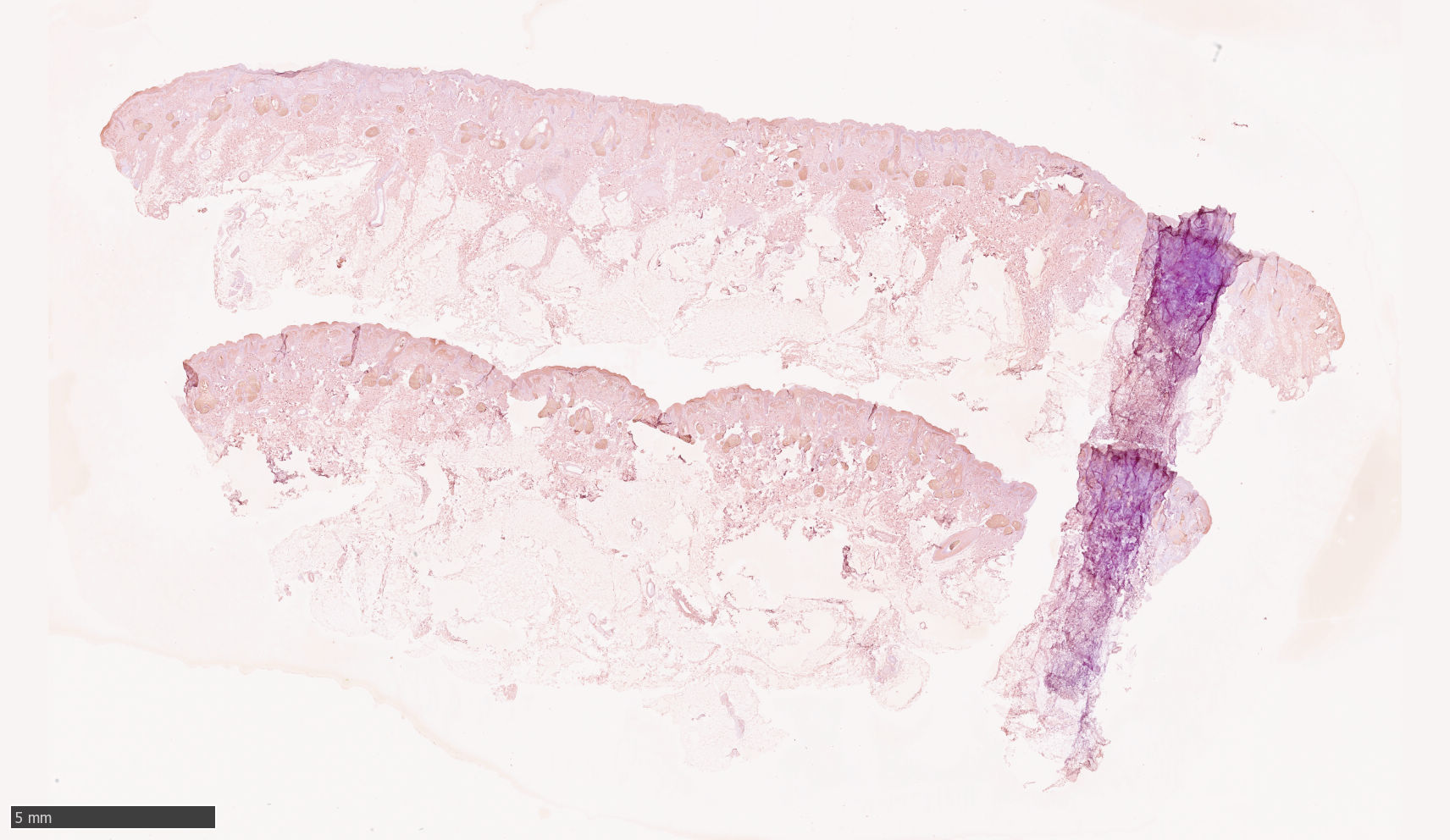


23-5303_CD8
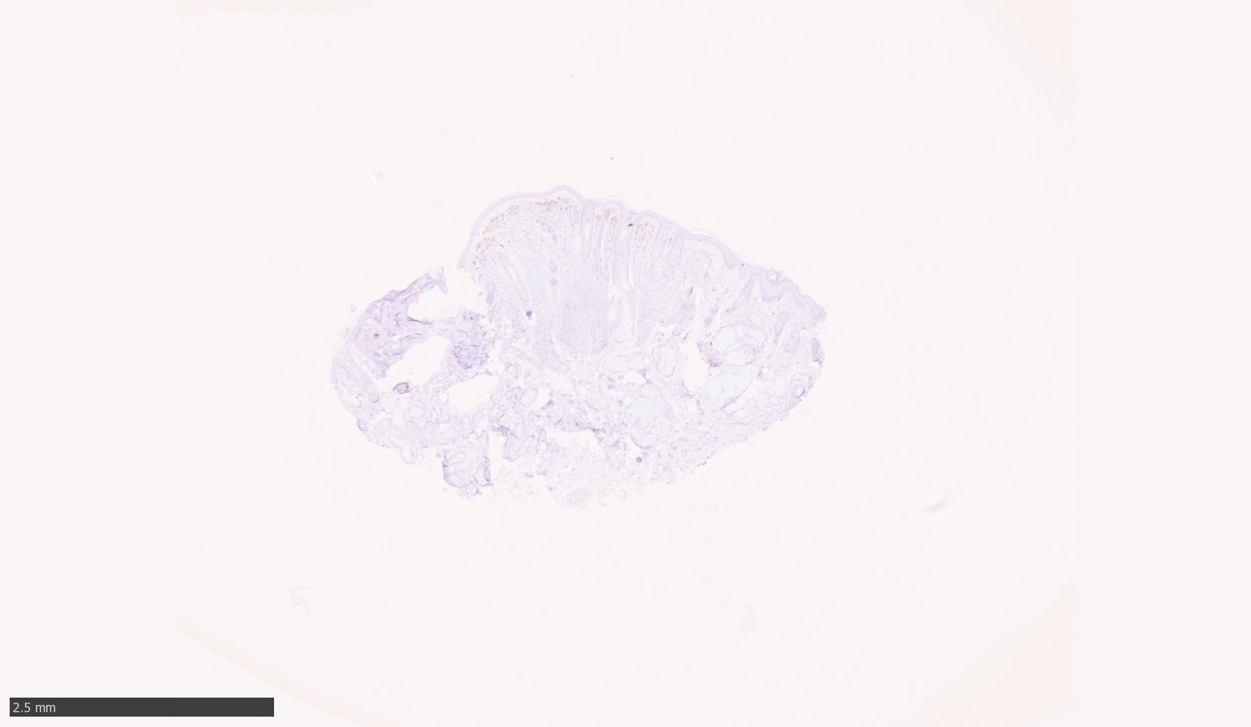


23-5303_CD57
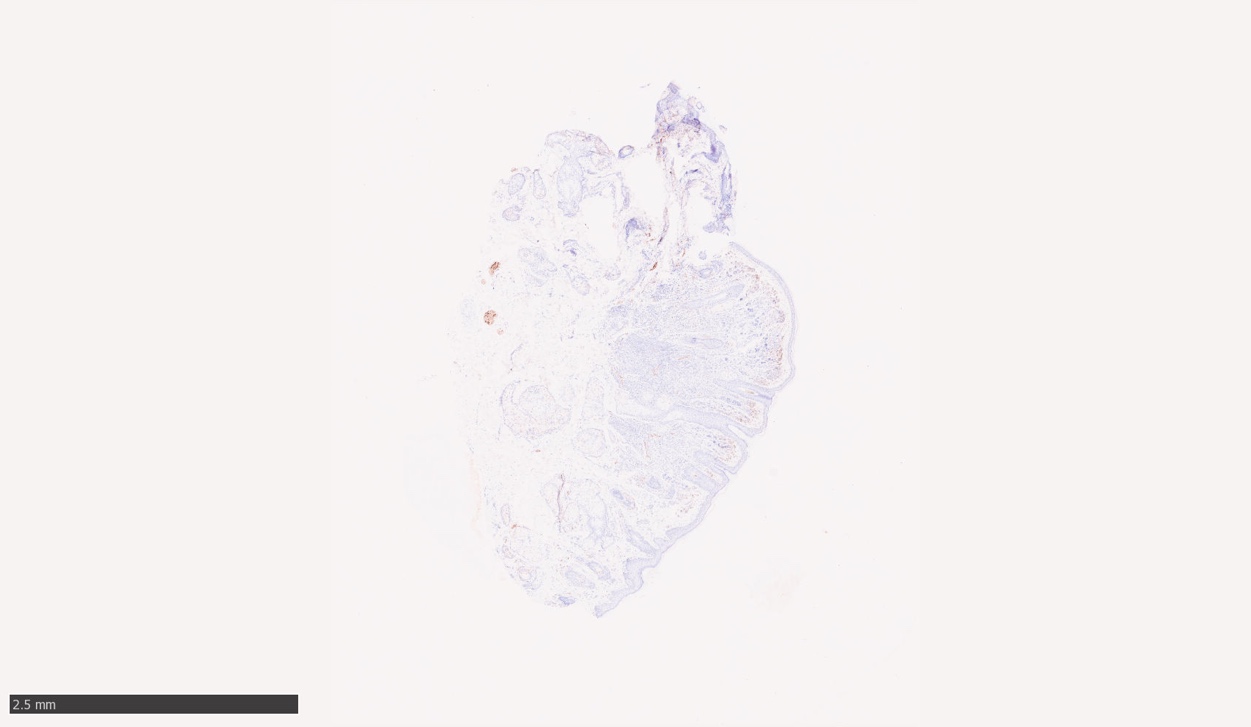


23-5303_CHMP4A
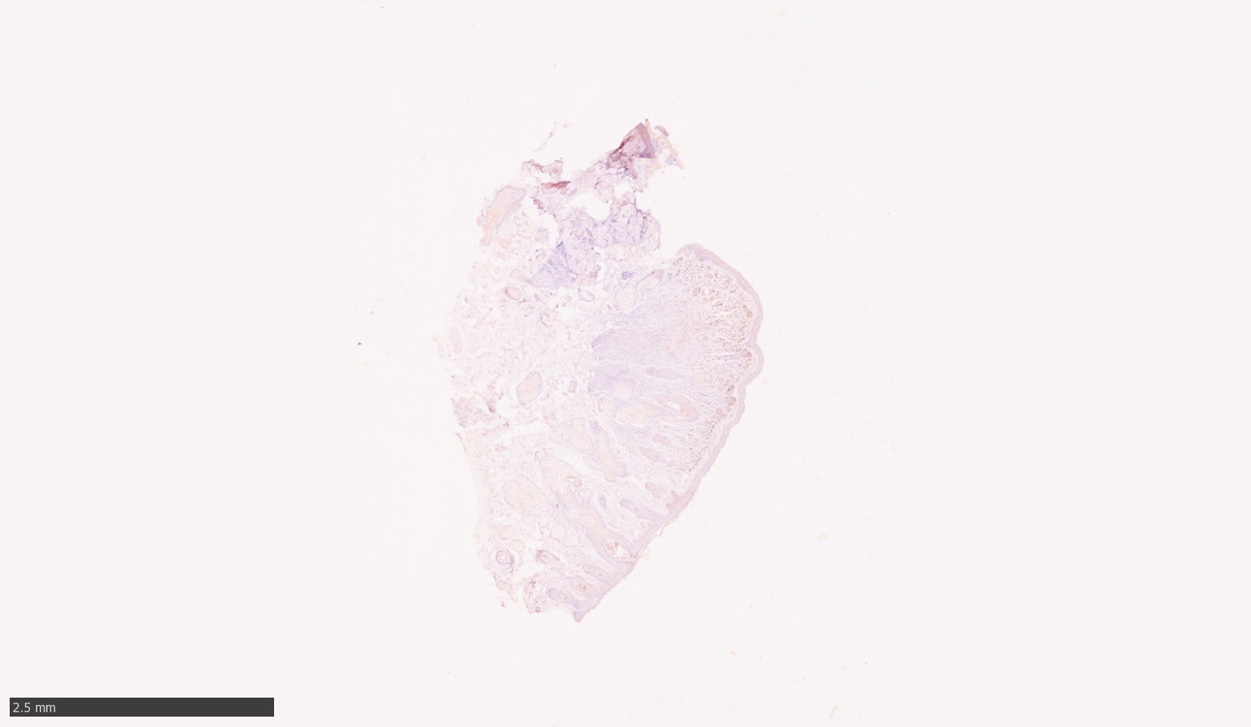


23-5303_GSDMB
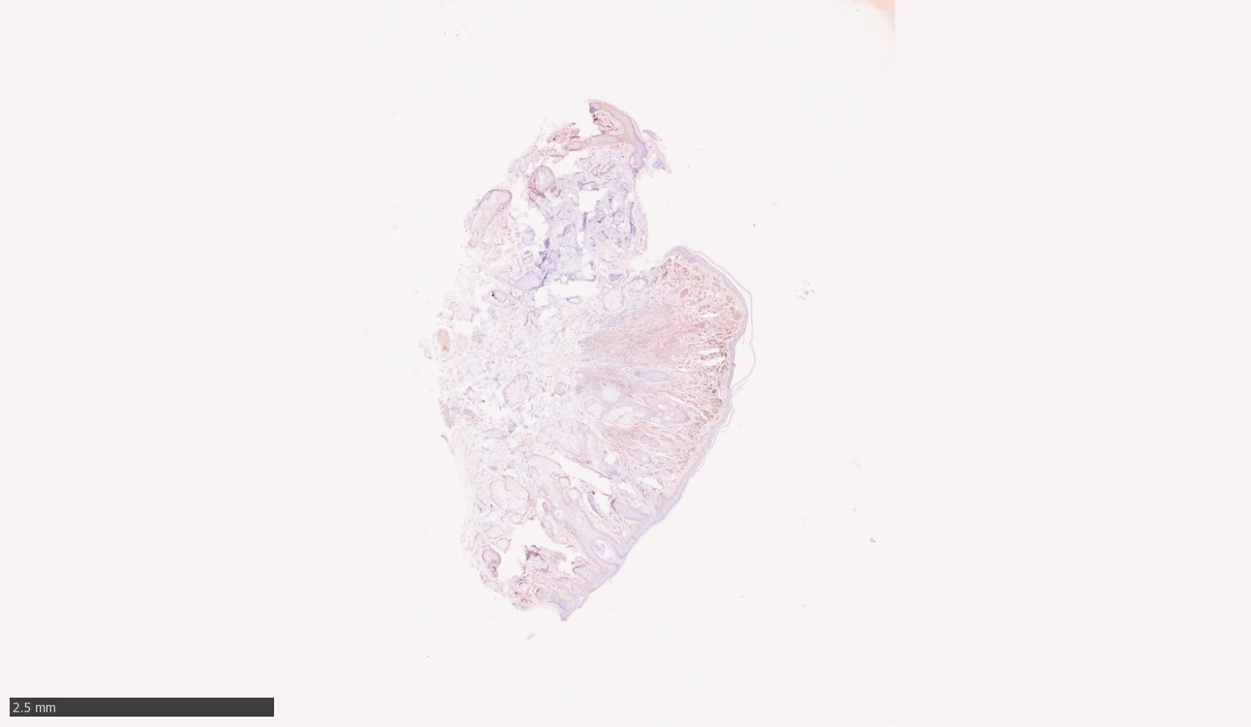


23-5303_GZMA
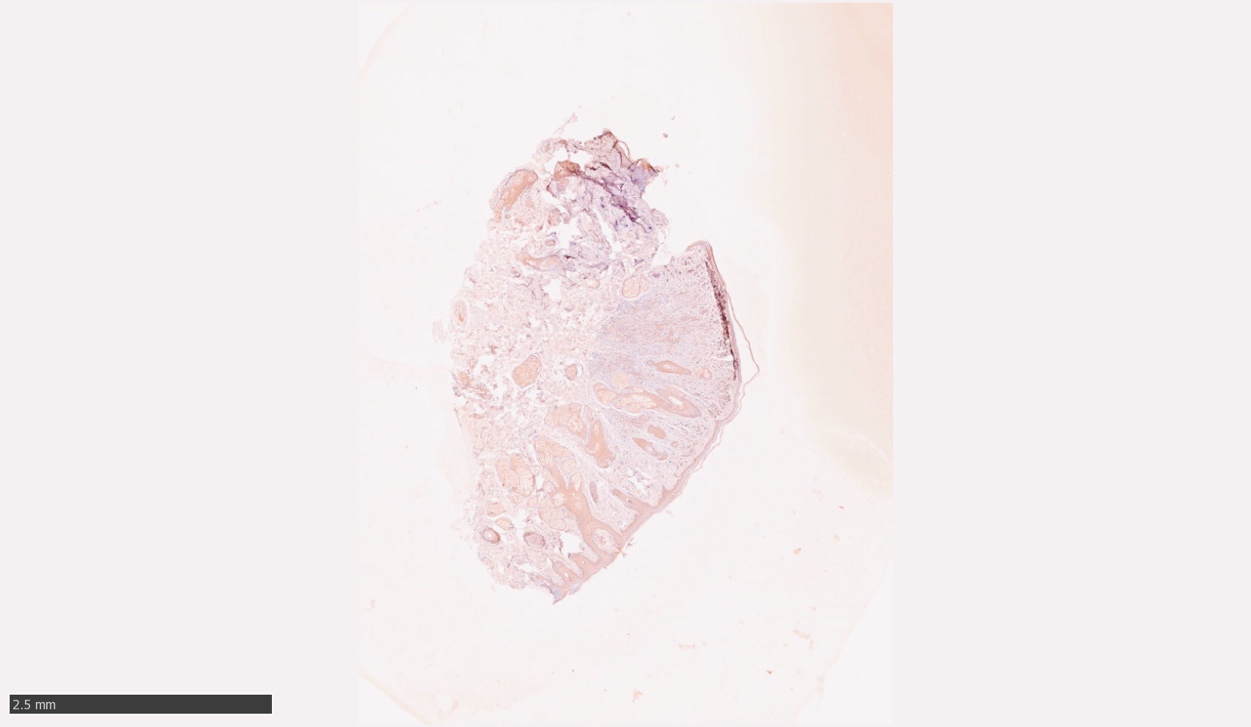


23-5303_IL18
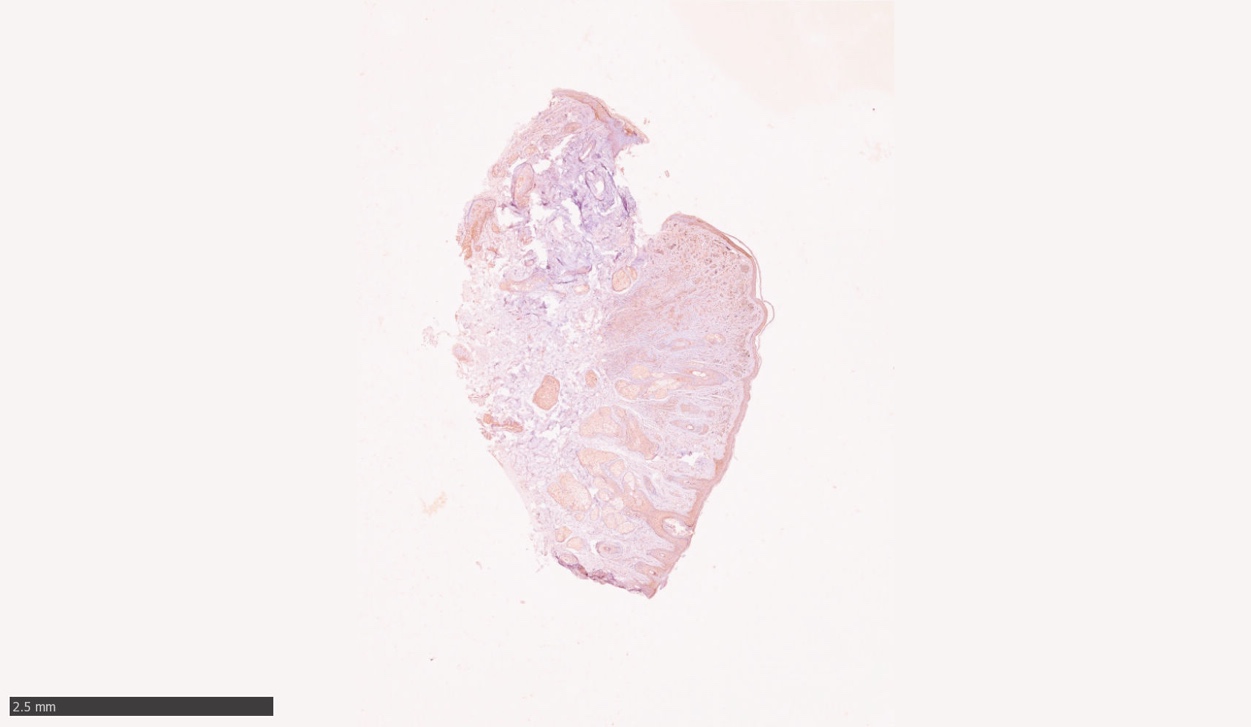


23-5303_NLRP1
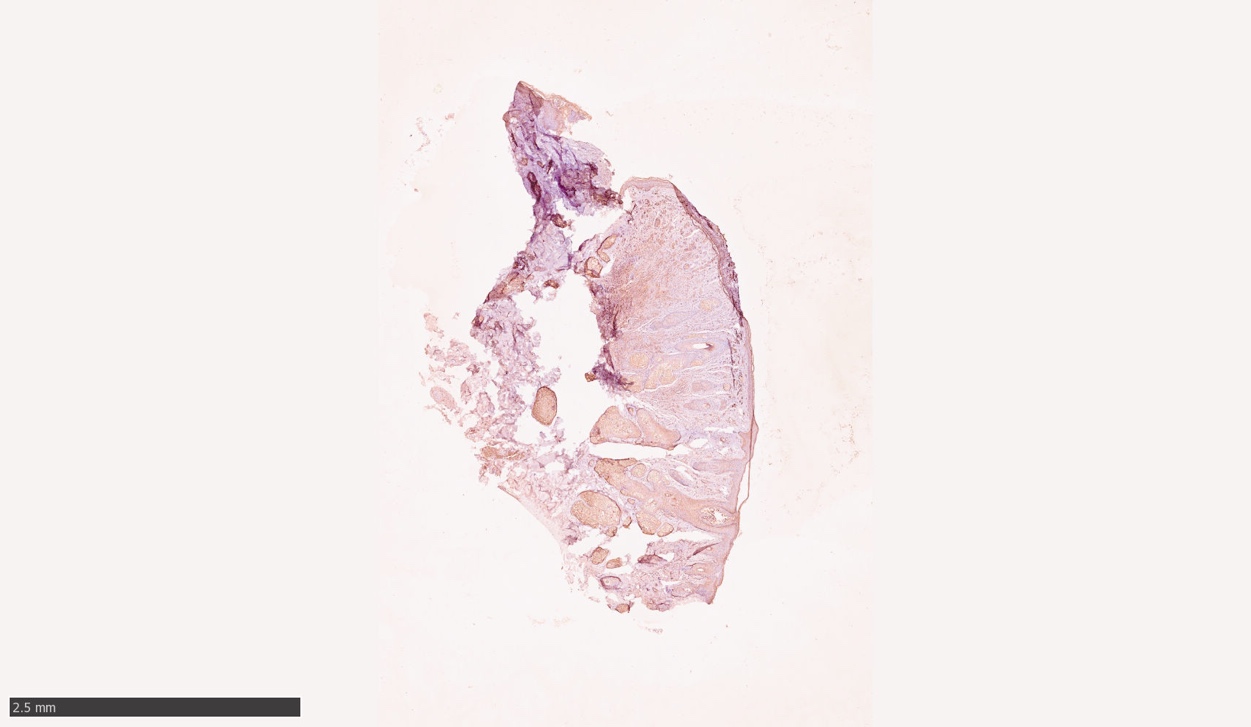


D23-5304_CD8
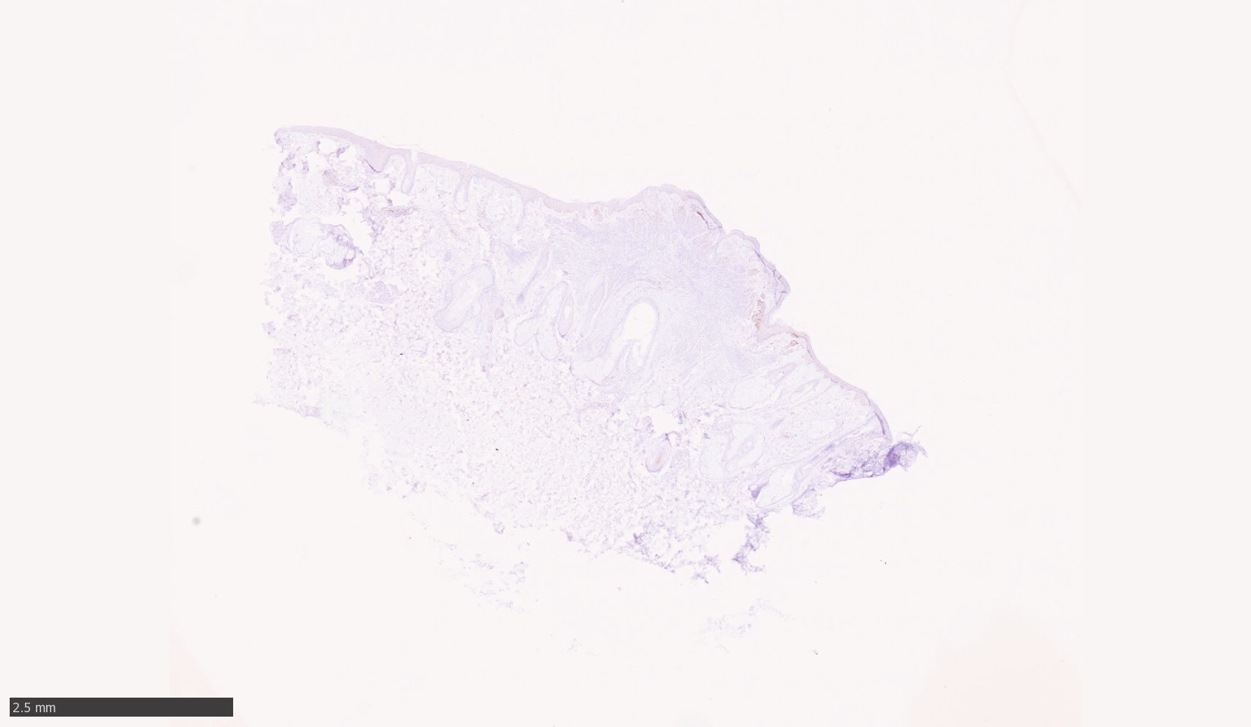


D23-5304_CD57

D23-5304_CHMP4A

D23-5304_GSDMB

D23-5304_GZMA

D23-5304_IL18

D23-5304_NLRP1

Multiplex immunofluorescence uncropped figure

raw 7503 cd57

raw 7503 cd8

raw 7459 cd57

raw 7459 cd8

raw 7188 cd57

raw 7188 cd8

raw 5304 cd57

raw 5303 cd57

raw 4582 cd57

raw 4582 cd8

raw 4488 cd57

raw 4488 cd8

raw 1261 cd57

raw 1261 cd8

raw 23-5304 cd8

raw 23-5303 cd8

R code

library(Seurat)

library(harmony)

library(ggplot2)

folders<- list.files("../melanoma")

sceList = lapply(folders,function(folder){

CreateSeuratObject(counts =Read10X(folder),

project =folder )

})

sce.big1 <- merge(sceList[[1]],

y = c(sceList[[2]],sceList[[3]],sceList[[4]],sceList[[5]]), add.cell.ids = folders)

#GSE215120

folders<- list.files("/home/zyy/project/20230304cnv_singlecell/GSE215120")

sceList = lapply(folders,function(folder){

CreateSeuratObject(counts =Read10X_h5(paste0("/home/zyy/project/20230304cnv_singlecell/GSE215120/",folder),use.names = T), project = substr(folder,1,10) )

})

sce.big2 <- merge(sceList[[1]],

y = c(sceList[[2]],sceList[[3]],sceList[[4]],sceList[[5]],

sceList[[6]],sceList[[7]],sceList[[8]],sceList[[9]],sceList[[10]],sceList[[11]]))

sce.big<- merge(sce.big1,sce.big2)

sce.big[["percent.mt"]] <- PercentageFeatureSet(sce.big, pattern = "^MT-")

sce.big[["percent.rb"]] <- PercentageFeatureSet(sce.big, pattern ="^RP[SL]")

minGene=200

maxGene=5000

pctMT=25

sce.filter <- subset(sce.big,subset = nFeature_RNA > minGene & nFeature_RNA < maxGene & percent.mt < pctMT)#103872

Seu_obj <- NormalizeData(sce.filter)

Seu_obj=FindVariableFeatures(Seu_obj, selection.method = "vst", nfeatures = 2000)

Seu_obj <- ScaleData(Seu_obj, features = VariableFeatures(Seu_obj))

Seu_obj <- RunPCA(Seu_obj)

rds1<- Seu_obj[,Seu_obj@meta.data$orig.ident %in% c("GSM5474333", "GSM5474334" ,"GSM5474335")]

Seu_obj= RunHarmony(rds1,

group.by.vars="orig.ident",

dims.use=1:30,

plot_convergence = TRUE)

Seu_obj <- FindNeighbors(Seu_obj,reduction="harmony", dims = 1:30)

Seu_obj<- FindClusters(Seu_obj,resolution = 0.5)

Seu_obj = RunTSNE(Seu_obj, dims = 1:30, reduction = "harmony", reduction.name = "tsne")

Seu_obj1<- Seu_obj

rds<- Seu_obj1

rds2<- Seu_obj[,!Seu_obj@meta.data$orig.ident %in% c("GSM5474333", "GSM5474334" ,"GSM5474335")]

Seu_obj= RunHarmony(rds2,

group.by.vars="orig.ident",

dims.use=1:30,

plot_convergence = TRUE)

Seu_obj <- FindNeighbors(Seu_obj,reduction="harmony", dims = 1:30)

Seu_obj<- FindClusters(Seu_obj,resolution = 0.7)

Seu_obj = RunTSNE(Seu_obj, dims = 1:30, reduction = "harmony", reduction.name = "tsne")

Seu_obj2<- Seu_obj

rds<- Seu_obj2

saveRDS(rds,file="tumor.rds")

plot5 =DimPlot(rds, reduction = "tsne", label=T)

ggsave("tsne_tissue2.png",plot=plot5)

p5 <- VlnPlot(rds, features = c("MLANA", "PMEL", "MITF", "DCT"))

ggsave("tumor1.png",plot=p5,width=9,height=6)

p55 <- FeaturePlot(rds,features = c("MLANA", "PMEL", "MITF", "DCT"))

ggsave("tumor2.png",plot=p55,width=6,height=6)

h2 <- VlnPlot(rds,features=c("VWF","CDH5","ENG","CLDN5","CD31","PECAM1","FLT1"))

ggsave("Endo13.png",plot=h2,width=9,height=6)

h3 <- FeaturePlot(rds,features=c("VWF","CDH5","ENG","CLDN5","CD31","PECAM1","FLT1"))

ggsave("Endo2.png",plot=h3,width=9,height=9)

h1 <- VlnPlot(rds,features=c("PDGFRA","PDGFRB","COL1A1","FAP","PDPN"))

ggsave("CAF14.png",plot=h1,width=9,height=6)

h2 <- FeaturePlot(rds,features=c("PDGFRA","PDGFRB","COL1A1","FAP","PDPN"))

ggsave("CAF2.png",plot=h2,width=6,height=9)

pe <- VlnPlot(rds,features=c("PDGFRB","MCAM","RGS5","CSPG4"))

ggsave("per1.png",plot=pe,width=9,height=6)

pe2 <- FeaturePlot(rds,features=c("PDGFRB","MCAM","RGS5","CSPG4"))

ggsave("per2.png",plot=pe2,width=6,height=6)

a1 <- VlnPlot(rds,features=c("CD3D","NKG7","CD56","GNLY","NCAM1","KLRD1","NCR1","XCL1"))

ggsave("NK.png",plot=a1,width=9,height=9)

a11 <- FeaturePlot(rds,features=c("CD3D","NKG7","CD56","GNLY","NCAM1","KLRD1","NCR1","XCL1"))

ggsave("NK2.png",plot=a11,width=9,height=9)

p4 <- VlnPlot(rds, features=c("CD68","CD163","CD14","CD204","CD206"))

ggsave("Myeloid1.png",plot=p4,width=9,height=3)

p44<- FeaturePlot(rds, features=c("CD68","CD163","CD14","CD204","CD206"))

ggsave("Myeloid2.png",plot=p44,width=6,height=6)

p <- VlnPlot(rds,features=c("CD2","CD3D","CD3E","CD3G","CD4","CD8A","CD8B"))

ggsave("T1.png",plot=p,width=9,height=9)

pp <-FeaturePlot(rds, features = c("CD2","CD3D","CD3E","CD3G","CD4","CD8A","CD8B"))

ggsave("T2.png",plot=pp,width=9,height=9)

p5 <- VlnPlot(rds, features = c("CD19","MS4A1","CD79B","CD79A"))##CD20原名MS4A1

ggsave("B1.png",plot=p5,width=9,height=6)

p55 <- FeaturePlot(rds,features = c("CD19","MS4A1","CD79B","CD79A"))

ggsave("B2.png",plot=p55,width=6,height=6)

p5 <- VlnPlot(rds, features = c("PTPRC","KLRB1","IL7R"))

ggsave("ilc1.png",plot=p5,width=10,height=4)

p55 <- FeaturePlot(rds,features = c("PTPRC","KLRB1","IL7R"))

ggsave("ilc2.png",plot=p55,width=6,height=6)

p5 <- VlnPlot(rds, features = c("KRT1","KRT5"))

ggsave("keratinocytes1.png",plot=p5,width=8,height=4)

p55 <- FeaturePlot(rds,features = c("KRT1","KRT5"))

ggsave("keratinocytes2.png",plot=p55,width=6,height=3)

p5 <- VlnPlot(rds, features = c("TAGLN","ACTA2"))

ggsave("SMC1.png",plot=p5,width=8,height=4)

p55 <- FeaturePlot(rds,features = c("TAGLN","ACTA2"))

ggsave("SMC2.png",plot=p55,width=6,height=3)

p5 <- VlnPlot(rds, features = c("LYZ"))

ggsave("phagocytes1.png",plot=p5,width=4,height=4)

p55 <- FeaturePlot(rds,features = c("LYZ"))

ggsave("phagocytes2.png",plot=p55,width=3,height=3)

p5 <- VlnPlot(rds, features = c("PECAM1","CLEC14A"))

ggsave("en1.png",plot=p5,width=8,height=4)

p55 <- FeaturePlot(rds,features = c("PECAM1","CLEC14A"))

ggsave("en2.png",plot=p55,width=6,height=3)

#########################

#

library(tidyverse)

library(ggrepel)

rds<- Seu_obj2

tsne = rds@reductions$tsne@cell.embeddings %>%

as.data.frame() %>%

cbind(cell_type =rds@meta.data$seurat_clusters)

cell_type_med <- tsne %>%

group_by(cell_type) %>%

summarise(

tSNE_1 = median( tSNE_1),

tSNE_2 = median( tSNE_2)

)

allcolour=c("#DC143C","#0000FF","#20B2AA","#FFA500","#9370DB","#98FB98","#F08080","#1E90FF","#7CFC00","#FFFF00","#FF1493","#0000CD","#008B8B","#FFE4B5","#8A2BE2","#228B22","#E9967A","#4682B4","#32CD32","#F0E68C","#FFFFE0","#EE82EE","#FF6347","#6A5ACD","#9932CC","#8B008B","#8B4513","#DEB887")

p <- ggplot(tsne,aes(x=tSNE_1 , y = tSNE_2 ,color = cell_type)) +

geom_point(size = 1 , alpha =0.3 ) +

scale_color_manual(values = allcolour)

p2 <- p +

theme(panel.grid.major = element_blank(), #

panel.grid.minor = element_blank(), #

panel.border = element_blank(), #

axis.title = element_blank(), #

axis.text = element_blank(), #

axis.ticks = element_blank(),

legend.position="none",

panel.background = element_rect(fill = 'white'), #

plot.background=element_rect(fill="white"))

p4 <- p2 +

geom_segment(aes(x = min(tsne$tSNE_1) , y = min(tsne$tSNE_2) ,

xend = min(tsne$tSNE_1) +7, yend = min(tsne$tSNE_2) ),

colour = "black", size=1,arrow = arrow(length = unit(0.3,"cm")))+

geom_segment(aes(x = min(tsne$tSNE_1) , y = min(tsne$tSNE_2) ,

xend = min(tsne$tSNE_1) , yend = min(tsne$tSNE_2) +7),

colour = "black", size=1,arrow = arrow(length = unit(0.3,"cm"))) +

annotate("text", x = min(tsne$tSNE_1) +2.5, y = min(tsne$tSNE_2) -1, label = "tSNE_1",

color="black",size = 3 ) +

annotate("text", x = min(tsne$tSNE_1) -1, y = min(tsne$tSNE_2) + 2.5, label = "tSNE_2",

color="black",size = 3, angle=90)

p5<- p4+geom_text_repel(aes(label=cell_type),data=cell_type_med, fontface="bold",col="black",size=5)

ggsave("tsne_tumor.pdf",plot=p5,width=6,height=5.5)

#####

rds<- Seu_obj1

tsne = rds@reductions$tsne@cell.embeddings %>%

as.data.frame() %>%

cbind(cell_type =rds@meta.data$seurat_clusters)

cell_type_med <- tsne %>%

group_by(cell_type) %>%

summarise(

tSNE_1 = median( tSNE_1),

tSNE_2 = median( tSNE_2)

)

allcolour=c("#DC143C","#0000FF","#20B2AA","#FFA500","#9370DB","#98FB98","#F08080","#1E90FF","#7CFC00","#FFFF00","#FF1493","#0000CD","#008B8B","#FFE4B5","#8A2BE2","#228B22","#E9967A","#4682B4","#32CD32","#F0E68C","#FFFFE0","#EE82EE","#FF6347","#6A5ACD","#9932CC","#8B008B","#8B4513","#DEB887")

p <- ggplot(tsne,aes(x=tSNE_1 , y = tSNE_2 ,color = cell_type)) +

geom_point(size = 1 , alpha =0.4) +

scale_color_manual(values = allcolour)

p2 <- p +

theme(panel.grid.major = element_blank(),

panel.grid.minor = element_blank(), #

panel.border = element_blank(), #

axis.title = element_blank(), #

axis.text = element_blank(), #

axis.ticks = element_blank(),

legend.position="none",

panel.background = element_rect(fill = 'white'), #

plot.background=element_rect(fill="white"))

p4 <- p2 +

geom_segment(aes(x = min(tsne$tSNE_1) , y = min(tsne$tSNE_2) ,

xend = min(tsne$tSNE_1) +7, yend = min(tsne$tSNE_2) ),

colour = "black", size=1,arrow = arrow(length = unit(0.3,"cm")))+

geom_segment(aes(x = min(tsne$tSNE_1) , y = min(tsne$tSNE_2) ,

xend = min(tsne$tSNE_1) , yend = min(tsne$tSNE_2) +7),

colour = "black", size=1,arrow = arrow(length = unit(0.3,"cm"))) +

annotate("text", x = min(tsne$tSNE_1) +4, y = min(tsne$tSNE_2) -1.5, label = "tSNE_1",

color="black",size = 3 ) +

annotate("text", x = min(tsne$tSNE_1) -1.5, y = min(tsne$tSNE_2) + 4, label = "tSNE_2",

color="black",size = 3, angle=90)

p5<- p4+geom_text_repel(aes(label=cell_type),data=cell_type_med, fontface="bold",col="black",size=5)

ggsave("tsne_normal.pdf",plot=p5,width=6,height=5.5)

####

rds<- Seu_obj2

rds<- rds[,rds@meta.data$seurat_clusters %in% c(2,3,16,17,24)]

Seu_obj<- rds

Seu_obj= RunHarmony(Seu_obj,

group.by.vars="orig.ident",

dims.use=1:30,

plot_convergence = TRUE)

Seu_obj <- FindNeighbors(Seu_obj,reduction="harmony", dims = 1:30)

Seu_obj<- FindClusters(Seu_obj,resolution = 0.5)

Seu_obj = RunTSNE(Seu_obj, dims = 1:30, reduction = "harmony", reduction.name = "tsne")

rds<- Seu_obj

Seu_obj3<- rds

saveRDS(Seu_obj3,file="immune_tumor.rds")

#####

rds<- Seu_obj3

tsne = rds@reductions$tsne@cell.embeddings %>%

as.data.frame() %>%

cbind(cell_type =rds@meta.data$seurat_clusters)

cell_type_med <- tsne %>%

group_by(cell_type) %>%

summarise(

tSNE_1 = median( tSNE_1),

tSNE_2 = median( tSNE_2)

)

allcolour=c("#DC143C","#0000FF","#20B2AA","#FFA500","#9370DB","#98FB98","#F08080","#1E90FF","#7CFC00","#FFFF00","#FF1493","#0000CD","#008B8B","#FFE4B5","#8A2BE2","#228B22","#E9967A","#4682B4","#32CD32","#F0E68C","#FFFFE0","#EE82EE","#FF6347","#6A5ACD","#9932CC","#8B008B","#8B4513","#DEB887")

p <- ggplot(tsne,aes(x=tSNE_1 , y = tSNE_2 ,color = cell_type)) +

geom_point(size = 1 , alpha =0.4) +

scale_color_manual(values = allcolour)

p2 <- p +

theme(panel.grid.major = element_blank(), #

panel.grid.minor = element_blank(), #

panel.border = element_blank(), #

axis.title = element_blank(), #

axis.text = element_blank(), #

axis.ticks = element_blank(),

legend.position="none",

panel.background = element_rect(fill = 'white'), #

plot.background=element_rect(fill="white"))

p4 <- p2 +

geom_segment(aes(x = min(tsne$tSNE_1) , y = min(tsne$tSNE_2) ,

xend = min(tsne$tSNE_1) +7, yend = min(tsne$tSNE_2) ),

colour = "black", size=1,arrow = arrow(length = unit(0.3,"cm")))+

geom_segment(aes(x = min(tsne$tSNE_1) , y = min(tsne$tSNE_2) ,

xend = min(tsne$tSNE_1) , yend = min(tsne$tSNE_2) +7),

colour = "black", size=1,arrow = arrow(length = unit(0.3,"cm"))) +

annotate("text", x = min(tsne$tSNE_1) +4, y = min(tsne$tSNE_2) -1.5, label = "tSNE_1",

color="black",size = 3 ) +

annotate("text", x = min(tsne$tSNE_1) -1.5, y = min(tsne$tSNE_2) + 4, label = "tSNE_2",

color="black",size = 3, angle=90)

p5<- p4+geom_text_repel(aes(label=cell_type),data=cell_type_med, fontface="bold",col="black",size=5)

ggsave("tsne_immune.pdf",plot=p5,width=6,height=5.5)

####

tumor<- [Seu_obj2@meta.data](mailto:Seu_obj@meta.data)

info<- data.frame(seurat_clusters=0:25,celltype=c("Melanoma cell","Melanoma cell","T cell","NK/T cell","Melanoma cell","Melanoma cell","Melanoma cell","Melanoma cell","Melanoma cell","Melanoma cell",

"Endothelial cell","Melanoma cell","Melanoma cell","Fibroblasts","Fibroblasts","Melanoma cell","Myeloid cell","B cell","Melanoma cell","Melanoma cell","Melanoma cell","Melanoma cell","Fibroblasts","Melanoma cell","T cell","Endothelial cell"))

tumor$cell<- rownames(tumor)

tumor<- merge(tumor,info,by="seurat_clusters")

tumor_percent<- data.frame()

for (i in unique(tumor$celltype)){

cc<- tumor[tumor$celltype==i,]

per<- nrow(cc)/nrow(tumor)

tumor_percent[i,1]<- per

}

tumor_percent$celltype<- rownames(tumor_percent)

tumor_percent$class<- "Tumor"

normal<- [Seu_obj1@meta.data](mailto:Seu_obj1@meta.data)

info<- data.frame(seurat_clusters=0:10,celltype=c("ILCs","NK cell",

"NK cell","Keratinocytes","Keratinocytes","ILCs","ILCs","Keratinocytes","B cell",

"Phagocytes","Keratinocytes"))

normal$cell<- rownames(normal)

normal<- merge(normal,info,by="seurat_clusters")

normal_percent<- data.frame()

for (i in unique(normal$celltype)){

cc<- normal[normal$celltype==i,]

per<- nrow(cc)/nrow(normal)

normal_percent[i,1]<- per

}

normal_percent$celltype<- rownames(normal_percent)

normal_percent$class<-"Normal"

percent<- rbind(tumor_percent,normal_percent)

ggplot(percent,aes(class,V1,fill=celltype))+

geom_bar(stat="identity",width=0.7)+

theme_classic()+

scale_fill_manual(values=c("#FF1493","#0000CD","#FFFFE0","#EE82EE","#FF6347","#6A5ACD","#9932CC","#8B008B","#8B4513","#DEB887","#20B2AA"))+

ylab("Ratio of cell types")+xlab("")+labs(fill="Cell types")+

theme(axis.text=element_text(color="black"),legend.position="bottom")

ggsave("bar.pdf",width=7,height=8)

####

rds<- Seu_obj2

pp <-FeaturePlot(rds, features = c("CD2","CD3E","CD4","CD8A"))

ggsave("Tcell_marker.pdf",plot=pp,width=6,height=6)

pp <-FeaturePlot(rds, features = c("CD19","MS4A1","CD79B","CD79A"))

ggsave("Bcell_marker.pdf",plot=pp,width=6,height=6)

pp <-FeaturePlot(rds, features = c("CD68","CD163"))

ggsave("myeloid_marker.pdf",plot=pp,width=6,height=3)

pp <-FeaturePlot(rds, features = c("PDGFRA","PDGFRB","COL1A1"))

ggsave("fibroblast_marker.pdf",plot=pp,width=6,height=6)

pp <-FeaturePlot(rds, features = c("CDH5","ENG","PECAM1"))

ggsave("endothelial_marker.pdf",plot=pp,width=6,height=6)

pp <-FeaturePlot(rds, features = c("MLANA", "PMEL"))

ggsave("melanomacell_marker.pdf",plot=pp,width=6,height=3)

a11 <- FeaturePlot(rds,features=c("CD3D","NKG7","GNLY","KLRD1"))

ggsave("NKT_marker.pdf",plot=a11,width=6,height=6)

####

pp <-FeaturePlot(rds, features = c("GSDMB"))

ggsave("GSDMB_marker.pdf",plot=pp,width=3,height=3)

pp <-FeaturePlot(rds, features = c("GZMA"))

ggsave("GZMA_marker.pdf",plot=pp,width=3,height=3)

pp <-FeaturePlot(rds, features = c("GZMA","GZMB","IL18", "NLRP1" ,"CHMP4A"))

ggsave("select_markers.pdf",plot=pp,width=6,height=9)

###

rds<- Seu_obj3

pp <-FeaturePlot(rds, features = c("CD2","CD3E","CD4","CD8A"))

ggsave("Tcell_marker.pdf",plot=pp,width=6,height=6)

pp <-FeaturePlot(rds, features = c("CD19","MS4A1","CD79B","CD79A"))

ggsave("Bcell_marker.pdf",plot=pp,width=6,height=6)

pp <-FeaturePlot(rds, features = c("CD68","CD163"))

ggsave("myeloid_marker.pdf",plot=pp,width=6,height=3)

pp <-FeaturePlot(rds, features = c("PDGFRA","PDGFRB","COL1A1"))

ggsave("fibroblast_marker.pdf",plot=pp,width=6,height=6)

pp <-FeaturePlot(rds, features = c("CDH5","ENG","PECAM1"))

ggsave("endothelial_marker.pdf",plot=pp,width=6,height=6)

a11 <- FeaturePlot(rds,features=c("CD3D","NKG7","GNLY","KLRD1"))

ggsave("NK_marker.pdf",plot=a11,width=6,height=6)

b2 <- FeaturePlot(rds, features=c("TNFRSF17"))

ggsave("plasma.pdf",plot=b2,width=3,height=3)

pp <-FeaturePlot(rds, features = c("GZMA","GZMB","GSDMB","IL18", "NLRP1" ,"CHMP4A"))

ggsave("select_markers.pdf",plot=pp,width=6,height=9)

#####

Seu_obj<- readRDS("tumor.rds")

harmony.markers <- FindAllMarkers(Seu_obj, only.pos = TRUE, min.pct = 0.25, logfc.threshold = 0.25)

write.csv(harmony.markers ,"tumor_diff_gene.csv")

Seu_obj<- readRDS("immune_tumor.rds")

harmony.markers <- FindAllMarkers(Seu_obj, only.pos = TRUE, min.pct = 0.25, logfc.threshold = 0.25)

write.csv(harmony.markers ,"immune_diff_gene.csv")

###

library(Seurat)

library(harmony)

library(ggplot2)

tumor<- readRDS("immune_tumor.rds")

cd8<- tumor[,tumor@meta.data$seurat_clusters %in% c(1,7)]

nk<- tumor[,tumor@meta.data$seurat_clusters %in% c(2)]

cd8p=4334/18295=0.2369

nkp=3985/18295=0.2178

normal<- readRDS("normal.rds")

nim<- normal[,normal@meta.data$seurat_clusters %in% c(0,1,2,5,6,8,9)]

nk<- normal[,normal@meta.data$seurat_clusters %in% c(1,2)]

nkp=4201/9124=0.4604

######

library(Seurat)

library(monocle)

rds<- readRDS("immune_tumor.rds")

harmony.markers1<- read.csv("immune_diff_gene.csv",head=T,row.names=1)

harmony.markers1<- harmony.markers1[abs(harmony.markers1$avg_log2FC)>0.5 &harmony.markers1$p_val_adj<0.05,]

expr=rds@assays$[RNA@data](mailto:RNA@data)

dd<- as.data.frame(expr)

dd2<- dd[rowSums(dd)>0,]

monocle.matrix=as.matrix(dd2)

data <- as(monocle.matrix, 'sparseMatrix')

monocle.sample=[rds@meta.data](mailto:rds@meta.data)

monocle.sample$Cluster<- paste0("cluster ",monocle.sample$seurat_clusters)

monocle.geneAnn=data.frame(gene_short_name = row.names(data), row.names = row.names(data))

monocle.markers=harmony.markers1

#

pd<-new("AnnotatedDataFrame", data = monocle.sample)

fd<-new("AnnotatedDataFrame", data = monocle.geneAnn)

cds <- newCellDataSet(data, phenoData = pd, featureData = fd)

#

cds <- estimateSizeFactors(cds)

cds <- estimateDispersions(cds)

cds <- setOrderingFilter(cds, as.vector(harmony.markers1$gene))

cds <- reduceDimension(cds, max_components = 2, reduction_method = 'DDRTree')

cds <- orderCells(cds)

saveRDS(cds,file="im_cds.rds")

#

library(RColorBrewer)

library(ggplot2)

cds$Cluster<- factor(cds$Cluster,levels=paste0("cluster ",0:12))

pdf(file="im.trajectory.pdf",width=6.5,height=6.9)

plot_cell_trajectory(cds, color_by ="Cluster",show_branch_points=F) +

facet_wrap(~Cluster,nrow=4)+

theme_void()+theme(legend.position="none")+

scale_color_manual(values =c("#1B9E77",brewer.pal(12,"Paired")))

dev.off()

pdf("pseudotime.pdf",width = 4.5,height = 4)

plot_cell_trajectory(cds,color_by="Pseudotime", size=1,show_backbone=TRUE,

show_branch_points=F)

dev.off()

###

rds<- readRDS("tumor.rds")

gene<- c("CD19","MS4A1","CD79B","CD79A","CDH5","ENG","PECAM1","PDGFRA","PDGFRB","COL1A1","MLANA","PMEL","CD68","CD163","CD3D","NKG7","GNLY","KLRD1","CD2","CD3E","CD4","CD8A","GSDMB","GZMB","GZMA","NLRP1","IL18","CHMP4A")

for ( i in gene){

a1 <- VlnPlot(rds,features=i,pt.size=0)

ggsave(paste0(i,".pdf"),plot=a1,width=8,height=3)

}

rds2<- readRDS("immune_tumor.rds")

gene<- c("CD19","MS4A1","CD79B","CD79A","CD68","CD163","CD3D","NKG7","GNLY","KLRD1","TNFRSF17","CD2","CD3E","CD4","CD8A","GZMA","GZMB","GSDMB","IL18","NLRP1","CHMP4A")

for ( i in gene){

a1 <- VlnPlot(rds2,features=i,pt.size=0)

ggsave(paste0(i,".pdf"),plot=a1,width=6,height=3)

}
